# Supplementary material for: Comparing pharmaceutical company payments in the four UK countries: a cross-sectional and social network analysis
Source: BMJ Open. 2023 Mar 28;13(3):e061591. doi: 10.1136/bmjopen-2022-061591 (PMC10069501; doi:10.1136/bmjopen-2022-061591)
Supplement: Supplementary data [file bmjopen-2022-061591supp001.pdf]

Supplementary file contents:

**Supplementary File**	**Page**
Supplementary File 1. Payment types included in Disclosure UK and patient organisation codes applied to Disclosure UK codes	2
Supplementary File 2. Data integration flowchart	3
Supplementary File 3. Recipient category descriptions and examples	4-5
Supplementary File 4. Top 10% of donors in each country	6
Supplementary File 5. Visualised networks for England (a), Scotland (b), Wales (c), Northern Ireland (d)	7-10
Supplementary File 6. Top ten companies by degree centrality scores in each country	11
Supplementary File 7. Descriptive statistics for each recipient category	12-14
Supplementary File 8. Top 10 recipients in each country	15-16
Supplementary File 9. Post-hoc Bonferroni pairwise comparisons between countries of payments per category	17-19
Supplementary File 10. Post-hoc Bonferroni pairwise comparisons between countries of payment types	20

Rickard E, *et al.* *BMJ Open* 2023; 13:e061591. doi: 10.1136/bmjopen-2022-061591

Supplementary File 1. Payment types included in Disclosure UK and patient organisation codes applied to Disclosure UK codes

Payment type	Description of payment type	Patient organisation payments subsumed within the payment type
Contribution to costs of Events	Contribution to costs related to Events, through HCOs or Third Parties, including support to HCPs to attend Events, such as:<br>• Registration fees;<br>• Sponsorship agreements with HCOs or with Third Parties appointed by an HCO to manage an Event; and<br>• Travel and accommodation (EFPIA Code of Practice 2019, p. 30)	contributions to costs of events organised by recipients or third parties; travel, accommodation and registration fees
Donations and Grants to HCOs	Donations and Grants to HCOs that support healthcare, including donations and grants (either cash or benefits in kind) to institutions, organisations or associations that are comprised of HCPs and/or that provide healthcare (EFPIA Code of Practice 2019, p. 30)	donations; grants; corporate member, supporter, sponsor or partner; purchases and subscriptions from patient organisations; more than one distinct payment form; form of funding unclear; sponsorships
Fee for service and consultancy	Payments resulting from or related to contracts between Member Companies and HCOs under which such HCOs provide any type of services to a Member Company or any other type of funding not covered in the previous categories. Fees, on the one hand, and on the other hand payments relating to expenses agreed in the written agreement covering the activity will be disclosed as two separate amounts. (EFPIA Code of Practice 2019, p. 30)	fees for service and consultancy (including travel and accommodation); support, help and contributions
Joint working	The Department of Health defines joint working between the NHS and the pharmaceutical industry as situations where, for the benefit of patients, one or more pharmaceutical companies and the NHS pool skills, experience and/or resources for the joint development and implementation of patient centred projects and share a commitment to successful delivery. (ABPI Code of Practice 2015, Clause 20, p. 30)	n/a

Rickard E, *et al. BMJ Open* 2023; 13:e061591. doi: 10.1136/bmjopen-2022-061591

Supplementary File 2. Data integration flowchart

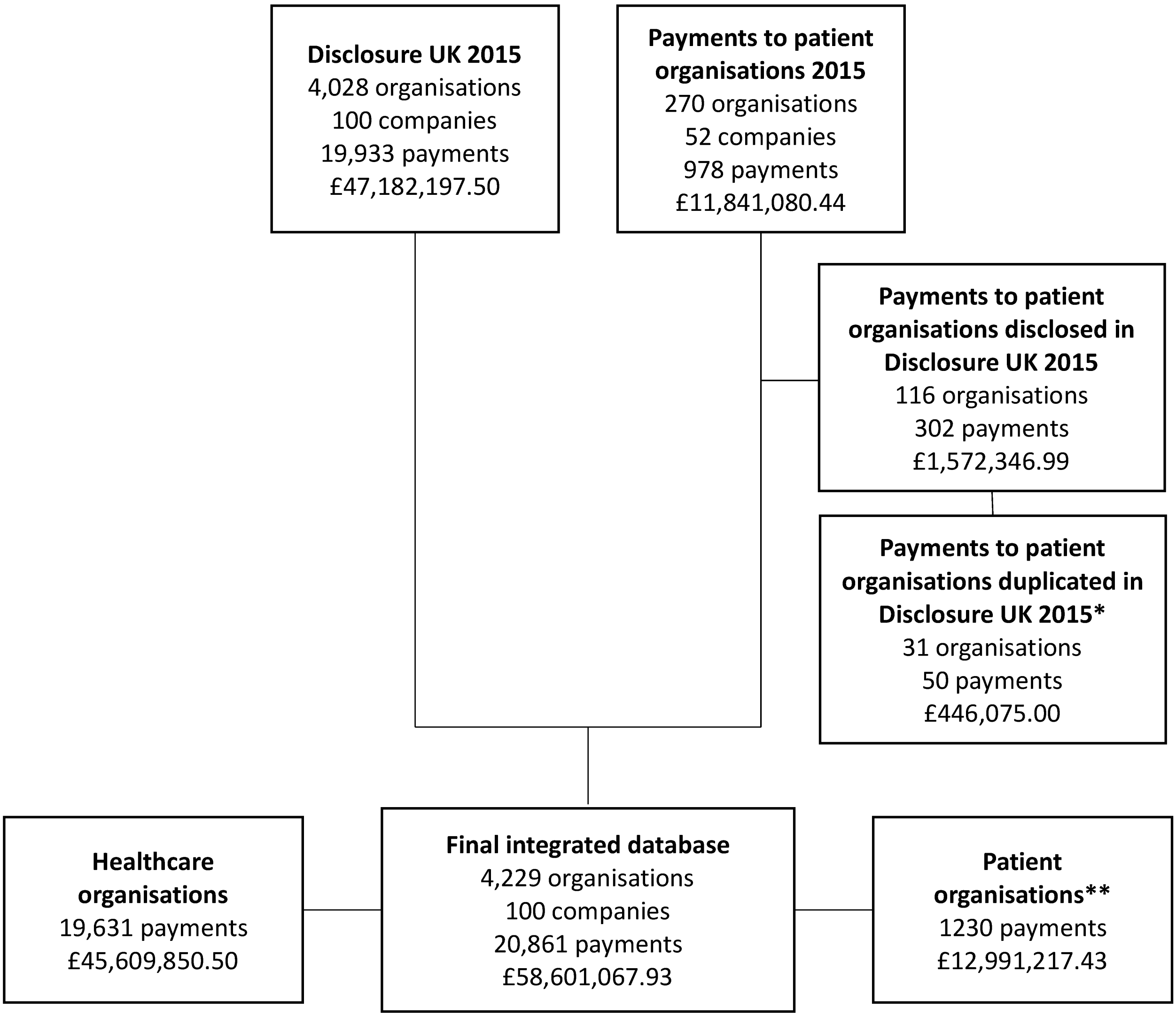

*This is the number and value of payments excluded to ensure no payment was counted twice

**During the cleaning process, a number of considerations took place to determine the final number and value of payments to patient organisations. Some duplicate payments were identified that were reported as multiple payments in one dataset and one payment in the other dataset (influencing the final number of payments). Approaches to VAT when reporting values also differed between the two datasets (influencing the final value of payments).

Rickard E, *et al. BMJ Open* 2023; 13:e061591. doi: 10.1136/bmjopen-2022-061591

Supplementary File 3. Recipient category descriptions and examples

Recipient category	Category description	Examples (country-specific examples where applicable)
Alternative providers of health services	Charities, not-for-profit companies, social enterprises and community interest companies providing health services	- social enterprise delivering primary or secondary care health services<br>- nursing or care home run by a community interest company (CIC)<br>- hospital, hospice, or nursing home with a charitable status
Education and research providers	Universities, charities, and noncommercial institutes undertaking research	- university<br>- research institute at a NHS organisation<br>- charity focusing on undertaking medical research
Formal bodies representing healthcare professionals or patients	Local medical, optical, optometric, or pharmaceutical committees and statutory bodies representing healthcare professionals or patients	- local medical committees (LMC)<br>- local optical or optometric committee (LOC)<br>*England*<br>- local pharmaceutical committee (LPC)
Charities and other third-sector organisations (excluding providers of health services, professional organisations, and patient organisations)	Organisations (not patient organisations) focusing on education, research, advocacy, and multipurpose organisations	- charitable trusts providing medical education events to healthcare professionals<br>- think tanks<br>- third-sector organisation (non-charity) or charity focused on funding medical research<br>- research institute registered as a charitable organisation
Healthcare commissioning, planning and regulatory organisations	Local, regional, and commissioning, planning, or regulatory organisations	- primary care trust (PCT)<br>- NHS Shared Business Services<br>- National Institute for Health and Care Excellence (NICE)<br>- Public Health England<br>*England*<br>- clinical commissioning group<br>- Locality group<br>- area prescribing committee<br>- local commissioning group<br>- NHS England<br>*Scotland*<br>- regional NHS board<br>- area pharmaceutical committee<br>*Wales*<br>- health board<br>- public health Wales

Rickard E, *et al. BMJ Open* 2023; 13:e061591. doi: 10.1136/bmjopen-2022-061591

		Northern Ireland<br>- health and social care board<br>- local commissioning group
Patient organisations	Organisations focusing on supporting education, research, advocacy, and multipurpose organisations	- multipurpose patient organisations<br>- organisations focused on providing patient support<br>- hospital charities
Private companies other than providers of health services	Providers of medical communications or training services, commercial or medical research services, and accountancy or consultancy services	- manufacturer or supplier of medical devices or technologies<br>- pharmacy wholesaler or distributor<br>- event management services<br>- journal or publishing company<br>- clinical or contract research organisation<br>- private laboratory
Private sector healthcare providers	Private clinics and hospitals, healthcare groups, and providers of dental, pharmacy, and optical services	- dental practice<br>- pharmacy or chemist<br>- opticians<br>- private clinic, surgery, practice, or hospital<br>- private company providing community health or social care services
Professional organisations	Organisations of medical professionals, other healthcare professionals, or non-healthcare professionals,	- organisation of medical professionals<br>- professional bodies responsible for setting standards of care and education for medical specialities<br>- royal college - medical professionals<br>- alliance or coalition of professional associations or groups<br>- professional organisation of pharmacists or pharmacy technicians
Public administration and providers of public services	Central UK government bodies, devolved administrations in Scotland, Wales, and Northern Ireland, and local authorities	- district, city, country, or borough council<br>- prison<br>- devolved administrations<br>- central government bodies
Public sector primary care providers	General practitioner surgeries, medical practice centres, groups of surgeries or medical practices, and healthcare or medical groups	- GP practice, surgery, medical practice or family practice<br>- health centre, medical centre or primary care centre<br>- group of surgeries or medical practices
Public sector secondary and tertiary care providers	NHS trusts, NHS hospitals, and networks and collaboratives of NHS organisations	- NHS hospital<br>- NHS Foundation Trust<br>- NHS rust<br>- strategic clinical network (SCN)<br>*Scotland*<br>- managed clinical network (MCN)

Rickard E, *et al. BMJ Open* 2023; 13:e061591. doi: 10.1136/bmjopen-2022-061591

Supplementary File 4. Top 10% of donors in each country

Country	Company (revenue ranking)	Payment value - £ (%)	Payments – n (%)
England	Pfizer (2)	5,292,130.74 (10.09)	1636 (8.99)
	Novartis (7)	3,564,500.43 (6.80)	460 (2.53)
	Bayer (8)	3,476,304.44 (6.63)	2110 (11.60)
	GlaxoSmithKline (4)	3,291,496.35 (6.28)	1076 (5.92)
	AstraZeneca (1)	2,779,000.54 (5.30)	1279 (7.03)
	Janssen-Cilag (10)	2,387,242.64 (4.55)	722 (3.97)
	UCB Pharma (30)	2,204,967.90 (4.20)	74 (0.41)
	Astellas Pharma (21)	2,044,050.60 (3.90)	311 (1.71)
	Roche (5)	1,931,651.77 (3.68)	173 (0.95)
	Biogen Idec (23)	1,886,879.26 (3.60)	83 (0.46)
	Top 10% total	28,858,224.67 (55.03)	7924 (43.56)
Scotland	Biogen Idec (24)	733,104.05 (20.09)	7 (0.51)
	Takeda UK (38)	274,952.71 (7.53)	25 (1.82)
	Pfizer (2)	250,859.45 (6.87)	143 (10.44)
	Bayer (8)	215,930.76 (5.92)	164 (11.97)
	Novartis (7)	199,703.97 (5.47)	48 (3.50)
	Bristol-Myers Squibb (23)	183,959.00 (5.04)	51 (3.72)
	AstraZeneca (1)	178,848.49 (4.90)	75 (5.47)
	Top 10% total	2,037,358.42 (55.82)	513 (37.45)
Wales	Pfizer (2)	284,719.57 (14.32)	102 (10.30)
	Roche (5)	230,090.90 (11.58)	10 (1.01)
	Novartis (7)	177,069.59 (8.91)	36 (3.64)
	AstraZeneca (1)	148,288.67 (7.46)	79 (7.98)
	Janssen-Cilag (10)	122,237.44 (6.15)	22 (2.22)
	Biogen (24)	112,428.62 (5.66)	7 (0.71)
	Top 10% total	1,074,834.80 (54.07)	256 (25.86)
Northern Ireland	Sanofi Aventis (13)	92,252.80 (17.81)	24 (7.72)
	Pfizer (2)	86,639.31 (16.73)	45 (14.47)
	Napp Pharmaceuticals (29)	83,252.29 (16.07)	45 (14.47)
	Bayer (8)	37,959.50 (7.33)	75 (24.12)
	Top 10% total	300,103.90 (57.94)	189 (60.77)

*Value as proportion of all payments in each country

Rickard E, *et al. BMJ Open* 2023; 13:e061591. doi: 10.1136/bmjopen-2022-061591

Supplementary File 5. Visualised networks for England (a), Scotland (b), Wales (c), Northern Ireland (d)

Supplementary File 5a. England's network

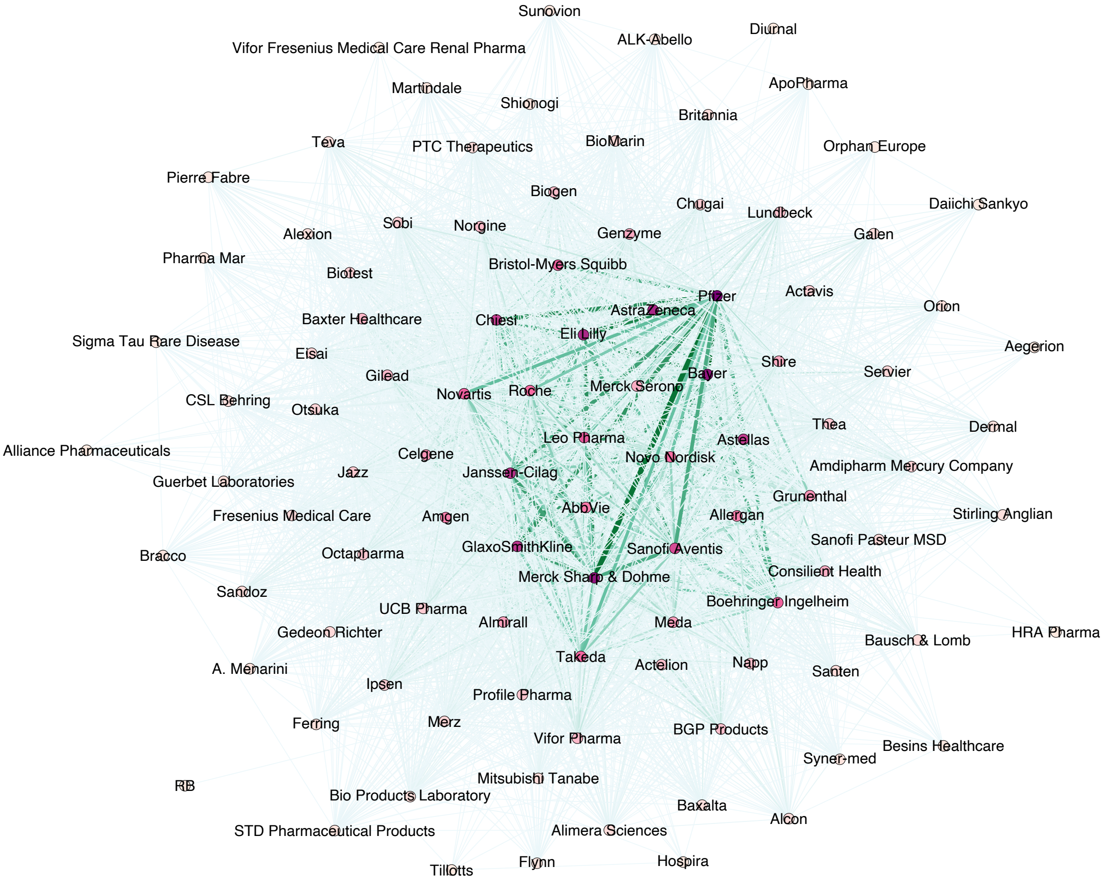

Supplementary File 5b. Scotland's network

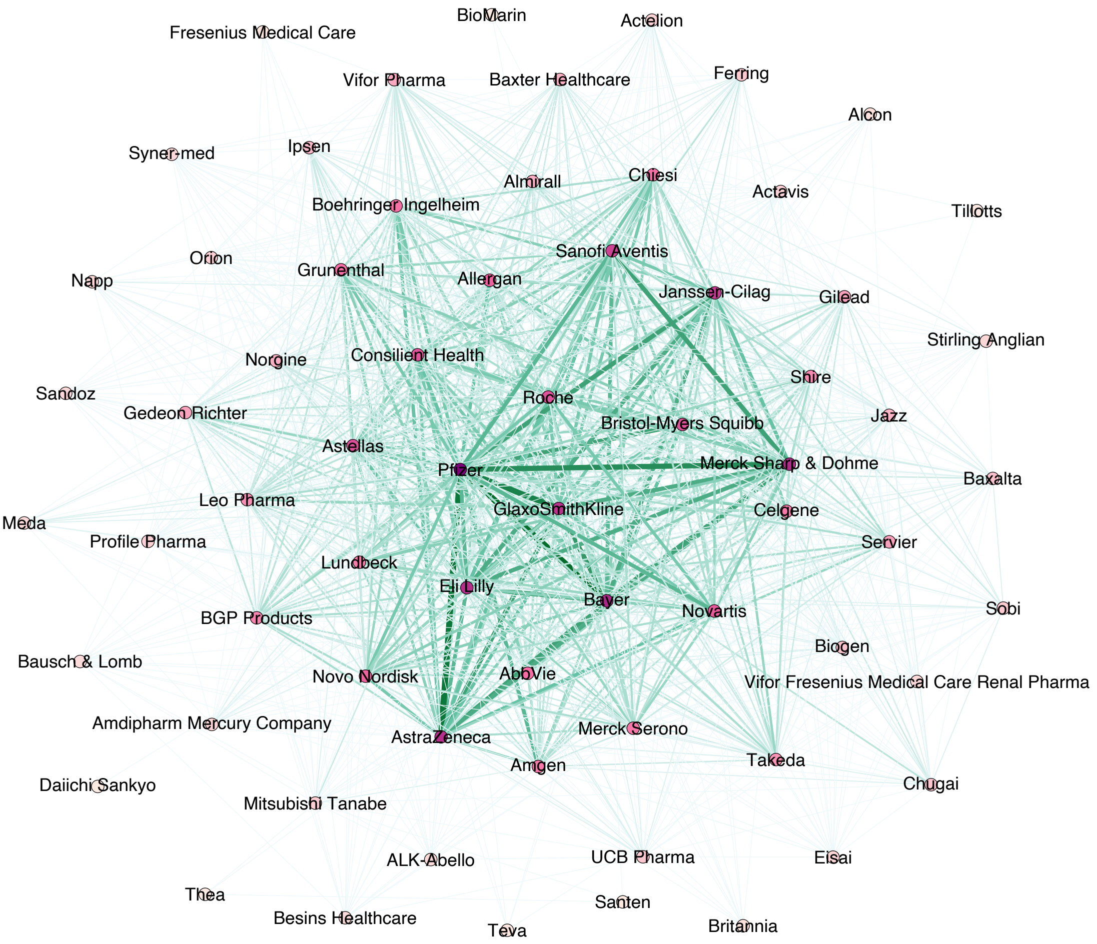

Supplementary File 5c. Wales' network

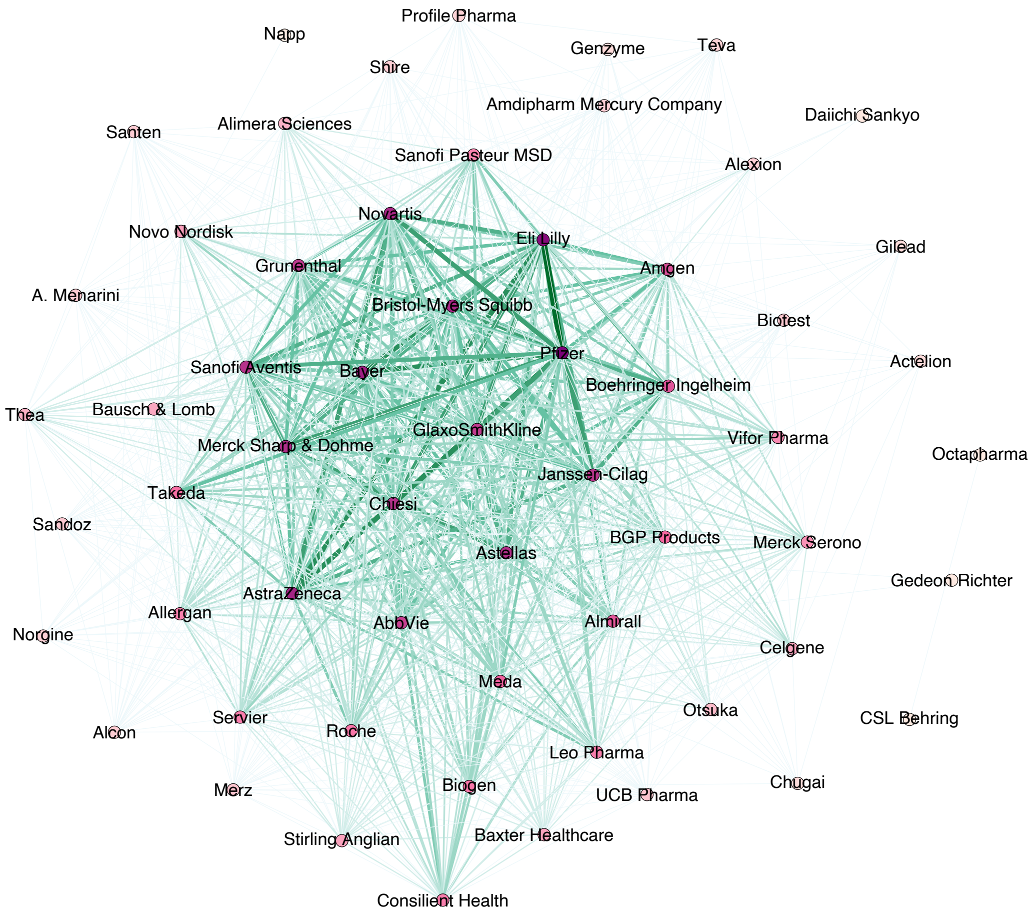

Supplementary File 5d. Northern Ireland's network

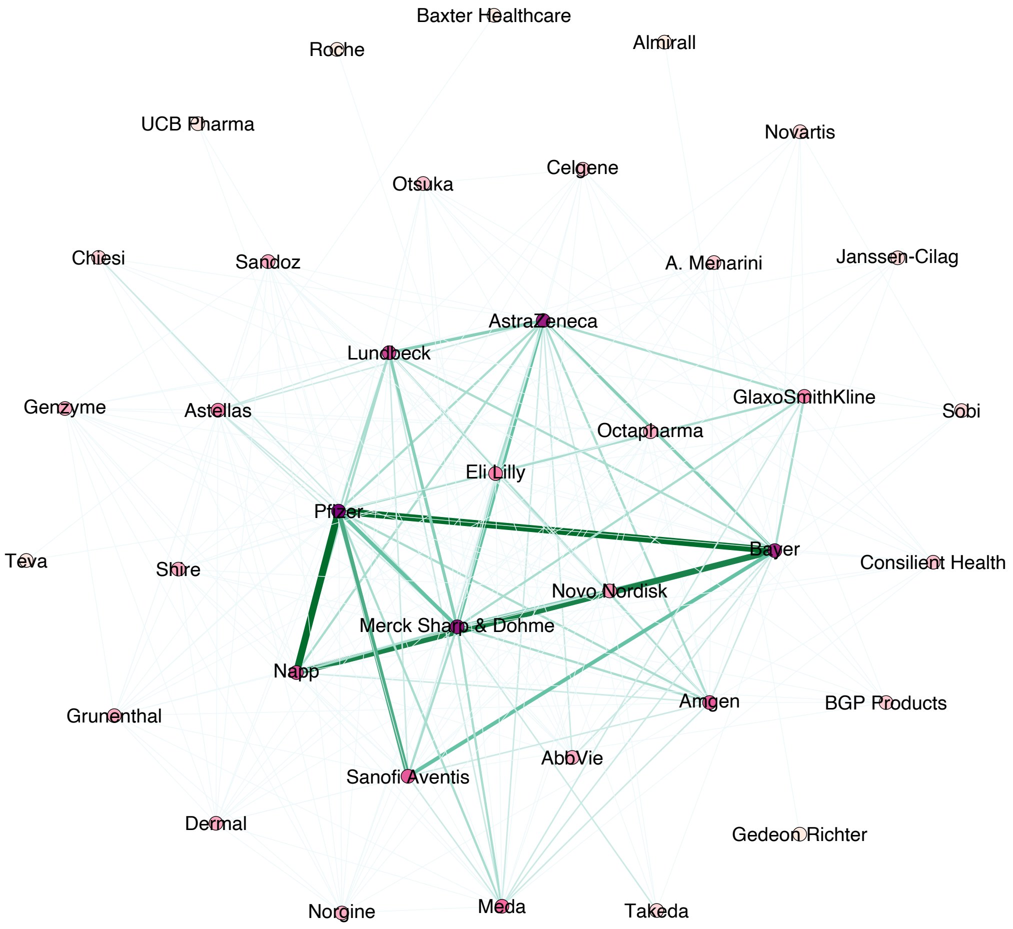

Note. All networks were visualised in Gephi v 0.9.2. Node label size and darkness corresponds to the weighted degree centrality of each company; the size and darkness of the edges (connecting lines) correspond to the number of shared recipients between companies

Supplementary File 6. Top ten companies by degree centrality scores in each country

England		Scotland		Wales		Northern Ireland	
Company	Degree	Company	Degree	Company	Degree	Company	Degree
Pfizer (2)	3394	Pfizer (2)	319	Pfizer (2)	206	Pfizer (2)	63
Merck Sharp & Dohme (6)	3064	Bayer (8)	260	Eli Lilly (3)	196	Merck Sharp & Dohme (6)	57
Bayer (8)	3060	Merck Sharp & Dohme (6)	251	Bayer (8)	176	AstraZeneca (1)	55
AstraZeneca (1)	2755	AstraZeneca (1)	245	AstraZeneca (1)	171	Bayer (8)	52
Eli Lilly (3)	2741	Eli Lilly (3)	245	Bristol-Myers Squibb (23)	169	Lundbeck (64)	43
Janssen-Cilag (10)	2539	GlaxoSmithKline (4)	241	Novartis (7)	168	Napp Pharmaceuticals (29)	39
GlaxoSmithKline (4)	2531	Janssen-Cilag (10)	227	Merck Sharp & Dohme (6)	161	Amgen (19)	36
Astellas Pharma (21)	2410	Sanofi Aventis (13)	214	Astellas Pharma (21)	160	Sanofi Aventis (13)	36
Chiesi (27)	2365	Astellas Pharma (21)	196	Chiesi (27)	158	Meda Pharma (46)	32
Sanofi Aventis (13)	2156	Bristol-Myers Squibb (23)	193	Sanofi Aventis (13)	156	Eli Lilly (3)	27

Note. Numbers in brackets represent company size (revenue ranking)

Supplementary File 7. Descriptive statistics for each recipient type

England – types of recipient	Value (%)	Payments – n (%)	Median - £ (IQR)	Recipients – n (%)	Pharmaceutical companies
Public sector secondary and tertiary care providers	**13,349,779.1 (25.56)**	**6660 (36.87)**	233.17 (141.87 – 500)	260 (7.41)	**89**
Patient organisation	12,227,843.2 (23.41)	1141 (6.32)	**4000 (500 – 11,104)**	288 (8.21)	65
Education and research providers	9,055,882.96 (17.34)	875 (4.84)	1000 (333.34 – 4,798.40)	56 (1.60)	68
Professional organisations	7,545,121.68 (14.44)	1776 (9.83)	500 (240 – 3,200)	354 (10.09)	84
Private companies other than providers of health services	3,975,461.63 (7.61)	1443 (7.99)	300 (196.8 – 598.92)	239 (6.81)	56
Public sector primary care providers	2,416,957.98 (4.63)	2513 (13.91)	434.5 (193.6 – 869)	**1809 (51.55)**	32
Private sector healthcare providers	1,322,785.04 (2.53)	463 (2.56)	240 (166 – 588)	108 (3.08)	44
Healthcare commissioning, planning and regulatory organisations	1,235,239.68 (2.36)	2166 (11.99)	208.17 (160 – 307.2)	206 (5.87)	47
Charities and other third-sector organisations	876,822.76 (1.68)	366 (2.03)	223.52 (157 – 487.2)	39 (1.11)	40
Formal bodies representing healthcare professionals or patients	121,351.93 (0.23)	458 (2.54)	200 (160 – 259.8)	68 (1.94)	25
Alternative providers of health services	93,534.91 (0.18)	180 (1.00)	200 (160 – 394)	62 (1.77)	28
Public administration and providers of public services	15,335.25 (0.03)	24 (0.13)	394.4 (224.45 – 546.67)	20 (0.57)	10
All payments	*52,445,615.48*	*18065*	*280 (160 – 827.75)*	*3509*	*100*
**Scotland - types of recipient**	**Value (%)**	**Payments - n (%)**	**Median - £ (IQR)**	**Recipients - n (%)**	**Pharmaceutical companies (%)**
Healthcare commissioning, planning and regulatory organisations	**878,333.57 (24.13)**	**582 (43.30)**	240 (131.18 - 500)	22 (8.30)	**53 (73.61)**
Private companies other than providers of health services	740,694.09 (20.35)	25 (1.86)	1,200 (350 - 5,528.88)	11 (4.15)	13 (18.06)
Education and research providers	708,149.16 (19.46)	141 (10.49)	1152 (400 – 2,880)	8 (3.02)	41 (56.94)
Patient organisation	620,384.33 (17.05)	52 (3.87)	1000 (253.68 - 9,745)	14 (5.28)	19 (26.39)
Professional organisations	466,833.11 (12.83)	291 (21.65)	450 (285.67 - 980)	64 (24.15)	52 (72.22)

Public sector primary care providers	112,308.91 (3.09)	128 (9.52)	434.5 (202.23 - 651,75)	**113 (42.64)**	13 (18.06)
Private sector healthcare providers	58,091.76 (1.60)	69 (5.13)	647.54 (206.65-1,500)	11 (4.15)	9 (12.50)
Public sector secondary and tertiary care providers	27,392.82 (0.75)	39 (2.90)	300 (189.75 - 612)	12 (4.53)	11 (15.28)
Charities and other third-sector organisations	19,710 (0.54)	4 (0.30)	**1700 (377.5 - 6,250)**	2 (0.75)	4 (5.56)
Alternative providers of health services	4,580 (0.13)	6 (0.45)	700 (360 – 1,100)	4 (1.51)	4 (5.56)
Public administration and providers of public services	2,700 (0.07)	5 (0.37)	540 (200 - 600)	3 (1.13)	5 (6.94)
Formal bodies representing healthcare professionals or patients	427.2 (0.01)	2 (0.15)	213.6 (211.4 - 214.8)	1 (0.38)	1 (1.39)
All payments	*3,649,749.43*	*1,344*	*400 (180 - 864)*	*265*	*72*
**Wales - types of recipient**	**Value (%)**	**Payments - n (%)**	**Median - £ (IQR)**	**Recipients - n (%)**	**Pharmaceutical companies (%)**
Healthcare commissioning, planning and regulatory organisations	**920,980.22 (46.38)**	**557 (56.61)**	225 (114.24 - 486.39)	10 (4.72)	**50 (78.13)**
Private companies other than providers of health services	179,495.4 (9.04)	56 (5.69)	**1475 (216 - 6,600)**	10 (4.72)	8 (12.50)
Education and research providers	179,256.38 (9.03)	37 (3.76)	336 (175.2 - 1000)	5 (2.36)	16 (25.00)
Public sector primary care providers	173,268.30 (8.73)	141 (14.33)	800 (434.5 - 1,152)	**118 (55.66)**	15 (23.44)
Private sector healthcare providers	153,983.36 (7.76)	18 (1.83)	440 (360.94 - 1,732)	6 (2.83)	9 (14.06)
Public administration and providers of public services	108,000 (5.44)	1 (0.10)	-	1 (0.47)	1 (1.56)
Patient organisation	99,784.32 (5.03)	22 (2.24)	747.93 (500 - 2,000)	10 (4.72)	11 (17.19)
Public sector secondary and tertiary care providers	96,862.66 (4.88)	20 (2.03)	253.66 (200 - 954)	3 (1.42)	13 (20.31)
Professional organisations	64,181.82 (3.23)	88 (8.94)	400 (280 - 800)	38 (17.92)	31 (48.44)
Charities and other third-sector organisations	5,036.8 (0.25)	17 (1.73)	120 (120 - 180)	4 (1.89)	7 (10.94)
Formal bodies representing healthcare professionals or patients	4,679.37 (0.24)	27 (2.74)	120 (96 - 142)	7 (3.30)	11 (17.19)
All payments	*1,987,702.62*	*984*	*300 (144 - 800)*	*212*	*64*

**Northern Ireland - types of recipient**	**Value (%)**	**Payments - n (%)**	**Median - £ (IQR)**	**Recipients - n (%)**	**Pharmaceutical companies (%)**
Public sector primary care providers	**184,903.09 (35.72)**	**127 (40.97)**	600 (434.5 - 1,600)	**94 (**60.65)	6 (14.29)
Public sector secondary and tertiary care providers	111,743.45 (21.59)	83 (26.77)	288 (163.4 - 490.13)	5 (3.23)	**27 (64.29)**
Professional organisations	81,489.7 (15.74)	34 (10.97)	600 (320 - 1,784)	21 (13.55)	21 (50.00)
Patient organisation	43,205.6 (8.35)	15 (4.84)	650 (600 - 1,450)	7 (4.52)	14 (33.33)
Education and research providers	32,258 (6.23)	10 (3.23)	1100 (873.75 - 3525)	1 (0.65)	7 (16.67)
Private companies other than providers of health services	26,242.77 (5.07)	6 (1.94)	**4179.38 (1,152.19 - 7,687.5)**	4 (2.58)	3 (7.14)
Healthcare commissioning, planning and regulatory organisations	22,447.6 (4.34)	7 (2.26)	1500 (470.8 - 4,087)	4 (2.58)	6 (14.29)
Private sector healthcare providers	11,476.85 (2.22)	23 (7.42)	38.49 (28.9 - 485)	16 (10.32)	6 (14.29)
Formal bodies representing healthcare professionals or patients	2,133.34 (0.41)	2 (0.65)	1066.67 (933.33 - 1200.00)	1 (0.65)	1 (2.38)
Alternative providers of health services	1,700 (0.33)	3 (0.97)	600 (550 - 600)	2 (1.29)	2 (4.76)
All payments	*517,600.40*	*310*	*475.2 (217.25 - 1,357.47)*	*155*	*42*

Supplementary File 8. Top 10 recipients in each country

Country	Recipient	Type of recipient	Value - £	Payments - n	Companies - n
England	King's College London	Education and research providers	2,572,086.51	45	18
	Bladder and Bowel Foundation	Patient organisation	1,459,371.52	11	1
	London School Hyg and Tropical Med	Education and research providers	935,025.98	16	6
	PeerVoice	Private companies other than providers of health services	930,028.30	11	3
	University College London	Education and research providers	907,256.40	96	36
	Diabetes UK - England	Patient organisation	888,845.00	41	7
	Healthcare At Home	Private sector healthcare providers	872,740.81	18	2
	Cancer Research UK	Patient organisation	804,543.76	19	9
	Central Manchester Univ Hosps FT	Public sector secondary and tertiary care providers	739,595.97	108	37
	British Society for Rheumatology	Professional organisations	543,012.33	31	14
Scotland	Quintiles - Scotland	Private companies other than providers of health services	682,601.65	5	1
	Myeloma UK	Patient organisation	521,574.36	12	7
	NHS Greater Glasgow and Clyde	Healthcare commissioning, planning and regulatory organisations	483,354.99	153	34
	University of Glasgow	Education and research providers	442,707.63	70	26
	University of Dundee	Education and research providers	160,632.40	20	11
	NHS Lothian	Healthcare commissioning, planning and regulatory organisations	144,175.05	73	25
	University of Edinburgh	Education and research providers	73,014.39	37	21
	NHS Tayside	Healthcare commissioning, planning and regulatory organisations	67,924.08	74	24
	NHS Ayrshire and Arran	Healthcare commissioning, planning and regulatory organisations	63,276.93	48	25
	Digestive Disorders Federation	Professional organisations	60,796.00	2	2
Wales	Cardiff and Vale University HB	Healthcare commissioning, planning and regulatory organisations	344,131.95	89	28

Rickard E, *et al. BMJ Open* 2023; 13:e061591. doi: 10.1136/bmjopen-2022-061591

	Abertawe Bro Morgannwg Univ HB	Healthcare commissioning, planning and regulatory organisations	242,418.82	124	32
	LloydsPharmacy	Private sector healthcare providers	146,376.00	4	1
	University of Cardiff	Education and research providers	120,822.78	27	13
	Bluebay Medical Systems	Private companies other than providers of health services	116,900.00	26	1
	Hywel Dda University HB	Healthcare commissioning, planning and regulatory organisations	115,600.62	77	27
	National Assembly for Wales	Public administration and providers of public services	108,000.00	1	1
	Betsi Cadwaladr University HB	Healthcare commissioning, planning and regulatory organisations	101,352.58	76	21
	Cwm Taf University Health Board	Healthcare commissioning, planning and regulatory organisations	84,624.15	119	25
	Velindre NHS Trust	Public sector secondary and tertiary care providers	80,629.32	18	13
Northern Ireland	Belfast Health and SC Trust	Public sector secondary and tertiary care providers	60,615.65	30	17
	Federation Of Family Practices	Public sector primary care providers	40,235.20	3	3
	UK and Ireland Society of Cataract and Refractive Surgeons - Northern Ireland	Professional organisations	35,000.00	1	1
	Queen's University Belfast	Education and research providers	32,258.00	10	7
	Northern Health and SC Trust	Public sector secondary and tertiary care providers	23,703.46	14	8
	Medical Communications	Private companies other than providers of health services	23,250.00	3	1
	Adult ADHD - Northern Ireland	Patient organisation	20,000.00	1	1
	Ulster Chemists' Association	Professional organisations	16,584.00	4	3
	Western Health and SC Trust	Public sector secondary and tertiary care providers	15,068.55	12	10
	Cancer Focus Northern Ireland	Patient organisation	12,255.60	2	2

Supplementary File 9. Post-hoc Bonferroni pairwise comparisons between countries of payments per recipient type

Recipient type	Group 1 – Group 2*	Test Statistic	Std. Error	Std. Test Statistic	Sig.	Adj. Sig†,‡	Median (IQR) – group 1 - £	Median (IQR) – group 2 - £
Alternative providers of health services	England-Scotland	-59.003	22.681	-2.601	0.009	**0.028**	200 (160 – 394)	700 (360 – 1,100)
	England-Northern Ireland	-65.919	31.816	-2.072	0.038	0.115	200 (160 – 394)	600 (550 - 600)
	Scotland-Northern Ireland	6.917	38.645	0.179	0.858	1	700 (360 – 1,100)	600 (550 - 600)
Charities and other third-sector organisations	England-Scotland	-118.214	56.132	-2.106	0.035	0.106	223.52 (157 – 487.2)	**1700 (377.5 - 6,250)**
	Wales-England	79.844	27.702	2.882	0.004	**0.012**	120 (120 - 180)	223.52 (157 – 487.2)
	Wales-Scotland	198.059	62.049	3.192	0.001	**0.004**	120 (120 - 180)	**1700 (377.5 - 6,250)**
Education and research providers	England-Scotland	-5.623	27.857	-0.202	0.84	1	1000 (333.34 – 4,798.40)	1152 (400 – 2,880)
	England-Northern Ireland	-92.61	97.628	-0.949	0.343	1	1000 (333.34 – 4,798.40)	1100 (873.75 - 3525)
	Scotland-Northern Ireland	86.987	100.458	0.866	0.387	1	1152 (400 – 2,880)	1100 (873.75 - 3525)
	Wales-England	204.193	51.523	3.963	<.001	**0**	336 (175.2 - 1000)	1000 (333.34 – 4,798.40)
	Wales-Scotland	209.816	56.703	3.7	<.001	**0.001**	336 (175.2 - 1000)	1152 (400 – 2,880)
	Wales-Northern Ireland	296.803	109.409	2.713	0.007	**0.04**	336 (175.2 - 1000)	1100 (873.75 - 3525)
Formal bodies representing healthcare professionals	England-Scotland	-46.978	99.854	-0.47	0.638	1	200 (160 – 259.8)	213.6 (211.4 - 214.8)
	England-Northern Ireland	-223.228	99.854	-2.236	0.025	0.152	200 (160 – 259.8)	1066.67 (933.33 - 1200.00)
	Scotland-Northern Ireland	176.25	140.907	1.251	0.211	1	213.6 (211.4 - 214.8)	1066.67 (933.33 - 1200.00)
	Wales-England	138.133	27.905	4.95	<.001	**0**	120 (96 - 142)	200 (160 – 259.8)
	Wales-Scotland	185.111	103.261	1.793	0.073	0.438	120 (96 - 142)	213.6 (211.4 - 214.8)

	Wales-Northern Ireland	361.361	103.261	3.5	<.001	**0.003**	120 (96 - 142)	1066.67 (933.33 - 1200.00)
Healthcare commissioning, planning and regulatory organisations	England-Scotland	-122.159	44.623	-2.738	0.006	**0.037**	208.17 (160 – 307.2)	240 (131.18 - 500)
	England-Northern Ireland	-786.684	361.817	-2.174	0.03	0.178	208.17 (160 – 307.2)	1500 (470.8 - 4,087)
	Scotland-Northern Ireland	664.525	363.4	1.829	0.067	0.405	240 (131.18 - 500)	1500 (470.8 - 4,087)
	Wales-Scotland	94.65	56.651	1.671	0.095	0.569	225 (114.24 - 486.39)	240 (131.18 - 500)
	Wales-Northern Ireland	759.175	363.497	2.089	0.037	0.22	225 (114.24 - 486.39)	1500 (470.8 - 4,087)
	England-Wales	-27.51	45.405	-0.606	0.545	1	208.17 (160 – 307.2)	225 (114.24 - 486.39)
Patient organisation	Wales-England	144.727	77.321	1.872	0.061	0.367	747.93 (500 - 2,000)	**4000 (500 – 11,104)**
	Wales-Scotland	29.422	91.028	0.323	0.747	1	747.93 (500 - 2,000)	1000 (253.68 - 9,745)
	Northern Ireland-Wales	-8.633	118.686	-0.073	0.942	1	650 (600 - 1,450)	747.93 (500 - 2,000)
	Northern Ireland-Scotland	-38.055	103.121	-0.369	0.712	1	650 (600 - 1,450)	1000 (253.68 - 9,745)
	Northern Ireland-England	153.361	91.248	1.681	0.093	0.557	650 (600 - 1,450)	**4000 (500 – 11,104)**
	Scotland-England	115.306	50.259	2.294	0.022	0.131	1000 (253.68 - 9,745)	**4000 (500 – 11,104)**
Private companies other than providers of health services	England-Scotland	-317.041	89.112	-3.558	<.001	**0.002**	300 (196.8 – 598.92)	1,200 (350 - 5,528.88)
	England-Northern Ireland	-527.308	180.717	-2.918	0.004	**0.021**	300 (196.8 – 598.92)	**4179.38 (1,152.19 - 7,687.5)**
	Scotland-Northern Ireland	210.267	200.821	1.047	0.295	1	1,200 (350 - 5,528.88)	**4179.38 (1,152.19 - 7,687.5)**
	Wales-Scotland	115.061	106.256	1.083	0.279	1	**1475 (216 - 6,600)**	1,200 (350 - 5,528.88)
	Wales-Northern Ireland	325.327	189.758	1.714	0.086	0.519	**1475 (216 - 6,600)**	**4179.38 (1,152.19 - 7,687.5)**

	England-Wales	-201.98	60.166	-3.357	<.001	**0.005**	300 (196.8 – 598.92)	**1475 (216 - 6,600)**
Private sector healthcare providers	England-Scotland	-64.982	21.355	-3.043	0.002	**0.014**	240 (166 – 588)	647.54 (206.65 - 1,500)
	England-Wales	-88.107	39.755	-2.216	0.027	0.16	240 (166 – 588)	440 (360.94 - 1,732)
	Northern Ireland-Wales	-206.778	52.076	-3.971	<.001	**0**	38.49 (28.9 - 485)	440 (360.94 - 1,732)
	Northern Ireland-Scotland	-183.652	39.843	-4.609	<.001	**0**	38.49 (28.9 - 485)	647.54 (206.65 - 1,500)
	Northern Ireland-England	118.671	35.352	3.357	<.001	**0.005**	38.49 (28.9 - 485)	240 (166 – 588)
	Scotland-wales	-23.126	43.797	-0.528	0.597	1	647.54 (206.65 - 1,500)	440 (360.94 - 1,732)
Professional organisations	England-Northern Ireland	-35.169	109.4	-0.321	0.748	1	500 (240 – 3,200)	600 (320 - 1,784)
	Scotland-Northern Ireland	157.615	114.524	1.376	0.169	1	450 (285.67 - 980)	600 (320 - 1,784)
	Wales-England	182.447	69.008	2.644	0.008	**0.049**	400 (280 - 800)	500 (240 – 3,200)
	Wales-Scotland	60.001	76.873	0.781	0.435	1	400 (280 - 800)	450 (285.67 - 980)
	Wales-Northern Ireland	217.616	127.597	1.705	0.088	0.529	400 (280 - 800)	600 (320 - 1,784)
	Scotland-England	122.446	39.962	3.064	0.002	**0.013**	450 (285.67 - 980)	500 (240 – 3,200)
Public sector primary care providers	England-Northern Ireland	-355.779	76.292	-4.663	<.001	**0**	434.5 (193.6 – 869)	600 (434.5 - 1,600)
	Scotland-Northern Ireland	407.76	105.06	3.881	<.001	**0.001**	434.5 (202.23 - 651,75)	600 (434.5 - 1,600)
	England-Wales	-459.79	72.597	-6.333	<.001	**0**	434.5 (193.6 – 869)	800 (434.5 - 1,152)
	Northern Ireland-Wales	-104.011	102.62	-1.014	0.311	1	600 (434.5 - 1,600)	800 (434.5 - 1,152)
	Scotland-England	51.98	76.008	0.684	0.494	1	434.5 (202.23 - 651,75)	434.5 (193.6 – 869)
	Scotland-wales	-511.771	102.409	-4.997	<.001	**0**	434.5 (202.23 - 651,75)	800 (434.5 - 1,152)

*Each row tests the null hypothesis that the Sample 1 and Sample 2 distributions are the same.
†Asymptotic significances (2-sided tests) are displayed. The significance level is .050.
‡Significance values have been adjusted by the Bonferroni correction for multiple tests

Supplementary File 10. Post-hoc Bonferroni pairwise comparisons of payment types between countries

Payment Type*	Group 1 – Group 2**	Test Statistic	Std. Error	Std. Test Statistic	Sig.	Adj. Sig†,‡	Median (IQR) – group 1 - £	Median (IQR) – group 2 - £
Contribution to costs of Events	Wales-England	498.254	159.743	3.119	0.002	0.011	223.36 (120 - 400)	240 (155.95 - 400)
	Wales-Scotland	1449.82	208.45	6.955	<.001	0	223.36 (120 - 400)	300 (160 - 600)
	Wales-Northern	2690.485	402.839	6.679	<.001	0	223.36 (120 - 400)	477.6 (200 - 1,147.6)
	England-Scotland	-951.566	143.605	-6.626	<.001	0	240 (155.95 - 400)	300 (160 - 600)
	England-Northern	-2192.23	373.43	-5.871	<.001	0	240 (155.95 - 400)	477.6 (200 - 1,147.6)
	Scotland-Northern	1240.664	396.716	3.127	0.002	0.011	300 (160 - 600)	477.6 (200 - 1,147.6)
Donations and Grants	Wales-England	25.151	94.77	0.265	0.791	1	800 (434.5 - 2,200)	959.98 (256 - 4,800)
	Northern-Scotland	-140.935	137.745	-1.023	0.306	1	434.5 (217.5 - 1,867.5)	651.75 (217.25 - 2,578)
	Northern-Wales	-451.347	142.863	-3.159	0.002	0.009	434.5 (217.5 - 1,867.5)	800 (434.5 - 2,200)
	Northern-England	476.498	111.315	4.281	<.001	0	434.5 (217.5 - 1,867.5)	959.98 (256 - 4,800)
	Scotland-Wales	-310.412	124.755	-2.488	0.013	0.077	651.75 (217.25 - 2,578)	800 (434.5 - 2,200)
	Scotland-England	335.563	86.862	3.863	<.001	0.001	651.75 (217.25 - 2,578)	959.98 (256 - 4,800)

*Kruskal-Wallis results: Contributions to cost of events $\chi2(3) = 89.680$, p = .000; Donations and grants $\chi2(3) = 31.698$, p = <.000

**Each row tests the null hypothesis that the Sample 1 and Sample 2 distributions are the same.

†Asymptotic significances (2-sided tests) are displayed. The significance level is .050.

‡Significance values have been adjusted by the Bonferroni correction for multiple tests.

Rickard E, et al. BMJ Open 2023; 13:e061591. doi: 10.1136/bmjopen-2022-061591
