# Supplementary material for: Comparing pharmaceutical company payments in the four UK countries: a cross-sectional and social network analysis
Source: BMJ Open. 2023 Mar 28;13(3):e061591. doi: 10.1136/bmjopen-2022-061591 (PMC10069501; doi:10.1136/bmjopen-2022-061591)
Supplement: Author's manuscript [file bmjopen-2022-061591.draft_revisions.pdf]

BMJ Open

BMJ Open is committed to open peer review. As part of this commitment we make the peer review history of every article we publish publicly available.

When an article is published we post the peer reviewers' comments and the authors' responses online. We also post the versions of the paper that were used during peer review. These are the versions that the peer review comments apply to.

The versions of the paper that follow are the versions that were submitted during the peer review process. They are not the versions of record or the final published versions. They should not be cited or distributed as the published version of this manuscript.

BMJ Open is an open access journal and the full, final, typeset and author-corrected version of record of the manuscript is available on our site with no access controls, subscription charges or pay-per-view fees (http://bmjopen.bmj.com).

If you have any questions on BMJ Open's open peer review process please email info.bmjopen@bmj.com

BMJ Open

Comparing drug company payments in the four UK countries: a cross-sectional and social network analysis

Journal:	*BMJ Open*
Manuscript ID	bmjopen-2022-061591
Article Type:	Original research
Date Submitted by the Author:	31-Jan-2022
Complete List of Authors:	Rickard, Emily; University of Bath, Social and Policy Sciences<br>Ozieranski, Piotr; University of Bath, Department of Social and Policy Sciences
Keywords:	Health policy < HEALTH SERVICES ADMINISTRATION & MANAGEMENT, MEDICAL ETHICS, PUBLIC HEALTH

SCHOLARONE™
Manuscripts

I, the Submitting Author has the right to grant and does grant on behalf of all authors of the Work (as defined in the below author licence), an exclusive licence and/or a non-exclusive licence for contributions from authors who are: i) UK Crown employees; ii) where BMJ has agreed a CC-BY licence shall apply, and/or iii) in accordance with the terms applicable for US Federal Government officers or employees acting as part of their official duties; on a worldwide, perpetual, irrevocable, royalty-free basis to BMJ Publishing Group Ltd ("BMJ") its licensees and where the relevant Journal is co-owned by BMJ to the co-owners of the Journal, to publish the Work in this journal and any other BMJ products and to exploit all rights, as set out in our licence.

The Submitting Author accepts and understands that any supply made under these terms is made by BMJ to the Submitting Author unless you are acting as an employee on behalf of your employer or a postgraduate student of an affiliated institution which is paying any applicable article publishing charge ("APC") for Open Access articles. Where the Submitting Author wishes to make the Work available on an Open Access basis (and intends to pay the relevant APC), the terms of reuse of such Open Access shall be governed by a Creative Commons licence – details of these licences and which Creative Commons licence will apply to this Work are set out in our licence referred to above.

Other than as permitted in any relevant BMJ Author's Self Archiving Policies, I confirm this Work has not been accepted for publication elsewhere, is not being considered for publication elsewhere and does not duplicate material already published. I confirm all authors consent to publication of this Work and authorise the granting of this licence.

For peer review only

Comparing drug company payments in the four UK countries: a cross-sectional and social network analysis

Emily Rickard
Department of Social and Policy Sciences, University of Bath, Bath, United Kingdom.
Email: e.j.rickard@bath.ac.uk

Piotr Ozieranski (Corresponding Author)
Department of Social and Policy Sciences, University of Bath, Bath, United Kingdom.
Email: p.ozieranski@bath.ac.uk

Word count: 4447

Key words
Transparency, financial disclosures, pharmaceutical industry, health policy, conflicts of interest

Acknowledgements
We would like to express our gratitude to Dr Emma Carmel for reading and commenting on the manuscript.

ABSTRACT

Objectives To examine general patterns in drug company payments to healthcare and patient organisations in the four UK countries, including payment and recipient prioritisation.

Design Cross-sectional descriptive and social network analysis.

Setting England, Scotland, Wales, Northern Ireland.

Participants 100 donors (drug companies) reporting payments to 4,229 recipients (healthcare organisations and patient organisations) in 2015.

Main outcome measures Overlap of payments in Disclosure UK and disclosures of payments to patient organisations. For each country: payment totals and distribution; average number of common recipients between companies; share of payments to different recipient categories and payment types.

Results Payments to patient organisations incorrectly reported as payments to healthcare organisations were identified, and some were duplicated in both datasets. Consistent with the population size in each country, England received the most payments (£52.4m), followed by Scotland (£3.65m), Wales (£1.99m) and Northern Ireland (£518,000). Significant differences existed in the distribution of payments across the four countries. Recipients in

For peer review only

England and Wales received smaller individual payments (medians of £280 and £300) and Scotland and Northern Ireland received larger individual payments (£400 and £475.2). Drug companies prioritised different recipient categories in each country: public sector secondary and tertiary care providers in England; healthcare commissioning, planning and regulatory organisations in Scotland and Wales; and public sector primary care providers in Northern Ireland. The type of payments prioritised also differed by country.

Conclusions

Disclosure policies may benefit from being segmented to better apply to the specific context and issues raised in each UK country. We call for a database containing all industry payments, including payments to healthcare and patient organisations, in one place, and disclosed with recipients' full location details. National level data and regulation may obscure important regional payment variations and recipient vulnerabilities.

STRENGTHS AND LIMITATIONS OF THIS STUDY

- The first study to comparatively assess pharmaceutical industry payments across the UK countries
- We compiled payments disclosed in the Disclosure UK database and on drug company websites to create a database of 20,861 payments made by 100 companies
- We use social network analysis to facilitate a systematic sub-national comparison of payments
- Our study does not capture the relative share of recipients' income represented by industry payments
- The study draws on one year's worth of payments, however longer-term patterns in funding may be what makes a bigger difference in shaping policy and health practice

For peer review only

INTRODUCTION

The pharmaceutical industry's 'web of influence', whereby companies "sustain large networks to gather, create, control and disseminate information"[1], and potentially distort public health research and policy to favour commercial interests above patients, has been subjected to increased scrutiny[2 3]. From the perspective of patients, it is integral that this web is understood as it presents opportunities for industry to directly influence those they are funding and indirectly influence governmental organisations which are often unaware that organisations may be, even inadvertently, promoting the interests of powerful industry actors[3].

In the United States, pharmaceutical industry disclosures of payments to physicians and teaching hospitals were made mandatory in 2013[4], and subsequent research has examined the risk of individual-level conflicts of interest (COIs) through payments to physicians[5-16], with institutional COIs through payments to hospitals largely ignored[17]. Further, payments to patient organisations, which provide support and advocate for people with specific conditions[18], have been seldom explored in the US[19 20] as their disclosure is not regulated by the state or industry. However, the potential consequences of institutional COIs can be as damaging as individual[21-23], leading to increased prescriptions of drugs with unproven safety[21], distorting research agendas[24], and compromising research integrity[25].

The situation regarding payments to patient organisations in Europe is very different due to the prevalence of industry self-regulation[26 27]. In 2012, the European trade association, European Federation of Pharmaceutical Industries and Associations (EFPIA), mandated drug companies disclose payments to patient organisations annually[28]. Subsequent studies revealed extensive industry-patient organisations ties in the UK[29 30] and the Nordic countries[31 32]. Separately, disclosures of payments to healthcare professionals and healthcare organisations, such as care providers and professional associations, were mandated in 2014 as part of self-regulatory arrangements[33]. Most research attention has been on the poor accessibility and quality of the data[26], noting lack of standardisation and categorisation of recipients[11 34] and insufficient details about the purpose of individual payments[34].

Despite healthcare and patient organisations being part of similar self-regulatory disclosure arrangements in Europe, our study is the first to our knowledge to consider them jointly because they are subject to different requirements within self-regulation – healthcare organisations are in a centralised database, Disclosure UK, and patient organisations are on company websites. These organisations have shared interests including providing patient care and support[17 29], involvement in policy-making[2 35 36], and conducting clinical research[23 29]. Pharmaceutical companies make payments to both, however solely analysing them separately may underestimate industry's web of influence and potential COIs in particular domains of healthcare and reinforcement effects of payments to organisations with shared goals, interests or personnel[2].

Another aspect of the industry's web of influence largely unexplored in Europe is targeting payments regionally, which may be characterised by strategic targeting of particular fields of healthcare provision and decision-making and lead to COIs in regional policy-making. Regionally targeted payments can also have direct effects on commissioning, operational

For peer review only - http://bmjopen.bmj.com/site/about/guidelines.xhtml

funding, and organisational priorities, and bear greater influence on day-to-day practices in particular healthcare fields. Emerging US research examines payments to physicians regionally, finding payment distributions differ significantly between states[6 37-39]. There are also payment disparities between states of different sizes[40] and political leaning[41], indicating that demographics and the organisation and regulation of healthcare and lobbying matter.

The first regional analysis in Europe revealed differences in the total value and type of payments prioritised in eight countries[42]. Most recently a UK found headquarter distance from country capitals to be a significant predictor of patient organisations' dependence on drug companies funding[43]. The primary reason research has not considered locations of UK healthcare organisation payment recipients is that Disclosure UK does not report which UK country they are in[11 14 42].

The UK presents a crucial case for sub-national analysis given its importance for the pharmaceutical industry as a market[44], the large value of payments compared to other European countries[42] and its vast charitable sector comprising many potential recipients[45]. Moreover, there are four separate health systems in England, Scotland, Wales and Northern Ireland as power over health was devolved in 1998, giving the four countries increased autonomy to shape their health policies and services[46-48]. Demographic differences include population sizes - England's population size is biggest, followed by Scotland, Wales, and Northern Ireland[49]; in per-person spending on healthcare - highest in Northern Ireland and lowest in England; health outcomes – highest in England and lowest in Scotland[50]; and number of prescriptions per capita – highest in Wales and lowest in Scotland[51]. These distinctions are overlooked in national UK analysis, potentially leading to results reflective of England only, as the largest country[31] and obscuring country-specific patterns. Further, pharmaceutical companies often spend more on drug promotion than drug development[52], making marketing decisions around *where* to spend money key[53 54]. Studies have begun acknowledging the country distinction, for example when analysing payments to Clinical Commissioning Groups[55] and GP surgeries[56] in England. We know from insights at the UK-level that drug companies prioritise different types of healthcare[11 14] and patient[29] organisations, and make different types of payments, however the extent that this translates to the devolved countries of the UK is unclear.

Our objective is to examine general patterns in drug company payments to healthcare and patient organisations in the four UK countries, including payment and recipient prioritisation.

METHODS

Data sources

Our primary data sources are publicly available pharmaceutical industry transparency disclosures from 2015. Corresponding to relevant Association for the British Pharmaceutical Industry (ABPI)[57] and EFPIA Codes[28], drug companies disclose payments to healthcare organisations and to patient organisations separately and in different ways.

Payments to healthcare organisations are disclosed in a single, centralised database of payments, Disclosure UK, published annually by the ABPI. Payments are disclosed with:

For peer review only - http://bmjopen.bmj.com/site/about/guidelines.xhtml

recipient name, payment type (donations and grants, costs of events, joint working, and consultancy – see Supplementary File 1), some address details, and payment value. We use the 2015 version of Disclosure UK and focus on non-R&D payments to healthcare organisations. (R&D payments are reported as one lump sum without named recipients[11 33]). We standardised all recipient names and categorised them based on their primary purpose (see Supplementary File 2 for a detailed breakdown). As Disclosure UK provides incomplete addresses, we conducted independent web searches to determine recipient countries. We detail data preparation, including the approach to data cleaning and coding, elsewhere[11].

Payments to patient organisations are available on drug company websites and are usually presented as a PDF file and include recipient name, payment description, and payment value. We extracted the payments to patient organisation data into a single database, standardising names and identifying headquarter addresses as part of another project[29].

Dataset integration

We merged the Disclosure UK and patient organisation datasets by firstly searching all charities and third-sector organisations disclosed as recipients in Disclosure UK to identify and recategorise patient organisations (therefore, we have two distinctive categories – charities and third-sector organisations; and patient organisations). Secondly, we standardised names of patient organisations appearing in both datasets to ensure these were not considered separately. Thirdly, we recoded the patient organisation descriptions to match payment types used in Disclosure UK (see Supplementary File 1).

Analysis

We used a combination of descriptive and social network analysis (SNA). We calculated the total and median value of payments in each country and recipient category. The Shapiro-Wilks test of normalcy found the data to be non-parametric in each country, therefore non-parametric Kruskall-Wallis tests (adjusted for ties) were used to check between-country differences in the distribution of payments. Dunn's post-hoc pairwise analyses (with Bonferroni's correction for multiple comparisons) were conducted to identify differences between countries. Statistical significance was set at $p = \leq .05$.

SNA was used to calculate connections between drug companies via a shared interest in recipients, measured by the number of common payment recipients between pairs of drug companies (density) and all drug companies (degree centrality). See Supplementary File 3 for more detailed description of SNA measures. We examine the number of common recipients in each country and recipient category and compare the network values. We created matrices of drug companies with ties based on the number of shared recipients ('valued matrices') in each country and category. Each network consisted only of the companies making at least one payment to take into account the 'nested structure' of the data[58] to ensure network statistics were not negatively skewed by companies making no payments.

Data was processed in Microsoft Excel. We analysed the data descriptively in SPSS version 27 (IBM) and Microsoft Excel. We conducted social network analysis in UCINET version 6[59]. Country networks were visualised in Gephi version 0.9.2.

For peer review only - http://bmjopen.bmj.com/site/about/guidelines.xhtml

For peer review only

Outcome measures

First, we assessed the level of overlap between the Disclosure UK and patient organisation databases by measuring the number of payments to patient organisations disclosed as healthcare organisations in Disclosure UK and the number of payments reported in both databases.

Second, we explored the payment characteristics in each country. We measured total and median value and the number of: payments, recipients, and companies. We adjusted the total value by population size for comparison. We also compared the distribution of payments between each country using Kruskall-Wallis tests.

Third, we observed the top 10% of companies making payments in each country to compare payment strategies.

Fourth, we assessed the extent to which companies make payments to the same recipients by measuring the average number of common recipients between each pair of companies (degree centrality).

Fifth, we scrutinised which companies dominate the payment networks in each country by identifying the number of recipients that each company had in common with every other company.

Sixth, for each country we examined the most prioritised recipient categories as measured by the proportion of payments received by recipients in each category. to compare the weighted importance of each recipient category. We also compared the distribution of payments in each recipient category using Kruskall-Wallis tests to determine whether payments to similar types of recipients differ between countries.

Seventh, we examined whether companies making payments in *each category* in each country made payments to the same recipients by measuring the average number of recipients each pair of companies share.

Finally, for each country we assessed which types of payments were prioritised through identifying the proportion of different payment types. We also compared the four types of payments using Kruskall-Wallis tests to identify differences in the distribution of payments.

Patient and public involvement
The study did not involve patients.

RESULTS

Duplicate payments
When integrating the Disclosure UK and patient organisation data (see Supplementary File 4 for data integration flowchart), we identified 341 payments (1.71% of all payments in Disclosure UK) to 116 patient organisations (2.88% of all organisations) worth £2,458,931.99 (5.21% of the total) incorrectly disclosed as healthcare organisations in Disclosure UK. We

For peer review only - http://bmjopen.bmj.com/site/about/guidelines.xhtml

also found 50 payments duplicated in the patient organisation and Disclosure UK data, which were excluded to ensure no payment was counted twice.

Payment characteristics in each UK country

The total value and number of payments, the number of recipients, and the number of companies making payments were consistent with the size of each country, with England receiving the highest and Northern Ireland the lowest (this was maintained after adjusting for population size – see Table 1).

Table 1. Value and number of payments, number of companies and recipients, and top donors in integrated dataset

Descriptive statistic	England	Scotland	Wales	Northern Ireland
Country population 2015* - n	54.8m	5.4m	3.1m	1.9m
Total value - £	52,445,615.48	3,649,749.43	1,987,702.62	518,000.40
Total value - £ (adjusted for population size)†	957,036.78	675,879.52	641,194.39	272,631.79
Payments - n	18,190	1,370	990	311
Recipients - n	3,575	282	216	156
Companies – n	100	72	64	42
Median payment value (IQR) - £	280 (665.53)	400 (685.25)	300 (658.20)	475.20 (1,164.35)
Value of payments to healthcare organisations - £	40,217,772.30	3,029,365.10	1,887,918.30	474,794.80
Value of payments to patient organisations - £	12,227,843.18	620,384.33	99,784.32	43,205.6

*Data obtained from the Office for National Statistics, values correct for mid-2015
†Total value of payments divided by the population size

Between-country differences in payment values

Smaller countries generally received larger individual payments - higher medians were observed in Scotland, Wales and Northern Ireland compared to the largest country England (Table 1). A Kruskall-Wallis Test revealed a statistically significant difference in the distribution of individual payments between the four countries, $\chi 2(3) = 50.127$, p = <.001. Post-hoc Bonferroni pairwise comparisons showed that this difference was significant between Wales-NI (p = <.000), England-Scotland (p = <.001), England-NI (p = <.001), Wales-Scot (p = .001), and Scotland-NI (p = .004).

Top donors in each country

Focusing on the top 10% of donors in each country, we can identify different approaches to payments across the four countries (see Supplementary File 5). Top donors generally made larger payments in Wales and multiple smaller payments in Northern Ireland. Pfizer was consistently a top donor with a high volume of payments in all four countries. In England, Novartis was the second biggest donor characterised by large payments; similar patterns characterised Biogen's payments in Scotland and Wales. The top donors in each country

For peer review only - http://bmjopen.bmj.com/site/about/guidelines.xhtml

were generally consistent, although England, Scotland and Northern Ireland all had at least one company not featuring as top donors in another country.

Within-country connections between companies

The average value density scores indicate that companies were most strongly connected via a shared interest in recipients in England (on average, companies had between six and seven recipients in common). Companies, on average, had at least one recipient in common with another company in Scotland and Wales, and were least connected in Northern Ireland (Table 2). The average weighted degree density score shows the average number of recipients a company shares with *all* companies in the network, where similarly the highest score was observed in England (664.36 recipients) and lowest in Northern Ireland. The visualised networks are in Supplementary File 6.

Table 2. Drug company connections in each country measured by common recipients

Network measure	England	Scotland	Wales	Northern Ireland
Density – average value (average number of ties* between two companies)	6.71	1.24	1.13	0.42
Density – average weighted degree† (average number of ties for all companies in the network)	664.36	88.39	71.06	17.38
Company with highest degree centrality score	Pfizer (3,394)	Pfizer (319)	Pfizer (206)	Pfizer (63)

*Ties are shared recipients

Note. Calculations were conducted on valued networks which means they consider the number of common recipients. Networks include only companies making payments in each country.

At the company level, the degree centrality scores indicated that Pfizer was the most connected company in each country indicated by expressing a shared interest in recipients with other companies (Table 2). The top ten most connected companies (see Supplementary File 7) were similar in England, Scotland and Wales and featured many of the biggest companies measured by average UK turnover, including AstraZeneca and Pfizer (ranked 1st and 2nd respectively) as well as lower revenue companies including Astellas (ranked 21st). Northern Ireland's top ten most connected companies differed more, and also featured AstraZeneca, as well as smaller companies such as Meda and Lundbeck, suggesting that a cluster of companies had a particular interest in pockets of Northern Ireland's health system. Further, the top ten most connected companies were not always top donors, for example highly connected companies Chiesi in England and Novartis in Wales were not top donors in these countries.

Most prioritised recipient categories in each country

The share of the total value of payments received by recipient categories in each country revealed distinctive patterns (Figure 1). In Wales, healthcare commissioning, planning and regulatory organisations received just under half of all payments - £920,980.22 (46.38% of Wales' total payments, see Supplementary File 8 for values). The top recipient in this category was Cardiff and Vale University Health Board (see Supplementary File 9 for top recipients). The same category was prioritised in Scotland (£878,333.57 – 24.13%); NHS Greater Glasgow and Clyde was the top recipient. Scotland's private companies other than

For peer review only - http://bmjopen.bmj.com/site/about/guidelines.xhtml

providers of health services were also prioritised, data analytics firm Quintiles in particular. In England, public sector secondary and tertiary care providers received the most funding (£13,349,779.1 – 25.56%), with Central Manchester University Hospitals Foundation Trust the top recipient, closely followed by patient organisations (23.41%). In Northern Ireland, public sector primary care providers were targeted with the most funding (£184,903.09 – 35.72%); Belfast Health and SC Trust was the top recipient.

Figure 1. Percentage of payments to recipients in each category per country

Education and research providers, namely universities, in England and Scotland, and professional organisations in England, Scotland and Northern Ireland also received large payment shares.

A Kruskall-Wallis Test revealed statistically significant differences in the distribution of payments between countries for ten of the twelve categories of recipients (see Table 3), indicating that payment sizes vary between countries even when the recipient category is the same.

Post-hoc Bonferroni pairwise analyses maintained the significant differences, except for in patient organisations (see Supplementary File 10). Differences in the distribution of payments were frequent in private sector healthcare providers, with Wales, Scotland and England all having significantly higher payment values than Northern Ireland, and Scotland higher than England. In public sector primary care providers, Northern Ireland and Wales both received higher value payments than England and Scotland. In education and research providers, England's, Scotland's, and Northern Ireland's values were significantly higher than Wales.

Table 3. Independent-samples Kruskall-Wallis H Test – differences between countries

Recipient category	Recipients – n	Test statistic (H)**	Degrees of freedom	P value
Alternative providers of health services	189	10.818	2	0.004*
*Charities and other third-sector organisations****	387	13.015	2	0.001*
Education and research providers	1063	16.988	3	0.001*
Formal bodies representing healthcare professionals	489	30.142	3	<.000*
Healthcare commissioning, planning and regulatory organisations	3312	11.919	3	0.008*
Patient organisations	1216	11.092	3	0.011*
Private companies other than providers of health services	1530	31.453	3	<.000*
Private sector healthcare providers	573	26.859	3	<.000*
Professional organisations	2189	15.452	3	0.001*
Public administration and providers of public services	30	2.872	2	0.238
Public sector primary care providers	2909	60.482	3	<.000*
Public sector secondary and tertiary care providers	6802	7.616	3	0.055

For peer review only - http://bmjopen.bmj.com/site/about/guidelines.xhtml

*Statistically significant
**All test statistics are adjusted for ties in SPSS
***excluding providers of health services and professional organisations and not patient organisations

Extent of connections between companies in each recipient category

Overall, companies targeting common recipients was highest in England's public sector secondary and tertiary care providers (5.8 common recipients on average – Table 4), evidencing industry's shared interest in key recipients in this category.

In Scotland and Wales, companies had common recipients most frequently in healthcare commissioning, planning and regulatory organisations. In Northern Ireland, compared to the country's overall density score, the score for public sector primary care providers category was very high, suggesting that companies have a particular shared interest in Northern Ireland's primary care system.

Density scores were generally higher in categories receiving a high share of payments, however there were exceptions characterised by relatively low density scores. Notable cases include professional organisations and patient organisations, indicating that although these categories benefitted financially, companies had fewer common interests in particular recipients.

Table 4. Density scores for valued recipient category networks in each country

Recipient category	England	Scotland	Wales	Northern Ireland
Alternative providers of health services	0.339	0.500	-	0.000
Charities and other third-sector organisations	0.510	0.333	0.476	-
Education and research providers	1.194	0.727	0.675	1.000
Formal bodies representing healthcare professionals	1.293	0.000	0.400	0.000
Healthcare commissioning, planning and regulatory organisations	2.523	1.578	1.634	0.133
Patient organisations	0.337	0.200	0.109	0.209
Private companies other than providers of health services	0.312	0.121	0.071	0.000
Private sector healthcare providers	0.416	0.167	0.167	0.067
Professional organisations	0.611	0.244	0.114	0.038
Public administration and providers of public services	0.022	0.300	0.000	-
Public sector primary care providers	0.893	0.038	0.124	1.600
Public sector secondary and tertiary care providers	5.819	1.309	1.000	0.826

Note. Density scores measure the average tie strength (average number of common recipients between two companies). The network matrix for each category consisted only of companies making payments. Dashes indicate no payments were made. Scores of 0.000 indicate all recipients received payments from one company only.

For peer review only

Prioritised payment types in each country
The types of payments drug companies made also varied between countries (Figure 2). Donations and grants were consistently prioritised, however there was notable diversity among the remaining payment types. Payments for joint working varied from 19.61% of all payments in Wales, and only 2.29% in Northern Ireland; fees for service and consultancy varied from 33.78% of all payments in Scotland, to 4.86% in Northern Ireland; and contributions to costs of events ranged from 31.87% in Northern Ireland, to 18.58% in Wales.

Figure 2. Percentage of total value for each payment type

A Kruskall-Wallis test also found a statistically significant difference between the distribution of payments for costs of events $\chi2(3) = 89.680$, $p = .000$, which were lowest in Wales and highest in Northern Ireland, and donations and grants $\chi2(3) = 31.698$, $p = <.000$, which were lowest in Northern Ireland and highest in England. Differences in fees for service and consultancy ($p = .995$) and joint working ($p = .261$) were non-significant. Post-hoc analyses revealed the differences in payments for costs of events were significant between every country pairing and between three country pairings for donations and grants (see Supplementary File 11).

DISCUSSION
Our findings show that the pharmaceutical industry's payment strategies and targeting of certain recipient categories differ across the four UK countries. This confirms concerns that previous analysis of industry payments at the UK level[11] resulted in conclusions representative of England only, an important oversight as key decisions about commissioning of health services are taken within each country[55 56].

Our analysis also confirms transparency concerns about duplicate payments and payments disclosed on the incorrect platform within the industry's self-regulatory system[30 60]. Although the UK's self-regulatory system is the most robust in Europe[42], better integration between Disclosure UK and patient organisation disclosures would eliminate double- and under-reporting as well as opacity about where something is reported. We know that drug companies under-report payments to patient organisations[30] and our findings indicate that some instances may be explained by confusion about where to report.

The prioritised recipient categories indicate diverse funding strategies in each country. Scotland and Wales were targeted at the "upstream" policy level (where policies are set), namely healthcare commissioning, planning and regulatory organisations such as NHS Boards (Scotland) and Health Boards (Wales), while England and Northern Ireland were targeted "downstream" (where policies are enacted), namely public sector secondary and tertiary care providers, such as hospitals, in England, and public sector primary care providers, such as GP surgeries, in Northern Ireland. These distinctions reflect the health system setup in each country, as in Scotland and Wales Health Boards are responsible for both the purchase and provision of health services, whereas in England and Northern Ireland these responsibilities are separate[61 62].

For peer review only - http://bmjopen.bmj.com/site/about/guidelines.xhtml

Professional organisations were also prioritised in England, Scotland and Northern Ireland, mirroring observations of frequent individual COIs held by professional organisations in the UK[11], Canada and the US[63 64]. These organisations comprise key opinion leaders in specific health fields which drug companies have been known to seek to influence in order to shape the background of medical opinion[65-67], and their members play an important role in the development of US[63 68] and Japanese[69] clinical practice guidelines. Education and research providers, particularly universities, were also prioritised in England and Scotland where, incidentally, all the UK's top universities are located[70]. New pharmaceuticals frequently originate in universities[71], and providing payments gives industry the opportunity to exert greater control over the research process[66]. Both professional organisations[69] and universities[72] are also central providers of continuing medical education (CME), but industry financial involvement has been criticised as unregulated and biased promotional activity[73 74]. Direct comparison to healthcare organisations also revealed patient organisations as a relatively major target of payments, particularly in England and Scotland. Funding various stakeholders forms part of the web of influence[2 3], allowing companies to influence public and policy opinion[75 76]. Customarily, industry has targeted prescribers, however non-prescribing stakeholders have become increasingly important given their considerable influence over prescribing decisions[77] and involvement in policymaking[3].

The number of recipients in common, whereby higher results imply a significant weighting of interest and influence, was highest in England and reduced in parallel to population size in the remaining three countries. These connections are recognised by the pharmaceutical industry which draws on prescribing and physician data to target their own payments[78 79 80] and identify organisations to inform marketing strategies[81 82], suggesting that connections through common recipients are not accidental. In England, funding healthcare providers evidently brings benefits given the prevalence of multiple donors per recipient. This pattern might also in part be a function of the number of research-active and national centres of excellence located in England, meaning that service providers might be very effective at getting donor funds, whereas in the smaller countries with perhaps fewer organisations operating on a national scale, the funding is more targeted and follows fewer overarching patterns. Additional benefits evidently exist in trying to influence strategic organisations in Scotland and Wales to shape future policies and service provision. In Northern Ireland, the smallest UK country, the high number of common primary care providers recipients may indicate they were being used as an experiment for the privatisation of primary care or for the establishment of additional primary care services. In the smaller countries, these patterns of common recipients potentially pose a greater risk to exposing vulnerabilities to collusion given the much smaller population each recipient serves.

Although professional and patient organisations were generally prioritised financially, recipients in common was infrequent. This may reflect these organisations being more tightly related to companies with specific drug or disease portfolios[83] than categories with higher density scores such as primary care providers which appeal to a broader spectrum of companies. The relatively low density scores for patient organisation are also concerning given that the ABPI Code provisions prohibit companies from being the sole funder of a patient organisation[57].

For peer review only - http://bmjopen.bmj.com/site/about/guidelines.xhtml

Our findings indicated that different payment types were prioritised in each country, although donations and grants, such as medical and educational goods, were prioritised in every consistently, mirroring payments in Ireland[14] and the UK[11]. Payments for joint working, initiatives involving shared investment by the NHS and drug companies[84], were prioritised most in Wales, raising concerns around the extent of drug company involvement in Welsh health services. Compared to England, there is very little private sector involvement in healthcare provision in Wales[85], however our analysis suggests this does not mean that industry is not heavily involved in healthcare planning and delivery. Joint working casts a veil over NHS underfunding[86] which may explain the prevalence in Wales given it's NHS is the most underfunded in the UK[87]. The prioritisation of fees for service and consultancy in Scotland may reflect the Scottish NHS priorities which include increasing the role of professionals in resource allocation[88]. Finally, costs of events, such as science or medical focused conferences and educational events[89], were prioritised most in Northern Ireland, however we know industry-sponsored CME can be biased[73], therefore greater policy attention needs to be placed on monitoring these activities in Northern Ireland.

Strengths and limitations

This is the first study to jointly analyse payments to healthcare and patient organisations, which was made possible by the current UK transparency provisions. It also is the first of its kind to explore payments across the four UK countries. To date, the spotlight has been on individual COIs, however this threatens to downplay the systemic problem of a broader institutional culture whereby industry funding is embraced and industry interests can be advanced[3 90]. However, it our study has limitations. We focus only on 2015 data due to the substantial time required to prepare Disclosure UK data for effective analysis, particularly categorising recipients to make them distinguishable and identifying recipient countries. We can assume the patterns are maintained over time as the overall payment values have remained stable each year[29 91], however longitudinal analysis would confirm this. Also, we could not determine whether sharing recipients was accidental or intentional, nor did we measure the impact of these payments.

Conclusions and policy implications

Our findings raise important questions regarding the risk of institutional COIs, particularly as the interests of patients and industry can be at odds[67]. Regional variability in payments has implications for subnational policymaking[40]. The variable prioritisation and significant differences in the distribution of payments to each recipient category suggests that companies strategically target recipients in individual countries based on policy responsibilities in each national government, perhaps following an assessment of the benefits of supporting particular organisations.

The transparency issues we identified show the self-regulatory system is disjointed and requires better integration through a standardised database. As a minimum, compulsory recipient identifiers should be introduced. Disclosure UK needs to introduce detailed payment descriptions and outline each recipient's total annual income, currently a non-obligatory suggestion in the ABPI Code for payments to patient organisations[92]. This is especially pertinent to smaller recipients, for example in Northern Ireland, which may have

For peer review only - http://bmjopen.bmj.com/site/about/guidelines.xhtml

fewer funding options and therefore may be prone to dependency on industry funding[43]. Echoing calls in the US for state-specific disclosure policies[40], Disclosure UK and disclosures of payments to patient organisations need to be adapted to better capture the distinction between payments in England, Scotland, Wales, and Northern Ireland.

However, although transparency is important it is not a panacea for the implicit bias caused by payments[93] or the corrosive impact financial COIs can have on the delivery of quality healthcare[23 94]. Identifying COIs is the first step[90 95], but responding to them needs to come next[23]. In lieu of a response to calls for total eradication of industry payments[94 96], our findings might inform future policies to mitigate against the risks posed by institutional COIs. In particular, recipient categories susceptible to high shares of industry funding in each country command increased regulatory oversight in the form of specific guidelines provided to companies and recipients by the ABPI.

Our findings indicate payment strategies, particularly targeted recipients and payment types, differ between countries. Acknowledging sub-national institutional COIs is a crucial first step to better inform policy responses and reforms at the sub-national level. Future analysis of industry payments should explore these payments longitudinally.

For peer review only

Ethical approval

The study did not require ethical approval (as it draws on publicly available data at the organisational level), however it is part of a bigger project which has ethical approval from the University of Bath's Social Sciences Research Ethics Committee (approval code: S19-073).

Funding statement

ER's work was supported by a +3 PhD Studentship award match-funded (50%) by the Economic and Social Research Council and the University of Bath. This work forms part of the PhD project. PO's work was supported by grants from The Swedish Research Council for Health, Working Life and Welfare (FORTE), no. 2016-00875, and The Swedish Research Council (VR), no. 2020-01822.

Competing interests statement

ER has no conflicts of interests to declare. PO's PhD student was supported by a grant from Sigma Pharmaceuticals, a UK pharmacy wholesaler and distributor (not a pharmaceutical company). The PhD work funded by Sigma Pharmaceuticals is unrelated to the subject of this paper.

Author contributions

ER designed, managed, analysed and interpreted the data, as well as drafted the article. PO conceived and designed the study, provided supervision, and drafted the article.

Data statement

Underlying data will be made publicly available on the Bath Research Data Archive upon publication.

For peer review only

REFERENCES

1. Sismondo S. Ghost-managed medicine: Big pharma's invisible hands: Mattering Press 2018:8.
2. Rickard E, Ozieranski P. A hidden web of policy influence: The pharmaceutical industry’s engagement with UK’s All-Party Parliamentary Groups. *PLOS ONE* 2021;16(6):e0252551. doi: 10.1371/journal.pone.0252551
3. Marks JH. The perils of partnership: industry influence, institutional integrity, and public health: Oxford University Press 2019.
4. Agrawal S, Brennan N, Budetti P. The Sunshine Act—effects on physicians. *N Engl J Med* 2013;2013(368):2054-57.
5. Ahmed R, Bae S, Hicks CW, et al. Here Comes the Sunshine: Industry's Payments to Cardiothoracic Surgeons. *Annals of Thoracic Surgery* 2017;103(2):567-72. doi: 10.1016/j.athoracsur.2016.06.053
6. Braithwaite J, Frane N, Partan MJ, et al. Review of Industry Payments to General Orthopaedic Surgeons Reported by the Open Payments Database: 2014 to 2019. *JAAOS Global Research & Reviews* 2021;5(5)
7. Fleischman W, Ross JS, Melnick ER, et al. Financial Ties Between Emergency Physicians and Industry: Insights From Open Payments Data. *Annals of Emergency Medicine* 2016;68(2):153-58.e4. doi: 10.1016/j.annemergmed.2016.01.014
8. Maruf M, Sidana A, Fleischman W, et al. Financial Relationships between Urologists and Industry: An Analysis of Open Payments Data. *Urology Practice* 2018;5(3):180-86. doi: 10.1016/j.urpr.2017.03.012
9. Slentz DH, Nelson CC, Lichter PR. Characteristics of Industry Payments to Ophthalmologists in the Open Payments Database. *JAMA Ophthalmology* 2019;137(9):1038-44. doi: 10.1001/jamaophthalmol.2019.2456
10. Moynihan R, Fabbri A, Parker L, et al. Mixed methods evaluation of workshops for citizen health advocates about financial conflicts of interests in healthcare. *BMJ Open* 2020;10(5) doi: 10.1136/bmjopen-2019-034195
11. Ozieranski P, Csanadi M, Rickard E, et al. Analysis of Pharmaceutical Industry Payments to UK Health Care Organizations in 2015. *JAMA Network Open* 2019;2(6):e196253-e53. doi: 10.1001/jamanetworkopen.2019.6253
12. Parker L, Karanges EA, Bero L. Changes in the type and amount of spending disclosed by Australian pharmaceutical companies: an observational study. *BMJ open* 2019;9(2):e024928.
13. Fabbri A, Grundy Q, Mintzes B, et al. A cross-sectional analysis of pharmaceutical industry-funded events for health professionals in Australia. *BMJ open* 2017;7(6):e016701.
14. Moriarty F, Larkin J, Fahey T. Payments reported by the pharmaceutical industry in Ireland from 2015 to 2019: an observational study. *Health Policy* 2021 doi: https://doi.org/10.1016/j.healthpol.2021.07.016
15. Ozaki A, Saito H, Senoo Y, et al. Overview and transparency of non-research payments to healthcare organizations and healthcare professionals from pharmaceutical companies in Japan: Analysis of payment data in 2016. *Health Policy* 2020;124(7):727-35. doi: https://doi.org/10.1016/j.healthpol.2020.03.011

For peer review only - http://bmjopen.bmj.com/site/about/guidelines.xhtml

16. Ozaki A, Saito H, Onoue Y, et al. Pharmaceutical payments to certified oncology specialists in Japan in 2016: a retrospective observational cross-sectional analysis. *BMJ Open* 2019;9(9):e028805. doi: 10.1136/bmjopen-2018-028805
17. Anderson TS, Gellad WF, Good CB. Characteristics of biomedical industry payments to teaching hospitals. *Health Affairs* 2020;39(9):1583-91. doi: 10.1377/hlthaff.2020.00385
18. Rothman SM, Raveis VH, Friedman A, et al. Health advocacy organizations and the pharmaceutical industry: an analysis of disclosure practices. *American journal of public health* 2011;101(4):602-09.
19. McCoy MS, Carniol M, Chockley K, et al. Conflicts of interest for patient-advocacy organizations. *New England Journal of Medicine* 2017;376(9):880-85.
20. Rose SL, Highland J, Karafa MT, et al. Patient Advocacy Organizations, Industry Funding, and Conflicts of Interest. *JAMA Internal Medicine* 2017;177(3):344-50. doi: 10.1001/jamainternmed.2016.8443
21. Schafer A. Institutional conflict of interest: attempting to crack the deferiprone mystery. *Journal of Medical Ethics* 2021;47(8):531. doi: 10.1136/medethics-2019-105498
22. Lo YL. Further insights on institutional conflicts of interest in research settings. *Proceedings of Singapore Healthcare* 2020;29(1):3-4. doi: 10.1177/2010105820909983
23. Lo B, Field MJ. Conflict of interest in medical research, education, and practice. 2009
24. Lemmens T, Miller PB. The human subjects trade: ethical and legal issues surrounding recruitment incentives. *J Law Med Ethics* 2003;31(3):398-418. doi: 10.1111/j.1748-720x.2003.tb00103.x [published Online First: 2003/11/25]
25. Olivieri NF. Patients' health or company profits? The commercialisation of academic research. *Science and Engineering Ethics* 2003;9(1):29-41.
26. Ozieranski P, Martinon, L., Jachiet, P., Mulinari, S. Accessibility and quality of drug company disclosures of payments to healthcare professionals and organisations in 37 countries: A European policy review. *BMJ Open* 2021
27. Fabbri A, Parker L, Colombo C, et al. Industry funding of patient and health consumer organisations: systematic review with meta-analysis. *BMJ* 2020;368:l6925. doi: 10.1136/bmj.l6925
28. EFPIA. EFPIA CODE OF PRACTICE ON RELATIONSHIPS BETWEEN THE PHARMACEUTICAL INDUSTRY AND PATIENT ORGANISATIONS, 2011.
29. Ozieranski P, Rickard E, Mulinari S. Exposing drug industry funding of UK patient organisations. *Bmj* 2019;365:l1806. doi: 10.1136/bmj.l1806 [published Online First: 2019/05/28]
30. Ozieranski P, Csanadi M, Rickard E, et al. Underreporting of drug industry payments to patient organisations in the UK (2012-2016). 2020
31. Mulinari S, Vilhelmsson A, Rickard E, et al. Five years of pharmaceutical industry funding of patient organisations in Sweden: Cross-sectional study of companies, patient organisations and drugs. *PLOS ONE* 2020;15(6):e0235021. doi: 10.1371/journal.pone.0235021
32. Pashley D OP, Mulinari S. . Disclosure of pharmaceutical industry funding of patient organizations in Nordic countries: can industry self-regulation deliver on its transparency promise? *International Journal of Health Services* 2022
33. EFPIA. The EFPIA Disclosure Code: Your Questions Answered, 2014.

For peer review only - http://bmjopen.bmj.com/site/about/guidelines.xhtml

34. Mulinari S, Ozieranski P. Disclosure of payments by pharmaceutical companies to healthcare professionals in the UK: analysis of the Association of the British Pharmaceutical Industry's Disclosure UK database, 2015 and 2016 cohorts. *BMJ open* 2018;8(10):e023094.
35. Keller AC, Packel L. Going for the cure: patient interest groups and health advocacy in the United States. *J Health Polit Policy Law* 2014;39(2):331-67. doi: 10.1215/03616878-2416238 [published Online First: 2013/12/07]
36. Hegde D, Sampat B. Can private money buy public science? Disease group lobbying and federal funding for biomedical research. *Management Science* 2015;61(10):2281-98.
37. Niforatos JD, Boas S, Hertz S, et al. Analysis of current financial relationships between emergency physicians and industry. *American Journal of Emergency Medicine* 2019;37(4):722-25. doi: 10.1016/j.ajem.2018.12.039
38. Cheng T, Boelitz K, Rybin D, et al. Nationwide patterns in industry payments to academic vascular surgeons. *Journal of Vascular Surgery* 2021;73(2):675-81. doi: 10.1016/j.jvs.2020.04.527
39. Garstka ME, Monlezun D, DuCoin C, et al. The Sunshine Act and surgeons: a nation-wide analysis of industry payments to physicians. *Journal of Surgical Research* 2019;233:41-49. doi: 10.1016/j.jss.2018.07.004
40. Cuomo RE, Cai M, Shah N, et al. Physicians payment in the United States between 2014 and 2018: An analysis of the CMS Open Payments database. *PLOS ONE* 2021;16(6):e0252656. doi: 10.1371/journal.pone.0252656
41. Mejia J, Mejia A, Pestilli F. Open data on industry payments to healthcare providers reveal potential hidden costs to the public. *Nature Communications* 2019;10(1) doi: 10.1038/s41467-019-12317-z
42. Mulinari S, Martinon L, Jachiet P-A, et al. Pharmaceutical industry self-regulation and non-transparency: country and company level analysis of payments to healthcare professionals in seven European countries. *Health Policy* 2021;125(7):915-22. doi: https://doi.org/10.1016/j.healthpol.2021.04.015
43. Ozieranski P, Pitter J, Rickard E, et al. A "patient-industry complex"? Investigating the financial dependency of UK patient organisations on drug company funding. *Sociology of Health & Illness* 2021
44. EFPIA. The Pharmaceutical Industry in Figures, 2016.
45. Hyndman N. Editorial: The charity sector— changing times, changing challenges. *Public Money & Management* 2017;37(3):149-53. doi: 10.1080/09540962.2017.1281608
46. Greer SL. Territorial politics and health policy: UK health policy in comparative perspective: Manchester University Press 2004.
47. Birrell D. Comparing devolved governance: Palgrave Macmillan 2012.
48. Cheung A, Paun A, Valsamidis L. Devolution at 20. Institute for Government, 2019.
49. ONS. Population estimates for the UK, England and Wales, Scotland and Northern Ireland: mid-2015 2016 [Available from: https://www.ons.gov.uk/peoplepopulationandcommunity/populationandmigration/populationestimates/bulletins/annualmidyearpopulationestimates/mid2015 accessed 13th September 2021.
50. Office for National Statistics. Life expectancy for local areas of the UK: between 2001 to 2003 and 2017 to 2019 2020 [Available from: https://www.ons.gov.uk/peoplepopulationandcommunity/healthandsocialcare/heal

For peer review only

thandlifeexpectancies/bulletins/lifeexpectancyforlocalareasoftheuk/between2001to2003and2017to2019 accessed 28th August 2021.
51. The Pharmaceutical Journal. Wales has highest number of prescriptions per head of population among UK countries 2021 [Available from: https://pharmaceutical-journal.com/article/news/wales-has-highest-number-of-prescriptions-per-head-of-population-among-uk-countries accessed 30th November 2021.
52. Lexchin J. Pharmaceutical company spending on research and development and promotion in Canada, 2013-2016: a cohort analysis. *J Pharm Policy Pract* 2018;11:5-5. doi: 10.1186/s40545-018-0132-3
53. Ozieranski P, King LP. Governing drug reimbursement policy in Poland: The role of the state, civil society, and the private sector. *Theory and Society* 2017;46(6):577-610. doi: 10.1007/s11186-017-9300-8
54. Orientation Marketing Insights. The Pharmaceutical Marketer's Guide.
55. Moberly T. CCGs fail to declare pharma funding. *BMJ* 2018;360:j5911. doi: 10.1136/bmj.j5911
56. Saghy E, Mulinari S, Ozieranski P. Drug company payments to General Practices in England: cross-sectional and social network analysis. *PLOS ONE* 2021
57. Association of the British Pharmaceutical Industry. CODE OF PRACTICE for the PHARMACEUTICAL INDUSTRY 2015, 2015.
58. Snijders TA, Faye M, Brailly J. Network dynamics with a nested node set: Sociability in seven villages in Senegal. *Statistica Neerlandica* 2020;74(3):300-23.
59. Borgatti SP, Everett, M.G. and Freeman, L.C. . Ucinet for Windows: Software for Social Network Analysis. Harvard, MA: Analytic Technologies 2002.
60. Rickard E, Ozieranski P, Mulinari S. Evaluating the transparency of pharmaceutical company disclosure of payments to patient organisations in the UK. *Health Policy* 2019;123(12):1244-50.
61. Greer SL. Devolution and health in the UK: policy and its lessons since 1998. *British Medical Bulletin* 2016;118(1):16.
62. Harker R. NHS Funding and Expenditure. Commons Library Briefing, 2019.
63. Neuman J, Korenstein D, Ross JS, et al. Prevalence of financial conflicts of interest among panel members producing clinical practice guidelines in Canada and United States: cross sectional study. *BMJ* 2011;343:d5621. doi: 10.1136/bmj.d5621
64. Moynihan R, Albarqouni L, Nangla C, et al. Financial ties between leaders of influential US professional medical associations and industry: Cross sectional study. *The BMJ* 2020;369 doi: 10.1136/bmj.m1505
65. Sismondo S. Key opinion leaders and the corruption of medical knowledge: what the Sunshine Act will and won't cast light on: SAGE Publications Sage CA: Los Angeles, CA, 2013.
66. Madureira Lima J, Galea S. Corporate practices and health: a framework and mechanisms. *Globalization and Health* 2018;14(1):21. doi: 10.1186/s12992-018-0336-y
67. House of Commons Health Committee. The Influence of the Pharmaceutical Industry, 2005.
68. Mitchell AP, Basch EM, Dusetzina SB. Financial relationships with industry among national comprehensive cancer network guideline authors. *JAMA Oncology* 2016;2(12):1628-31. doi: 10.1001/jamaoncol.2016.2710

For peer review only - http://bmjopen.bmj.com/site/about/guidelines.xhtml

69. Saito H, Tani Y, Ozaki A, et al. Financial ties between authors of the clinical practice guidelines and pharmaceutical companies: an example from Japan. *Clinical Microbiology and Infection* 2019;25(11):1304-06. doi: https://doi.org/10.1016/j.cmi.2019.07.025
70. The Guardian. University league table 2015 - the complete list 2014 [Available from: https://www.theguardian.com/education/ng-interactive/2014/jun/02/university-league-tables-2015-the-complete-list accessed 6th December 2021.
71. Nayak RK, Avorn J, Kesselheim AS. Public sector financial support for late stage discovery of new drugs in the United States: cohort study. *BMJ* 2019;367:l5766. doi: 10.1136/bmj.l5766
72. Angell M. The truth about the drug companies: How they deceive us and what to do about it: Random House Incorporated 2005.
73. Fugh-Berman A. Industry-funded medical education is always promotion—an essay by Adriane Fugh-Berman. *BMJ* 2021;373:n1273. doi: 10.1136/bmj.n1273
74. Spithoff S. Industry involvement in continuing medical education: time to say no. *Can Fam Physician* 2014;60(8):694-703.
75. Pereira Gray D, White E, Russell G. Medicalisation in the UK: changing dynamics, but still ongoing. *Journal of the Royal Society of Medicine* 2015;109(1):7-11. doi: 10.1177/0141076815600908
76. Abraham J. The sociological concomitants of the pharmaceutical industry and medications. *Handbook of medical sociology* 2010;6:290-308.
77. Bhalla G, Evgeniou T, Lerer L. Customer Relationship Management and Networked Healthcare in the Pharmaceutical Industry. *Journal of Medical Marketing* 2004;4(4):370-79. doi: 10.1057/palgrave.jmm.5040188
78. Heesters M. An assault on the business of pharmaceutical data mining. *U Pa J Bus L* 2008;11:789.
79. Sullivan T. Open Payments for All – Cresen Solutions Launches PowerCMS™ 2019 [Available from: https://www.policymed.com/2019/06/open-payments-for-all-cresen-solutions-launches-powercms.html accessed 30th November 2021.
80. Anand R CD, Yan J, et al. Leveraging CMS Open Payments Data to Identify Channel Preferences and Gather Competitive Intelligence, Thereby Improving HCP Targeting. *Journal of the Pharmaceutical Management Science Association* 2017 doi: https://www.pmsa.org/jpmsa-vol05-article06
81. VOXX Analytics. VOXX Products 2017 [Available from: http://www.voxxanalytics.com/products accessed July 2019.
82. Hollands S. Receipt of Promotional Payments at the Individual and Physician Network Level Associated with Higher Branded Antipsychotic Prescribing Rates. *Adm Policy Ment Health* 2020;47(1):73-85. doi: 10.1007/s10488-019-00974-7
83. Edgar A. The dominance of big pharma: power. *Medicine, Health Care and Philosophy* 2013;16(2):295-304. doi: 10.1007/s11019-012-9385-9
84. Association of the British Pharmaceutical Industry. Joint Working: A toolkit for industry and the NHS, 2019.
85. Timmins N. The four UK health systems. Learning from each other: The King's Fund, 2013.
86. Moberly T. NHS joint working with industry is out of public sight. *BMJ* 2019;364:l1353. doi: 10.1136/bmj.l1353

For peer review only

87. Paun A, Cheung A, Nicholson E. Funding Devolution: The Barnett formula in theory and practice. In: Government IF, ed., 2021.
88. Greer SL. Four way bet: how devolution has led to four different models for the NHS: Constitution Unit London 2004.
89. Bayer. Methodology Note, 2021.
90. Marks JH. Lessons from Corporate Influence in the Opioid Epidemic: Toward a Norm of Separation. *Journal of Bioethical Inquiry* 2020;17(2):173-89. doi: 10.1007/s11673-020-09982-x
91. ABPI. Disclosure UK 2022 [Available from: https://www.abpi.org.uk/reputation/disclosure-uk/.
92. Association of the British Pharmaceutical Industry. CODE OF PRACTICE for the PHARMACEUTICAL INDUSTRY 2019, 2019.
93. Basken P. Why disclosure policies don't discourage academe's drug salesmen. 2017 [Available from: https://www.chronicle.com/article/why-disclosure-policies-dont-discourage-academes-drug-salesmen/ accessed 30th November 2021.
94. Lexchin J, Fugh-Berman A. A Ray of Sunshine: Transparency in Physician-Industry Relationships Is Not Enough. *Journal of General Internal Medicine* 2021 doi: 10.1007/s11606-021-06657-0
95. Bachynski KE, Goldberg DS. Time out: NFL conflicts of interest with public health efforts to prevent TBI. *Injury Prevention* 2018;24(3):180-84.
96. Wilson M. The Sunshine Act: commercial conflicts of interest and the limits of transparency. *Open Med* 2014;8(1):e10-e13.

For peer review only

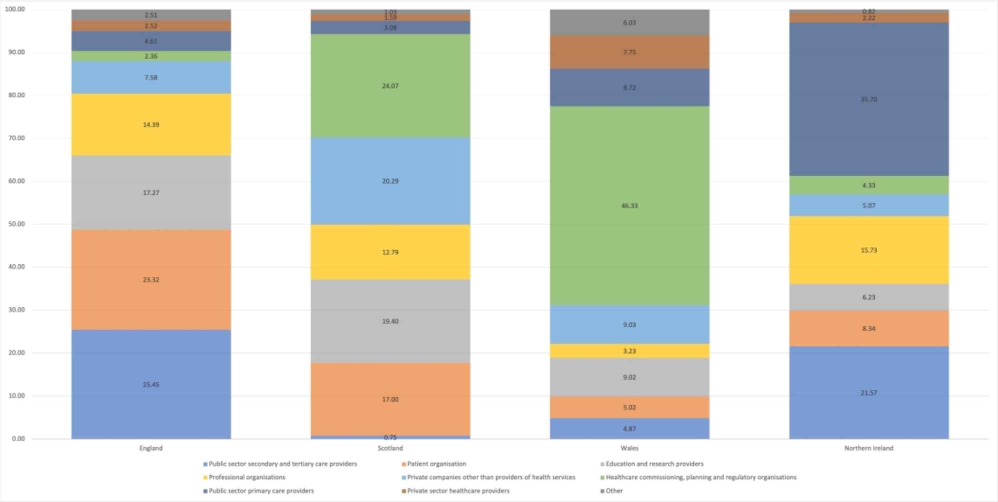

Percentage of payments to recipients in each category per country

442x222mm (300 x 300 DPI)

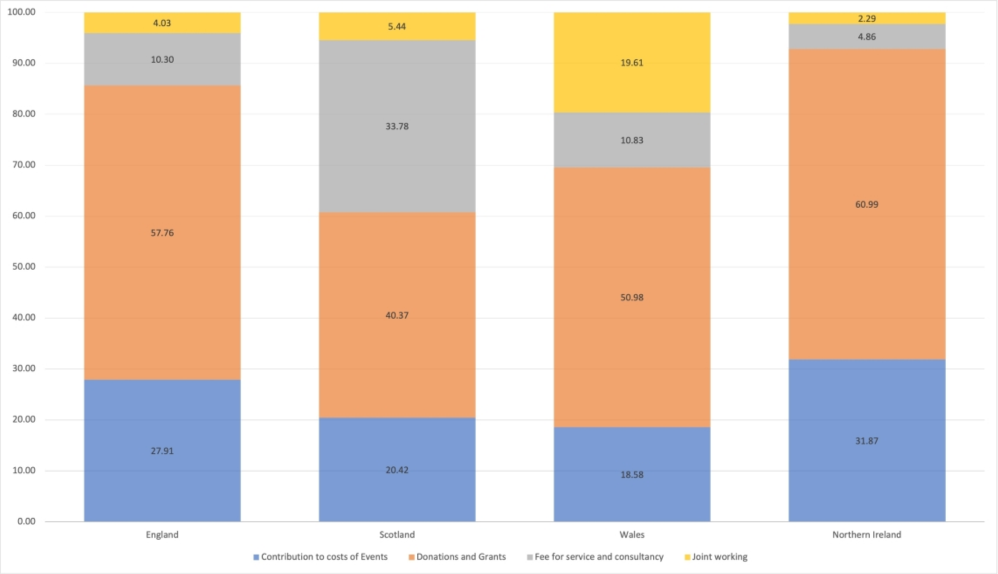

Percentage of total value for each payment type

314x181mm (300 x 300 DPI)

Supplementary file contents:

Supplementary File	Page
Supplementary File 1. Payment types included in Disclosure UK and patient organisation codes applied to Disclosure UK codes	2
Supplementary File 2. Recipient category descriptions and examples	3-4
Supplementary File 3. Additional details on network statistics used	5
Supplementary File 4. Data integration flowchart	6
Supplementary File 5. Top 10% of donors in each country	7
Supplementary File 6. Visualised networks for England (a), Scotland (b), Wales (c), Northern Ireland (d)	8-11
Supplementary File 7. Top ten companies by degree centrality scores in each country	12
Supplementary File 8. Descriptive statistics for each recipient category	13-15
Supplementary File 9. Top 10 recipients in each country	16-17
Supplementary File 10. Post-hoc Bonferroni pairwise comparisons between countries of payments per category	18-20
Supplementary File 11. Post-hoc Bonferroni pairwise comparisons between countries of payment types	21

For peer review only

Supplementary File 1. Payment types included in Disclosure UK and patient organisation codes applied to Disclosure UK codes

Payment type	Description of payment type	Patient organisation payments subsumed within the payment type
Contribution to costs of Events	Contribution to costs related to Events, through HCOs or Third Parties, including support to HCPs to attend Events, such as:<br>• Registration fees;<br>• Sponsorship agreements with HCOs or with Third Parties appointed by an HCO to manage an Event; and<br>• Travel and accommodation (EFPIA Code of Practice 2019, p. 30)	contributions to costs of events organised by recipients or third parties; travel, accommodation and registration fees
Donations and Grants to HCOs	Donations and Grants to HCOs that support healthcare, including donations and grants (either cash or benefits in kind) to institutions, organisations or associations that are comprised of HCPs and/or that provide healthcare (EFPIA Code of Practice 2019, p. 30)	donations; grants; corporate member, supporter, sponsor or partner; purchases and subscriptions from patient organisations; more than one distinct payment form; form of funding unclear; sponsorships
Fee for service and consultancy	Payments resulting from or related to contracts between Member Companies and HCOs under which such HCOs provide any type of services to a Member Company or any other type of funding not covered in the previous categories. Fees, on the one hand, and on the other hand payments relating to expenses agreed in the written agreement covering the activity will be disclosed as two separate amounts. (EFPIA Code of Practice 2019, p. 30)	fees for service and consultancy (including travel and accommodation); support, help and contributions
Joint working	The Department of Health defines joint working between the NHS and the pharmaceutical industry as situations where, for the benefit of patients, one or more pharmaceutical companies and the NHS pool skills, experience and/or resources for the joint development and implementation of patient centred projects and share a commitment to successful delivery. (ABPI Code of Practice 2015, Clause 20, p. 30)	n/a

For peer review only

Supplementary File 2. Recipient category descriptions and examples

Recipient category	Category description	Examples (country-specific examples where applicable)
Alternative providers of health services	Charities, not-for-profit companies, social enterprises and community interest companies providing health services	- social enterprise delivering primary or secondary care health services<br>- nursing or care home run by a community interest company (CIC)<br>- hospital, hospice, or nursing home with a charitable status
Education and research providers	Universities, charities, and noncommercial institutes undertaking research	- university<br>- research institute at a NHS organisation<br>- charity focusing on undertaking medical research
Formal bodies representing healthcare professionals or patients	Local medical, optical, optometric, or pharmaceutical committees and statutory bodies representing healthcare professionals or patients	- local medical committees (LMC)<br>- local optical or optometric committee (LOC)<br>*England*<br>- local pharmaceutical committee (LPC)
Charities and other third-sector organisations (excluding providers of health services and professional organisations)	Organisations (not patient organisations) focusing on education, research, advocacy, and multipurpose organisations	- third-sector organisation (non-charity) or charity focused on funding medical research<br>- Multipurpose third-sector organisation (non-charity)<br>- charity focused on providing funding or material support to patients or NHS organisations
Healthcare commissioning, planning and regulatory organisations	Local, regional, and commissioning, planning, or regulatory organisations	- primary care trust (PCT)<br>- NHS Shared Business Services<br>- National Institute for Health and Care Excellence (NICE)<br>- Public Health England<br>*England*<br>- Clinical commissioning group<br>- Locality group<br>- Area prescribing committee<br>- Local commissioning group<br>- NHS England<br>*Scotland*<br>- Regional NHS board<br>- Area pharmaceutical committee<br>*Wales*<br>- Health board<br>- Public health Wales<br>*Northern Ireland*

For peer review only

		- Health and social care board<br>- Local commissioning group
Patient organisations	Organisations focusing on supporting education, research, advocacy, and multipurpose organisations	- Hospital charities
Private companies other than providers of health services	Providers of medical communications or training services, commercial or medical research services, and accountancy or consultancy services	- manufacturer or supplier of medical devices or technologies<br>- pharmacy wholesaler or distributor<br>- event management services<br>- journal or publishing company<br>- clinical or contract research organisation<br>- private laboratory
Private sector healthcare providers	Private clinics and hospitals, healthcare groups, and providers of dental, pharmacy, and optical services	- dental practice<br>- pharmacy or chemist<br>- opticians<br>- private clinic, surgery, practice, or hospital<br>- private company providing community health or social care services
Professional organisations	Organisations of medical professionals, other healthcare professionals, or non-healthcare professionals	- organisation of medical professionals (doctors)<br>- royal college - medical professionals<br>- alliance or coalition of professional associations or groups<br>- professional organisation of pharmacists or pharmacy technicians
Public administration and providers of public services	Central UK government bodies, devolved administrations in Scotland, Wales, and Northern Ireland, and local authorities	- district, city, country, or borough council<br>- prison<br>- devolved administrations<br>- central government bodies
Public sector primary care providers	General practitioner surgeries, medical practice centres, groups of surgeries or medical practices, and healthcare or medical groups	- GP practice, surgery, medical practice or family practice<br>- health centre, medical centre or primary care centre<br>- group of surgeries or medical practices
Public sector secondary and tertiary care providers	NHS trusts, NHS hospitals, and networks and collaboratives of NHS organisations	- NHS hospital<br>- NHS Foundation Trust<br>- NHS Trust<br>- strategic clinical network (SCN)<br>*Scotland*<br>- managed clinical network (MCN)

For peer review only - http://bmjopen.bmj.com/site/about/guidelines.xhtml

Supplementary File 3. Additional details on network statistics used

We calculated *density* scores, a measure of the overall level of connection within a network, which provides two network-level values – average value and average weighted degree. First, average value density computes the average tie strength, or the average number of recipients each *pair* of companies in the network shares[58]. Second, average weighted degree density computes the total number of ties for each company and then averages them. The higher the density scores, the more connected by recipients the companies are[59]. Density adjusts for the number of nodes, or companies, in the network, improving comparability between groups[60].

We also calculated *degree centrality*, which provides a score for each individual company based on the number of shared recipients it has with *all* other companies in the network. The higher the degree centrality, the more central the drug company is[61]. From a marketing perspective, this reveals the dominant companies that appear to purposely target key areas of shared interest through providing payments to the same recipients as other companies, which can, in turn, be compared between recipient countries and categories, as well as to the value of payments.

For peer review only

Supplementary File 4. Data integration flowchart

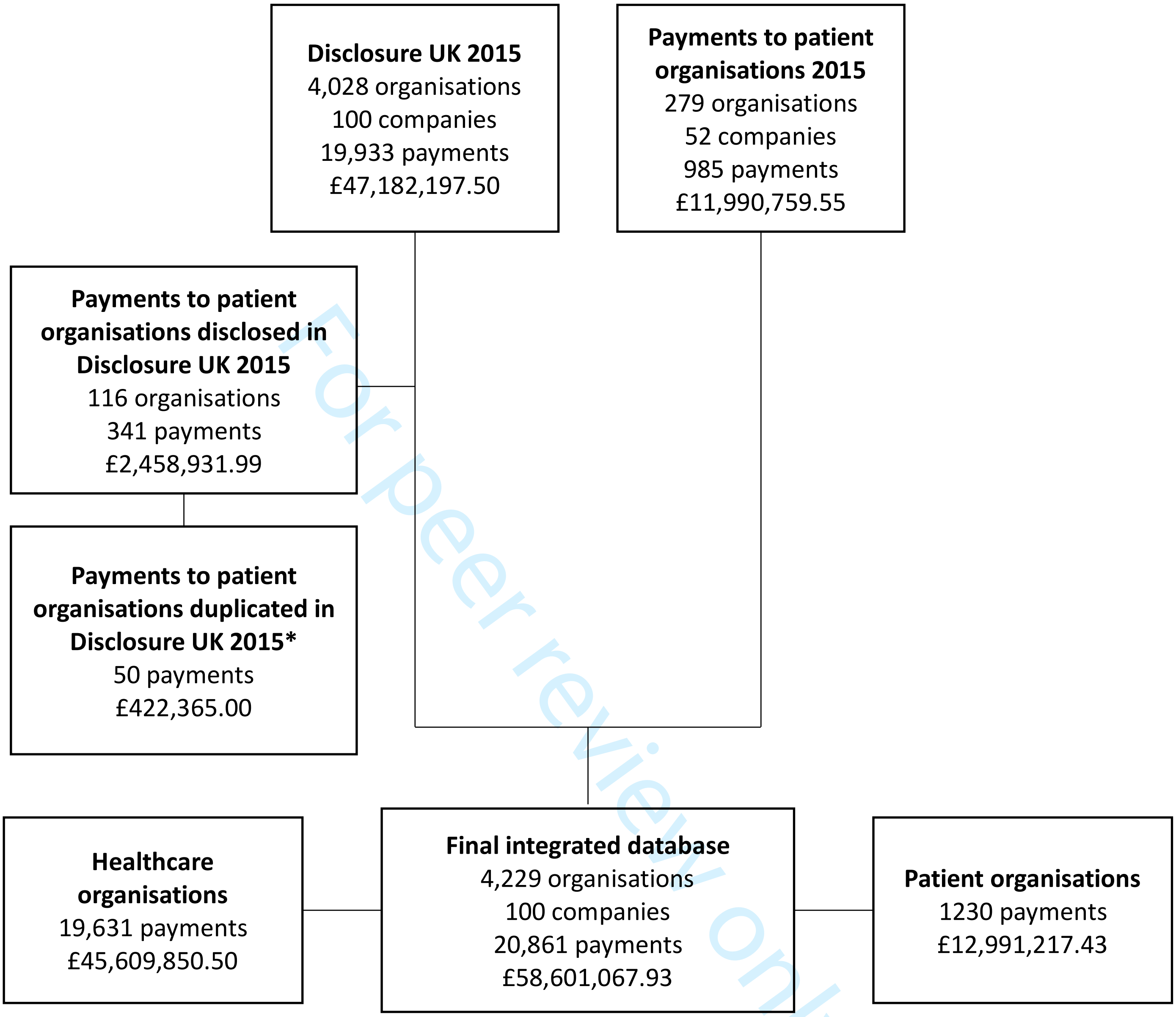

* This is the number and value of payments excluded to ensure no payment was counted twice

For peer review only

Supplementary File 5. Top 10% of donors in each country

Country	Company (revenue ranking)	Payment value - £ (%)	Payments – n (%)
England	Pfizer (2)	5,292,130.74 (10.09)	1636 (8.99)
	Novartis (7)	3,564,500.43 (6.80)	460 (2.53)
	Bayer (8)	3,476,304.44 (6.63)	2110 (11.60)
	GlaxoSmithKline (4)	3,291,496.35 (6.28)	1076 (5.92)
	AstraZeneca (1)	2,779,000.54 (5.30)	1279 (7.03)
	Janssen-Cilag (10)	2,387,242.64 (4.55)	722 (3.97)
	UCB Pharma (30)	2,204,967.90 (4.20)	74 (0.41)
	Astellas Pharma (21)	2,044,050.60 (3.90)	311 (1.71)
	Roche (5)	1,931,651.77 (3.68)	173 (0.95)
	Biogen Idec (23)	1,886,879.26 (3.60)	83 (0.46)
	Top 10% total	28,858,224.67 (55.03)	7924 (43.56)
Scotland	Biogen Idec (24)	733,104.05 (20.09)	7 (0.51)
	Takeda UK (38)	274,952.71 (7.53)	25 (1.82)
	Pfizer (2)	250,859.45 (6.87)	143 (10.44)
	Bayer (8)	215,930.76 (5.92)	164 (11.97)
	Novartis (7)	199,703.97 (5.47)	48 (3.50)
	Bristol-Myers Squibb (23)	183,959.00 (5.04)	51 (3.72)
	AstraZeneca (1)	178,848.49 (4.90)	75 (5.47)
	Top 10% total	2,037,358.42 (55.82)	513 (37.45)
Wales	Pfizer (2)	284,719.57 (14.32)	102 (10.30)
	Roche (5)	230,090.90 (11.58)	10 (1.01)
	Novartis (7)	177,069.59 (8.91)	36 (3.64)
	AstraZeneca (1)	148,288.67 (7.46)	79 (7.98)
	Janssen-Cilag (10)	122,237.44 (6.15)	22 (2.22)
	Biogen (24)	112,428.62 (5.66)	7 (0.71)
	Top 10% total	1,074,834.80 (54.07)	256 (25.86)
Northern Ireland	Sanofi Aventis (13)	92,252.80 (17.81)	24 (7.72)
	Pfizer (2)	86,639.31 (16.73)	45 (14.47)
	Napp Pharmaceuticals (29)	83,252.29 (16.07)	45 (14.47)
	Bayer (8)	37,959.50 (7.33)	75 (24.12)
	Top 10% total	300,103.90 (57.94)	189 (60.77)

*Value as proportion of all payments in each country

For peer review only - http://bmjopen.bmj.com/site/about/guidelines.xhtml

Supplementary File 6. Visualised networks for England (a), Scotland (b), Wales (c), Northern Ireland (d)

Supplementary File 5a. England's network

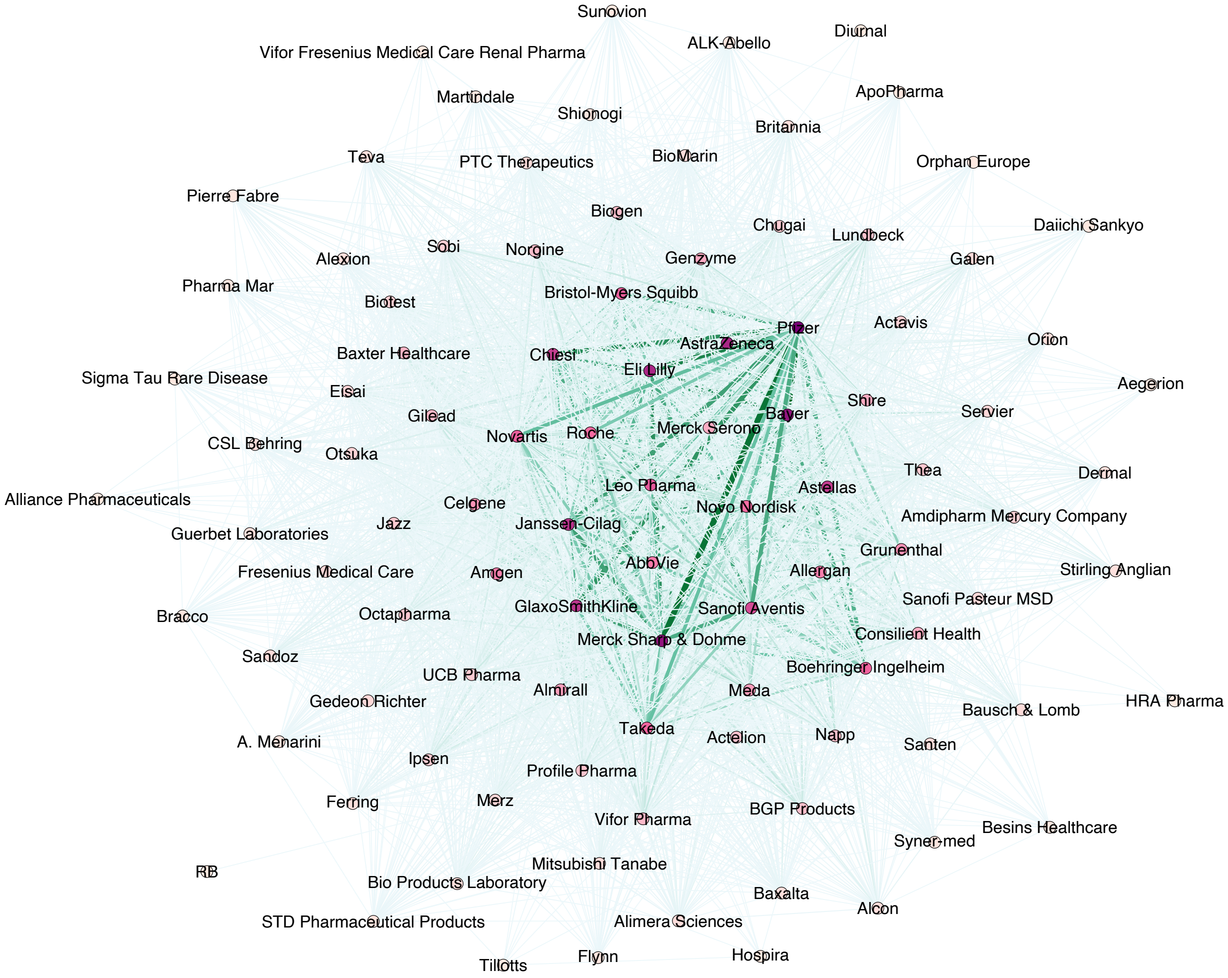

For peer review only - http://bmjopen.bmj.com/site/about/guidelines.xhtml

Supplementary File 6b. Scotland's network

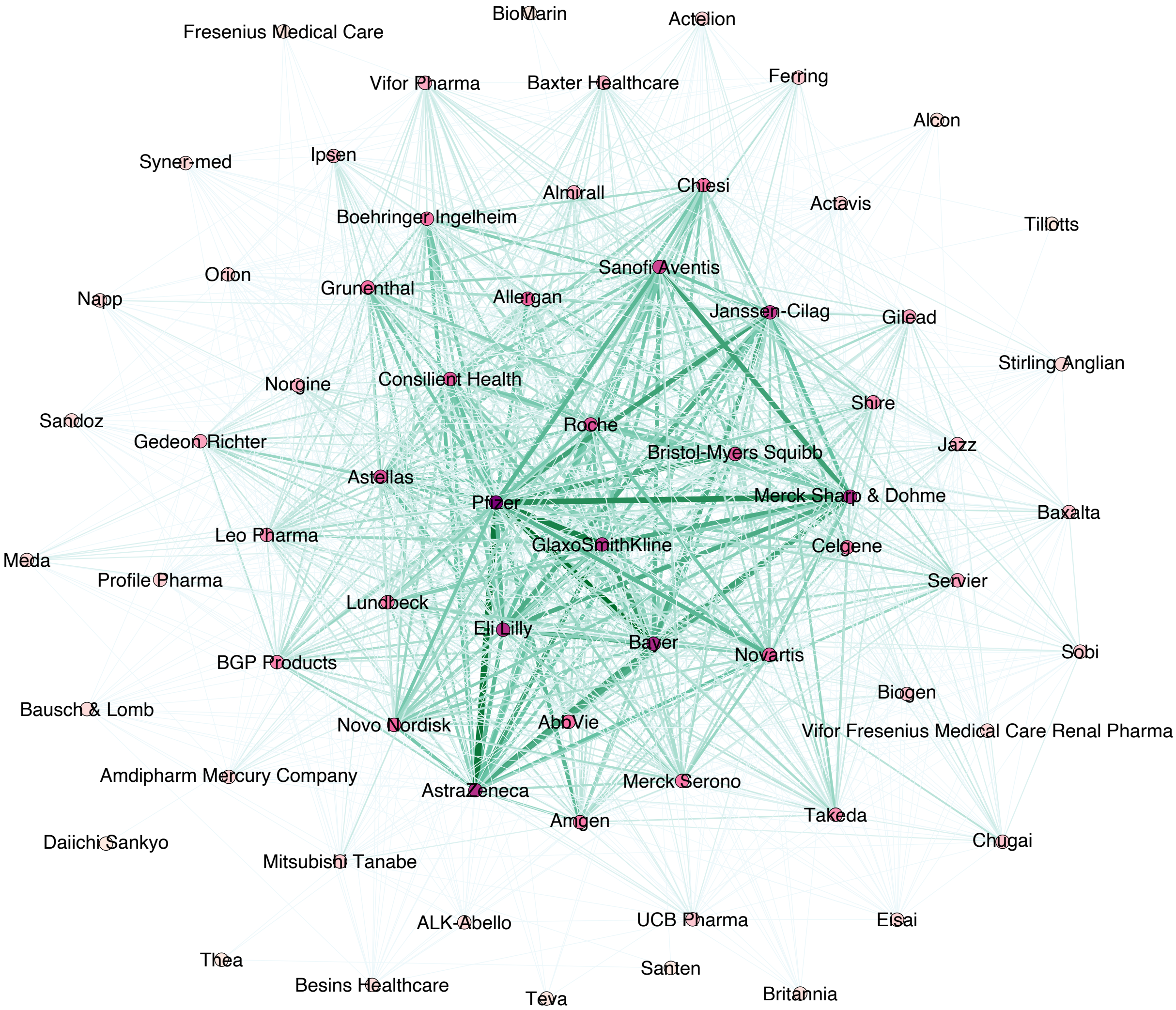

Supplementary File 6c. Wales' network

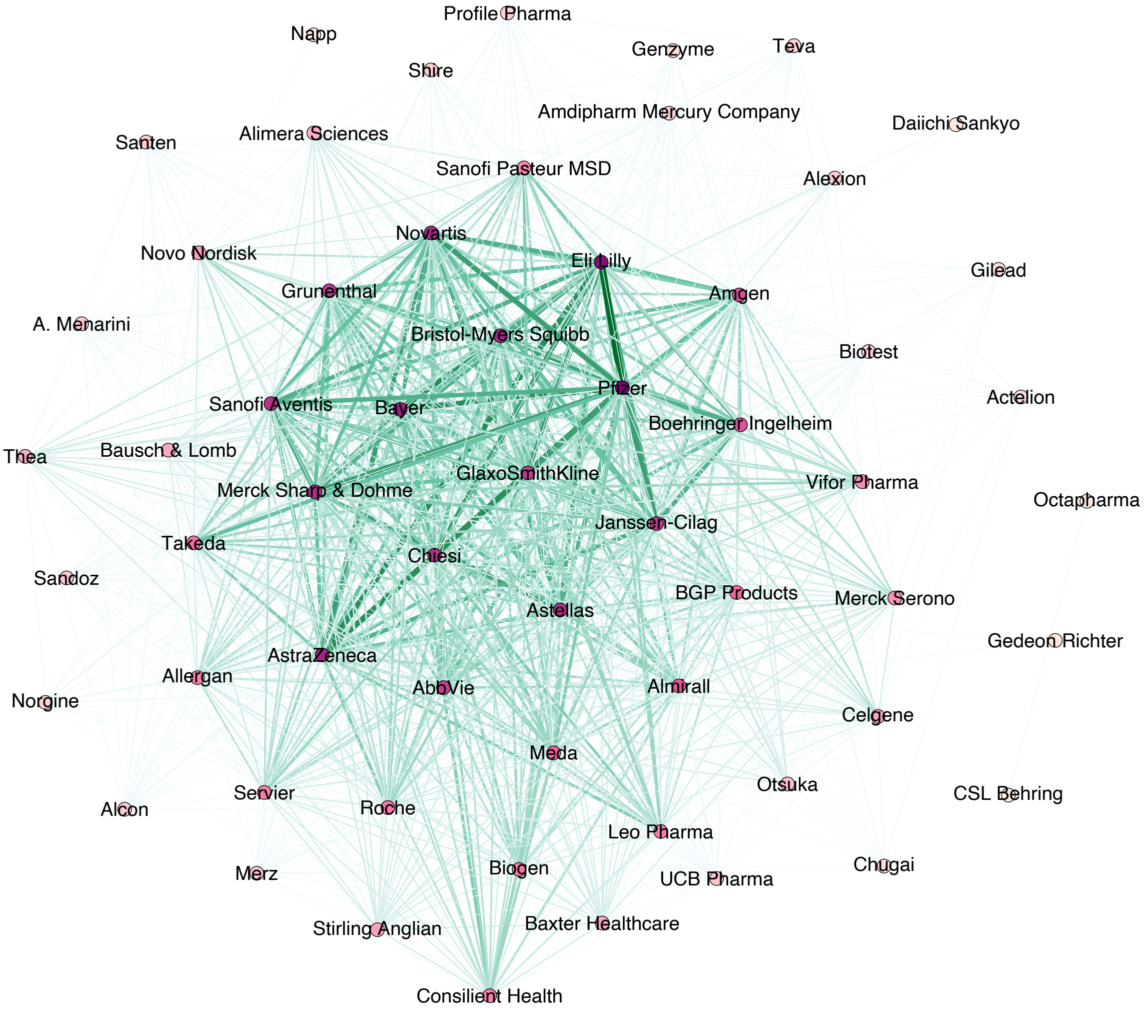

For peer review only - http://bmjopen.bmj.com/site/about/guidelines.xhtml

Supplementary File 6d. Northern Ireland's network

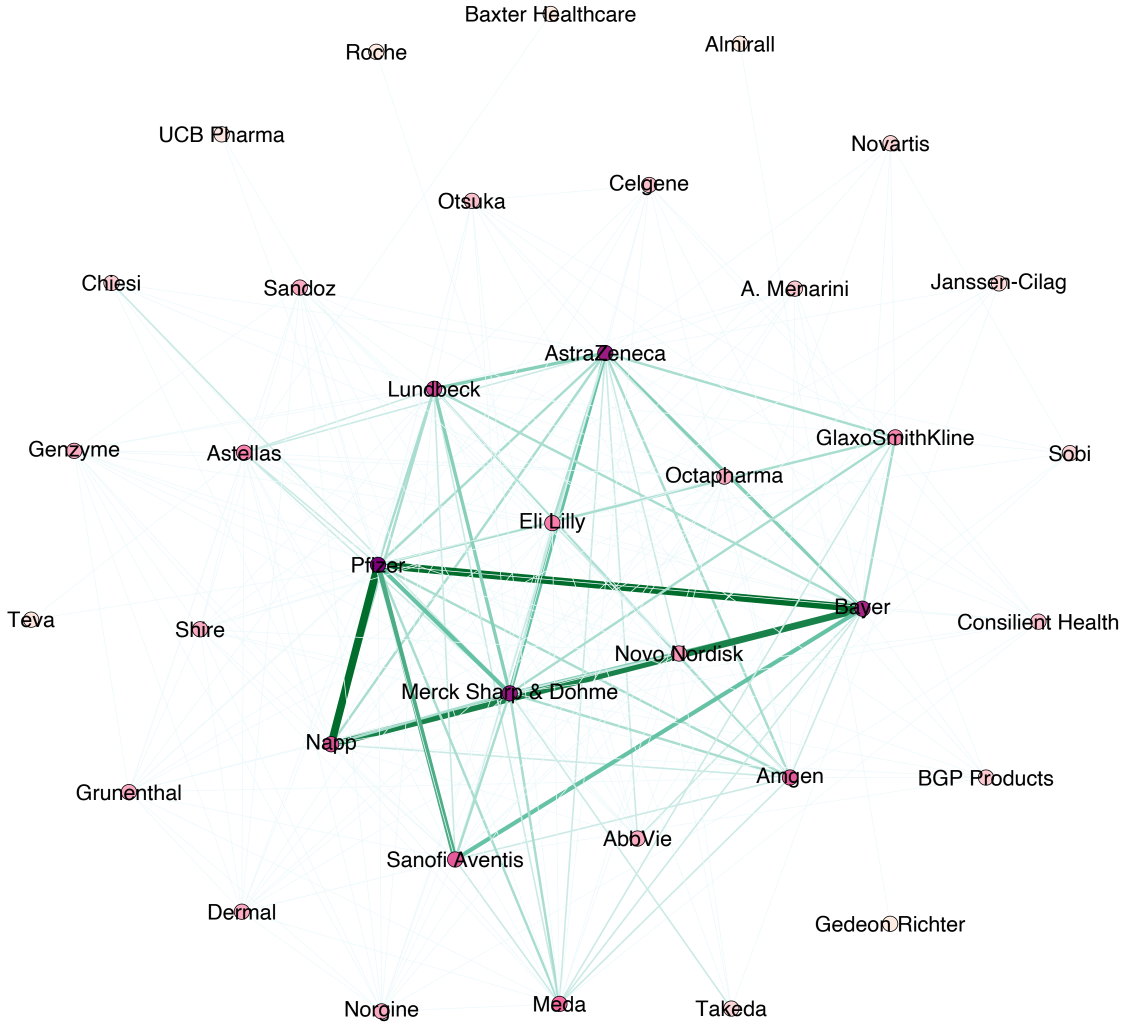

Note. All networks were visualised in Gephi v 0.9.2. Node label size and darkness corresponds to the weighted degree centrality of each company; the size and darkness of the edges (connecting lines) correspond to the number of shared recipients between companies

For peer review only - http://bmjopen.bmj.com/site/about/guidelines.xhtml

Supplementary File 7. Top ten companies by degree centrality scores in each country

England		Scotland		Wales		Northern Ireland	
Company	Degree	Company	Degree	Company	Degree	Company	Degree
Pfizer (2)	3394	Pfizer (2)	319	Pfizer (2)	206	Pfizer (2)	63
Merck Sharp & Dohme (6)	3064	Bayer (8)	260	Eli Lilly (3)	196	Merck Sharp & Dohme (6)	57
Bayer (8)	3060	Merck Sharp & Dohme (6)	251	Bayer (8)	176	AstraZeneca (1)	55
AstraZeneca (1)	2755	AstraZeneca (1)	245	AstraZeneca (1)	171	Bayer (8)	52
Eli Lilly (3)	2741	Eli Lilly (3)	245	Bristol-Myers Squibb (23)	169	Lundbeck (64)	43
Janssen-Cilag (10)	2539	GlaxoSmithKline (4)	241	Novartis (7)	168	Napp Pharmaceuticals (29)	39
GlaxoSmithKline (4)	2531	Janssen-Cilag (10)	227	Merck Sharp & Dohme (6)	161	Amgen (19)	36
Astellas Pharma (21)	2410	Sanofi Aventis (13)	214	Astellas Pharma (21)	160	Sanofi Aventis (13)	36
Chiesi (27)	2365	Astellas Pharma (21)	196	Chiesi (27)	158	Meda Pharma (46)	32
Sanofi Aventis (13)	2156	Bristol-Myers Squibb (23)	193	Sanofi Aventis (13)	156	Eli Lilly (3)	27

For peer review only

Supplementary File 8. Descriptive statistics for each recipient category

**England – categories**	**Value (%)**	**Payments – n (%)**	**Median - £ (IQR)**	**Recipients – n (%)**	**Drug companies**
Public sector secondary and tertiary care providers	**13,349,779.1 (25.56)**	**6660 (36.87)**	233.17 (141.87 – 500)	260 (7.41)	**89**
Patient organisation	12,227,843.2 (23.41)	1141 (6.32)	**4000 (500 – 11,104)**	288 (8.21)	65
Education and research providers	9,055,882.96 (17.34)	875 (4.84)	1000 (333.34 – 4,798.40)	56 (1.60)	68
Professional organisations	7,545,121.68 (14.44)	1776 (9.83)	500 (240 – 3,200)	354 (10.09)	84
Private companies other than providers of health services	3,975,461.63 (7.61)	1443 (7.99)	300 (196.8 – 598.92)	239 (6.81)	56
Public sector primary care providers	2,416,957.98 (4.63)	2513 (13.91)	434.5 (193.6 – 869)	**1809 (51.55)**	32
Private sector healthcare providers	1,322,785.04 (2.53)	463 (2.56)	240 (166 – 588)	108 (3.08)	44
Healthcare commissioning, planning and regulatory organisations	1,235,239.68 (2.36)	2166 (11.99)	208.17 (160 – 307.2)	206 (5.87)	47
Charities and other third-sector organisations	876,822.76 (1.68)	366 (2.03)	223.52 (157 – 487.2)	39 (1.11)	40
Formal bodies representing healthcare professionals or patients	121,351.93 (0.23)	458 (2.54)	200 (160 – 259.8)	68 (1.94)	25
Alternative providers of health services	93,534.91 (0.18)	180 (1.00)	200 (160 – 394)	62 (1.77)	28
Public administration and providers of public services	15,335.25 (0.03)	24 (0.13)	394.4 (224.45 – 546.67)	20 (0.57)	10
All payments	52,445,615.48	18065	280 (160 – 827.75)	3509	100
**Scotland - categories**	**Value (%)**	**Payments - n (%)**	**Median - £ (IQR)**	**Recipients - n (%)**	**Drug companies (%)**
Healthcare commissioning, planning and regulatory organisations	**878,333.57 (24.13)**	**582 (43.30)**	240 (131.18 - 500)	22 (8.30)	**53 (73.61)**
Private companies other than providers of health services	740,694.09 (20.35)	25 (1.86)	1,200 (350 - 5,528.88)	11 (4.15)	13 (18.06)
Education and research providers	708,149.16 (19.46)	141 (10.49)	1152 (400 – 2,880)	8 (3.02)	41 (56.94)
Patient organisation	620,384.33 (17.05)	52 (3.87)	1000 (253.68 - 9,745)	14 (5.28)	19 (26.39)
Professional organisations	466,833.11 (12.83)	291 (21.65)	450 (285.67 - 980)	64 (24.15)	52 (72.22)

Public sector primary care providers	112,308.91 (3.09)	128 (9.52)	434.5 (202.23 - 651,75)	**113 (42.64)**	13 (18.06)
Private sector healthcare providers	58,091.76 (1.60)	69 (5.13)	647.54 (206.65-1,500)	11 (4.15)	9 (12.50)
Public sector secondary and tertiary care providers	27,392.82 (0.75)	39 (2.90)	300 (189.75 - 612)	12 (4.53)	11 (15.28)
Charities and other third-sector organisations	19,710 (0.54)	4 (0.30)	**1700 (377.5 - 6,250)**	2 (0.75)	4 (5.56)
Alternative providers of health services	4,580 (0.13)	6 (0.45)	700 (360 – 1,100)	4 (1.51)	4 (5.56)
Public administration and providers of public services	2,700 (0.07)	5 (0.37)	540 (200 - 600)	3 (1.13)	5 (6.94)
Formal bodies representing healthcare professionals or patients	427.2 (0.01)	2 (0.15)	213.6 (211.4 - 214.8)	1 (0.38)	1 (1.39)
All payments	3,649,749.43	1,344	400 (180 - 864)	265	72

**Wales - categories**	**Value (%)**	**Payments - n (%)**	**Median - £ (IQR)**	**Recipients - n (%)**	**Drug companies (%)**
Healthcare commissioning, planning and regulatory organisations	**920,980.22 (46.38)**	**557 (56.61)**	225 (114.24 - 486.39)	10 (4.72)	**50 (78.13)**
Private companies other than providers of health services	179,495.4 (9.04)	56 (5.69)	**1475 (216 - 6,600)**	10 (4.72)	8 (12.50)
Education and research providers	179,256.38 (9.03)	37 (3.76)	336 (175.2 - 1000)	5 (2.36)	16 (25.00)
Public sector primary care providers	173,268.30 (8.73)	141 (14.33)	800 (434.5 - 1,152)	**118 (55.66)**	15 (23.44)
Private sector healthcare providers	153,983.36 (7.76)	18 (1.83)	440 (360.94 - 1,732)	6 (2.83)	9 (14.06)
Public administration and providers of public services	108,000 (5.44)	1 (0.10)	-	1 (0.47)	1 (1.56)
Patient organisation	99,784.32 (5.03)	22 (2.24)	747.93 (500 - 2,000)	10 (4.72)	11 (17.19)
Public sector secondary and tertiary care providers	96,862.66 (4.88)	20 (2.03)	253.66 (200 - 954)	3 (1.42)	13 (20.31)
Professional organisations	64,181.82 (3.23)	88 (8.94)	400 (280 - 800)	38 (17.92)	31 (48.44)
Charities and other third-sector organisations	5,036.8 (0.25)	17 (1.73)	120 (120 - 180)	4 (1.89)	7 (10.94)
Formal bodies representing healthcare professionals or patients	4,679.37 (0.24)	27 (2.74)	120 (96 - 142)	7 (3.30)	11 (17.19)
All payments	1,987,702.62	984	300 (144 - 800)	212	64

For peer review only

**Northern Ireland - categories**	**Value (%)**	**Payments - n (%)**	**Median - £ (IQR)**	**Recipients - n (%)**	**Drug companies (%)**
Public sector primary care providers	**184,903.09 (35.72)**	**127 (40.97)**	600 (434.5 - 1,600)	**94** (60.65)	6 (14.29)
Public sector secondary and tertiary care providers	111,743.45 (21.59)	83 (26.77)	288 (163.4 - 490.13)	5 (3.23)	**27 (64.29)**
Professional organisations	81,489.7 (15.74)	34 (10.97)	600 (320 - 1,784)	21 (13.55)	21 (50.00)
Patient organisation	43,205.6 (8.35)	15 (4.84)	650 (600 - 1,450)	7 (4.52)	14 (33.33)
Education and research providers	32,258 (6.23)	10 (3.23)	1100 (873.75 - 3525)	1 (0.65)	7 (16.67)
Private companies other than providers of health services	26,242.77 (5.07)	6 (1.94)	**4179.38 (1,152.19 - 7,687.5)**	4 (2.58)	3 (7.14)
Healthcare commissioning, planning and regulatory organisations	22,447.6 (4.34)	7 (2.26)	1500 (470.8 - 4,087)	4 (2.58)	6 (14.29)
Private sector healthcare providers	11,476.85 (2.22)	23 (7.42)	38.49 (28.9 - 485)	16 (10.32)	6 (14.29)
Formal bodies representing healthcare professionals or patients	2,133.34 (0.41)	2 (0.65)	1066.67 (933.33 - 1200.00)	1 (0.65)	1 (2.38)
Alternative providers of health services	1,700 (0.33)	3 (0.97)	600 (550 - 600)	2 (1.29)	2 (4.76)
All payments	517,600.40	310	475.2 (217.25 - 1,357.47)	155	42

For peer review only

Supplementary File 9. Top 10 recipients in each country

Country	Recipient	Category	Value - £	Payments - n	Companies - n
England	King's College London	Education and research providers	2,572,086.51	45	18
	Bladder and Bowel Foundation	Patient organisation	1,459,371.52	11	1
	London School Hyg and Tropical Med	Education and research providers	935,025.98	16	6
	PeerVoice	Private companies other than providers of health services	930,028.30	11	3
	University College London	Education and research providers	907,256.40	96	36
	Diabetes UK - England	Patient organisation	888,845.00	41	7
	Healthcare At Home	Private sector healthcare providers	872,740.81	18	2
	Cancer Research UK	Patient organisation	804,543.76	19	9
	Central Manchester Univ Hosps FT	Public sector secondary and tertiary care providers	739,595.97	108	37
	British Society for Rheumatology	Professional organisations	543,012.33	31	14
Scotland	Quintiles - Scotland	Private companies other than providers of health services	682,601.65	5	1
	Myeloma UK	Patient organisation	521,574.36	12	7
	NHS Greater Glasgow and Clyde	Healthcare commissioning, planning and regulatory organisations	483,354.99	153	34
	University of Glasgow	Education and research providers	442,707.63	70	26
	University of Dundee	Education and research providers	160,632.40	20	11
	NHS Lothian	Healthcare commissioning, planning and regulatory organisations	144,175.05	73	25
	University of Edinburgh	Education and research providers	73,014.39	37	21
	NHS Tayside	Healthcare commissioning, planning and regulatory organisations	67,924.08	74	24
	NHS Ayrshire and Arran	Healthcare commissioning, planning and regulatory organisations	63,276.93	48	25
	Digestive Disorders Federation	Professional organisations	60,796.00	2	2
Wales	Cardiff and Vale University HB	Healthcare commissioning, planning and regulatory organisations	344,131.95	89	28

For peer review only

	Abertawe Bro Morgannwg Univ HB	Healthcare commissioning, planning and regulatory organisations	242,418.82	124	32
	LloydsPharmacy	Private sector healthcare providers	146,376.00	4	1
	University of Cardiff	Education and research providers	120,822.78	27	13
	Bluebay Medical Systems	Private companies other than providers of health services	116,900.00	26	1
	Hywel Dda University HB	Healthcare commissioning, planning and regulatory organisations	115,600.62	77	27
	National Assembly for Wales	Public administration and providers of public services	108,000.00	1	1
	Betsi Cadwaladr University HB	Healthcare commissioning, planning and regulatory organisations	101,352.58	76	21
	Cwm Taf University Health Board	Healthcare commissioning, planning and regulatory organisations	84,624.15	119	25
	Velindre NHS Trust	Public sector secondary and tertiary care providers	80,629.32	18	13
Northern Ireland	Belfast Health and SC Trust	Public sector secondary and tertiary care providers	60,615.65	30	17
	Federation Of Family Practices	Public sector primary care providers	40,235.20	3	3
	UK and Ireland Society of Cataract and Refractive Surgeons - Northern Ireland	Professional organisations	35,000.00	1	1
	Queen's University Belfast	Education and research providers	32,258.00	10	7
	Northern Health and SC Trust	Public sector secondary and tertiary care providers	23,703.46	14	8
	Medical Communications	Private companies other than providers of health services	23,250.00	3	1
	Adult ADHD - Northern Ireland	Patient organisation	20,000.00	1	1
	Ulster Chemists' Association	Professional organisations	16,584.00	4	3
	Western Health and SC Trust	Public sector secondary and tertiary care providers	15,068.55	12	10
	Cancer Focus Northern Ireland	Patient organisation	12,255.60	2	2

For peer review only - http://bmjopen.bmj.com/site/about/guidelines.xhtml

Supplementary File 10. Post-hoc Bonferroni pairwise comparisons between countries of payments per category

Payment category	Group 1 – Group 2*	Test Statistic	Std. Error	Std. Test Statistic	Sig.	Adj. Sig†,‡	Median (IQR) – group 1 - £	Median (IQR) – group 2 - £
Alternative providers of health services	England-Scotland	-59.003	22.681	-2.601	0.009	**0.028**	200 (160 – 394)	700 (360 – 1,100)
	England-Northern Ireland	-65.919	31.816	-2.072	0.038	0.115	200 (160 – 394)	600 (550 - 600)
	Scotland-Northern Ireland	6.917	38.645	0.179	0.858	1	700 (360 – 1,100)	600 (550 - 600)
Charities and other third-sector organisations	England-Scotland	-118.214	56.132	-2.106	0.035	0.106	223.52 (157 – 487.2)	**1700 (377.5 - 6,250)**
	Wales-England	79.844	27.702	2.882	0.004	**0.012**	120 (120 - 180)	223.52 (157 – 487.2)
	Wales-Scotland	198.059	62.049	3.192	0.001	**0.004**	120 (120 - 180)	**1700 (377.5 - 6,250)**
Education and research providers	England-Scotland	-5.623	27.857	-0.202	0.84	1	1000 (333.34 – 4,798.40)	1152 (400 – 2,880)
	England-Northern Ireland	-92.61	97.628	-0.949	0.343	1	1000 (333.34 – 4,798.40)	1100 (873.75 - 3525)
	Scotland-Northern Ireland	86.987	100.458	0.866	0.387	1	1152 (400 – 2,880)	1100 (873.75 - 3525)
	Wales-England	204.193	51.523	3.963	<.001	**0**	336 (175.2 - 1000)	1000 (333.34 – 4,798.40)
	Wales-Scotland	209.816	56.703	3.7	<.001	**0.001**	336 (175.2 - 1000)	1152 (400 – 2,880)
	Wales-Northern Ireland	296.803	109.409	2.713	0.007	**0.04**	336 (175.2 - 1000)	1100 (873.75 - 3525)
Formal bodies representing healthcare professionals	England-Scotland	-46.978	99.854	-0.47	0.638	1	200 (160 – 259.8)	213.6 (211.4 - 214.8)
	England-Northern Ireland	-223.228	99.854	-2.236	0.025	0.152	200 (160 – 259.8)	1066.67 (933.33 - 1200.00)
	Scotland-Northern Ireland	176.25	140.907	1.251	0.211	1	213.6 (211.4 - 214.8)	1066.67 (933.33 - 1200.00)
	Wales-England	138.133	27.905	4.95	<.001	**0**	120 (96 - 142)	200 (160 – 259.8)
	Wales-Scotland	185.111	103.261	1.793	0.073	0.438	120 (96 - 142)	213.6 (211.4 - 214.8)

For peer review only - http://bmjopen.bmj.com/site/about/guidelines.xhtml

	Wales-Northern Ireland	361.361	103.261	3.5	<.001	**0.003**	120 (96 - 142)	1066.67 (933.33 - 1200.00)
Healthcare commissioning, planning and regulatory organisations	England-Scotland	-122.159	44.623	-2.738	0.006	**0.037**	208.17 (160 – 307.2)	240 (131.18 - 500)
	England-Northern Ireland	-786.684	361.817	-2.174	0.03	0.178	208.17 (160 – 307.2)	1500 (470.8 - 4,087)
	Scotland-Northern Ireland	664.525	363.4	1.829	0.067	0.405	240 (131.18 - 500)	1500 (470.8 - 4,087)
	Wales-Scotland	94.65	56.651	1.671	0.095	0.569	225 (114.24 - 486.39)	240 (131.18 - 500)
	Wales-Northern Ireland	759.175	363.497	2.089	0.037	0.22	225 (114.24 - 486.39)	1500 (470.8 - 4,087)
	England-Wales	-27.51	45.405	-0.606	0.545	1	208.17 (160 – 307.2)	225 (114.24 - 486.39)
Patient organisation	Wales-England	144.727	77.321	1.872	0.061	0.367	747.93 (500 - 2,000)	**4000 (500 – 11,104)**
	Wales-Scotland	29.422	91.028	0.323	0.747	1	747.93 (500 - 2,000)	1000 (253.68 - 9,745)
	Northern Ireland-Wales	-8.633	118.686	-0.073	0.942	1	650 (600 - 1,450)	747.93 (500 - 2,000)
	Northern Ireland-Scotland	-38.055	103.121	-0.369	0.712	1	650 (600 - 1,450)	1000 (253.68 - 9,745)
	Northern Ireland-England	153.361	91.248	1.681	0.093	0.557	650 (600 - 1,450)	**4000 (500 – 11,104)**
	Scotland-England	115.306	50.259	2.294	0.022	0.131	1000 (253.68 - 9,745)	**4000 (500 – 11,104)**
Private companies other than providers of health services	England-Scotland	-317.041	89.112	-3.558	<.001	**0.002**	300 (196.8 – 598.92)	1,200 (350 - 5,528.88)
	England-Northern Ireland	-527.308	180.717	-2.918	0.004	**0.021**	300 (196.8 – 598.92)	**4179.38 (1,152.19 - 7,687.5)**
	Scotland-Northern Ireland	210.267	200.821	1.047	0.295	1	1,200 (350 - 5,528.88)	**4179.38 (1,152.19 - 7,687.5)**
	Wales-Scotland	115.061	106.256	1.083	0.279	1	**1475 (216 - 6,600)**	1,200 (350 - 5,528.88)
	Wales-Northern Ireland	325.327	189.758	1.714	0.086	0.519	**1475 (216 - 6,600)**	**4179.38 (1,152.19 - 7,687.5)**

For peer review only - http://bmjopen.bmj.com/site/about/guidelines.xhtml

	England-Wales	-201.98	60.166	-3.357	<.001	**0.005**	300 (196.8 – 598.92)	**1475 (216 - 6,600)**
Private sector healthcare providers	England-Scotland	-64.982	21.355	-3.043	0.002	**0.014**	240 (166 – 588)	647.54 (206.65 - 1,500)
	England-Wales	-88.107	39.755	-2.216	0.027	0.16	240 (166 – 588)	440 (360.94 - 1,732)
	Northern Ireland-Wales	-206.778	52.076	-3.971	<.001	**0**	38.49 (28.9 - 485)	440 (360.94 - 1,732)
	Northern Ireland-Scotland	-183.652	39.843	-4.609	<.001	**0**	38.49 (28.9 - 485)	647.54 (206.65 - 1,500)
	Northern Ireland-England	118.671	35.352	3.357	<.001	**0.005**	38.49 (28.9 - 485)	240 (166 – 588)
	Scotland-wales	-23.126	43.797	-0.528	0.597	1	647.54 (206.65 - 1,500)	440 (360.94 - 1,732)
Professional organisations	England-Northern Ireland	-35.169	109.4	-0.321	0.748	1	500 (240 – 3,200)	600 (320 - 1,784)
	Scotland-Northern Ireland	157.615	114.524	1.376	0.169	1	450 (285.67 - 980)	600 (320 - 1,784)
	Wales-England	182.447	69.008	2.644	0.008	**0.049**	400 (280 - 800)	500 (240 – 3,200)
	Wales-Scotland	60.001	76.873	0.781	0.435	1	400 (280 - 800)	450 (285.67 - 980)
	Wales-Northern Ireland	217.616	127.597	1.705	0.088	0.529	400 (280 - 800)	600 (320 - 1,784)
	Scotland-England	122.446	39.962	3.064	0.002	**0.013**	450 (285.67 - 980)	500 (240 – 3,200)
Public sector primary care providers	England-Northern Ireland	-355.779	76.292	-4.663	<.001	**0**	434.5 (193.6 – 869)	600 (434.5 - 1,600)
	Scotland-Northern Ireland	407.76	105.06	3.881	<.001	**0.001**	434.5 (202.23 - 651,75)	600 (434.5 - 1,600)
	England-Wales	-459.79	72.597	-6.333	<.001	**0**	434.5 (193.6 – 869)	800 (434.5 - 1,152)
	Northern Ireland-Wales	-104.011	102.62	-1.014	0.311	1	600 (434.5 - 1,600)	800 (434.5 - 1,152)
	Scotland-England	51.98	76.008	0.684	0.494	1	434.5 (202.23 - 651,75)	434.5 (193.6 – 869)
	Scotland-wales	-511.771	102.409	-4.997	<.001	**0**	434.5 (202.23 - 651,75)	800 (434.5 - 1,152)

*Each row tests the null hypothesis that the Sample 1 and Sample 2 distributions are the same.
†Asymptotic significances (2-sided tests) are displayed. The significance level is .050.
‡Significance values have been adjusted by the Bonferroni correction for multiple tests

For peer review only - http://bmjopen.bmj.com/site/about/guidelines.xhtml

Supplementary File 11. Post-hoc Bonferroni pairwise comparisons between countries of payment types

Payment Type	Group 1 – Group 2*	Test Statistic	Std. Error	Std. Test Statistic	Sig.	Adj. Sig†,‡	Median (IQR) – group 1 - £	Median (IQR) – group 2 - £
Contribution to costs of Events	Wales-England	498.254	159.743	3.119	0.002	0.011	223.36 (120 - 400)	240 (155.95 - 400)
	Wales-Scotland	1449.82	208.45	6.955	<.001	0	223.36 (120 - 400)	300 (160 - 600)
	Wales-Northern	2690.485	402.839	6.679	<.001	0	223.36 (120 - 400)	477.6 (200 - 1,147.6)
	England-Scotland	-951.566	143.605	-6.626	<.001	0	240 (155.95 - 400)	300 (160 - 600)
	England-Northern	-2192.23	373.43	-5.871	<.001	0	240 (155.95 - 400)	477.6 (200 - 1,147.6)
	Scotland-Northern	1240.664	396.716	3.127	0.002	0.011	300 (160 - 600)	477.6 (200 - 1,147.6)
Donations and Grants	Wales-England	25.151	94.77	0.265	0.791	1	800 (434.5 - 2,200)	959.98 (256 - 4,800)
	Northern-Scotland	-140.935	137.745	-1.023	0.306	1	434.5 (217.5 - 1,867.5)	651.75 (217.25 - 2,578)
	Northern-Wales	-451.347	142.863	-3.159	0.002	0.009	434.5 (217.5 - 1,867.5)	800 (434.5 - 2,200)
	Northern-England	476.498	111.315	4.281	<.001	0	434.5 (217.5 - 1,867.5)	959.98 (256 - 4,800)
	Scotland-Wales	-310.412	124.755	-2.488	0.013	0.077	651.75 (217.25 - 2,578)	800 (434.5 - 2,200)
	Scotland-England	335.563	86.862	3.863	<.001	0.001	651.75 (217.25 - 2,578)	959.98 (256 - 4,800)

*Each row tests the null hypothesis that the Sample 1 and Sample 2 distributions are the same.
†Asymptotic significances (2-sided tests) are displayed. The significance level is .050.
‡Significance values have been adjusted by the Bonferroni correction for multiple tests.

For peer review only - http://bmjopen.bmj.com/site/about/guidelines.xhtml

STROBE Statement—Checklist of items that should be included in reports of ***cross-sectional studies***

	Item No	Recommendation	Page No
**Title and abstract**	1	(*a*) Indicate the study's design with a commonly used term in the title or the abstract	2
		(*b*) Provide in the abstract an informative and balanced summary of what was done and what was found	2
**Introduction**			
Background/rationale	2	Explain the scientific background and rationale for the investigation being reported	3-4
Objectives	3	State specific objectives, including any prespecified hypotheses	4
**Methods**			
Study design	4	Present key elements of study design early in the paper	5
Setting	5	Describe the setting, locations, and relevant dates, including periods of recruitment, exposure, follow-up, and data collection	5
Participants	6	(*a*) Give the eligibility criteria, and the sources and methods of selection of participants	5
Variables	7	Clearly define all outcomes, exposures, predictors, potential confounders, and effect modifiers. Give diagnostic criteria, if applicable	5-6
Data sources/ measurement	8*	For each variable of interest, give sources of data and details of methods of assessment (measurement). Describe comparability of assessment methods if there is more than one group	5-6
Bias	9	Describe any efforts to address potential sources of bias	n/a
Study size	10	Explain how the study size was arrived at	N/a
Quantitative variables	11	Explain how quantitative variables were handled in the analyses. If applicable, describe which groupings were chosen and why	5
Statistical methods	12	(*a*) Describe all statistical methods, including those used to control for confounding	5
		(*b*) Describe any methods used to examine subgroups and interactions	n/a
		(*c*) Explain how missing data were addressed	n/a
		(*d*) If applicable, describe analytical methods taking account of sampling strategy	n/a
		(*e*) Describe any sensitivity analyses	n/a
**Results**			
Participants	13*	(a) Report numbers of individuals at each stage of study—eg numbers potentially eligible, examined for eligibility, confirmed eligible, included in the study, completing follow-up, and analysed	Supplementary File 4 (flow diagram)
		(b) Give reasons for non-participation at each stage	n/a
		(c) Consider use of a flow diagram	Supplementary File 4

For peer review only - http://bmjopen.bmj.com/site/about/guidelines.xhtml

Descriptive data	14*	(a) Give characteristics of study participants (eg demographic, clinical, social) and information on exposures and potential confounders	n/a
		(b) Indicate number of participants with missing data for each variable of interest	n/a
Outcome data	15*	Report numbers of outcome events or summary measures	6
Main results	16	(*a*) Give unadjusted estimates and, if applicable, confounder-adjusted estimates and their precision (eg, 95% confidence interval). Make clear which confounders were adjusted for and why they were included	n/a
		(*b*) Report category boundaries when continuous variables were categorized	n/a
		(*c*) If relevant, consider translating estimates of relative risk into absolute risk for a meaningful time period	n/a
Other analyses	17	Report other analyses done—eg analyses of subgroups and interactions, and sensitivity analyses	n/a
**Discussion**			
Key results	18	Summarise key results with reference to study objectives	11-13
Limitations	19	Discuss limitations of the study, taking into account sources of potential bias or imprecision. Discuss both direction and magnitude of any potential bias	13
Interpretation	20	Give a cautious overall interpretation of results considering objectives, limitations, multiplicity of analyses, results from similar studies, and other relevant evidence	11-14
Generalisability	21	Discuss the generalisability (external validity) of the study results	11-14
**Other information**			
Funding	22	Give the source of funding and the role of the funders for the present study and, if applicable, for the original study on which the present article is based	1

*Give information separately for exposed and unexposed groups.

Note: An Explanation and Elaboration article discusses each checklist item and gives methodological background and published examples of transparent reporting. The STROBE checklist is best used in conjunction with this article (freely available on the Web sites of PLoS Medicine at http://www.plosmedicine.org/, Annals of Internal Medicine at http://www.annals.org/, and Epidemiology at http://www.epidem.com/). Information on the STROBE Initiative is available at www.strobe-statement.org.

For peer review only

For peer review only - http://bmjopen.bmj.com/site/about/guidelines.xhtml

BMJ Open

Comparing pharmaceutical company payments in the four UK countries: a cross-sectional and social network analysis

Journal:	*BMJ Open*
Manuscript ID	bmjopen-2022-061591.R1
Article Type:	Original research
Date Submitted by the Author:	16-Jan-2023
Complete List of Authors:	Rickard, Emily; University of Bath, Department of Social and Policy Sciences<br>Carmel, Emma; University of Bath, Department of Social and Policy Sciences<br>Ozieranski, Piotr; University of Bath, Department of Social and Policy Sciences
**Primary Subject Heading**:	Health policy
Secondary Subject Heading:	Public health
Keywords:	Health policy < HEALTH SERVICES ADMINISTRATION & MANAGEMENT, MEDICAL ETHICS, PUBLIC HEALTH

SCHOLARONE™
Manuscripts

I, the Submitting Author has the right to grant and does grant on behalf of all authors of the Work (as defined in the below author licence), an exclusive licence and/or a non-exclusive licence for contributions from authors who are: i) UK Crown employees; ii) where BMJ has agreed a CC-BY licence shall apply, and/or iii) in accordance with the terms applicable for US Federal Government officers or employees acting as part of their official duties; on a worldwide, perpetual, irrevocable, royalty-free basis to BMJ Publishing Group Ltd ("BMJ") its licensees and where the relevant Journal is co-owned by BMJ to the co-owners of the Journal, to publish the Work in this journal and any other BMJ products and to exploit all rights, as set out in our licence.

The Submitting Author accepts and understands that any supply made under these terms is made by BMJ to the Submitting Author unless you are acting as an employee on behalf of your employer or a postgraduate student of an affiliated institution which is paying any applicable article publishing charge ("APC") for Open Access articles. Where the Submitting Author wishes to make the Work available on an Open Access basis (and intends to pay the relevant APC), the terms of reuse of such Open Access shall be governed by a Creative Commons licence – details of these licences and which Creative Commons licence will apply to this Work are set out in our licence referred to above.

Other than as permitted in any relevant BMJ Author's Self Archiving Policies, I confirm this Work has not been accepted for publication elsewhere, is not being considered for publication elsewhere and does not duplicate material already published. I confirm all authors consent to publication of this Work and authorise the granting of this licence.

For peer review only

Comparing pharmaceutical company payments in the four UK countries: a cross-sectional and social network analysis

Emily Rickard
Department of Social and Policy Sciences, University of Bath, Bath, United Kingdom.
Email: e.j.rickard@bath.ac.uk

Emma Carmel
Department of Social and Policy Sciences, University of Bath, Bath, United Kingdom.
Email: e.k.carmel@bath.ac.uk

Piotr Ozieranski (Corresponding Author)
Department of Social and Policy Sciences, University of Bath, Bath, United Kingdom.
Email: p.ozieranski@bath.ac.uk

Word count: 4921

Key words
Transparency, financial disclosures, pharmaceutical industry, health policy, conflicts of interest

ABSTRACT

Objectives To examine the characteristics of pharmaceutical payments to healthcare and patient organisations in the four UK countries. Compare companies spending the most; types of organisations; and types of payments in the four countries. Measure the extent to which companies target payments at the same recipients in each country and whether it differs depending on the type of recipient.

Design Cross-sectional comparative and social network analysis.

Setting England, Scotland, Wales, Northern Ireland.

Participants 100 donors (pharmaceutical companies) reporting payments to 4,229 recipients (healthcare organisations and patient organisations) in 2015.

Main outcome measures Number of payments incorrectly reported in Disclosure UK and payments reported twice in two places –Disclosure UK and company websites. For each country: payment totals and distribution; average number of common recipients between companies; share of payments to organisations fulfilling different roles in the health ecosystem; and payments for different activities.

Results We found evidence of reporting errors in Disclosure UK. Companies prioritised different types of recipient and different types of activity in each country. There were significant differences in the distribution of payments across the four countries, even for

For peer review only - http://bmjopen.bmj.com/site/about/guidelines.xhtml

similar types of recipients. Recipients in England and Wales received smaller individual payments than in Scotland and Northern Ireland. Overall, targeting shared recipients occurred most frequently in England, but was also common in certain pockets of each country's health ecosystem.

Conclusions Our findings suggest a strategic approach to payments tailored to countries' policy and decision-making context, indicating there may be specific vulnerabilities to potential financial conflicts of interest at sub-national level. Payment differences between countries may be occurring in other countries, particularly those with decentralised health systems and/or high levels of independence across its decision-making authorities. We call for a single database containing all recipient types, full location details, and published with associated descriptive and network statistics.

For peer review only

STRENGTHS AND LIMITATIONS OF THIS STUDY

- This is the first study to compare pharmaceutical industry payments in England, Scotland, Wales and Northern Ireland
- Our analysis created a new database combining payments disclosed in Disclosure UK with individual company disclosures of payments to patient organisations
- We use social network analysis to facilitate a systematic sub-national comparison of payments
- One key limitation is that the data is from 2015 and is not able to assess trends in payment types or amounts.

INTRODUCTION

Some of the major pharmaceutical companies spend more on marketing than on the development of products[1 2 3]. Industry marketing efforts include payments to physicians, which are seen to boost innovation and efficiency in healthcare[4] but also generate concerns about potential *individual* financial conflicts of interest (COIs), influencing prescribing choices[5] and leading to patient harms[6]. Payments to healthcare and patient organisations have also been seen to generate potential *institutional* financial COIs around policy and programme decision-making. An institution's primary goals *may* be unduly influenced by a secondary interest[7], which can be more damaging than individual COIs[7-9]. COIs are defined in terms of the *risk* of undue influence and not actual bias or misconduct[9], but institutional COIs have been linked to increased prescriptions of drugs with unproven safety[8], distorting research agendas[10], threatening the objectivity of professional education[7], and compromising independence[11]. These observations have highlighted what has been called the pharmaceutical industry's 'web of influence', in which companies "sustain large networks to gather, create, control and disseminate information"[12].

The potential to distort public health research and policy to favour commercial interests above patients' has led to increased policy scrutiny[13 14], including the introduction of self-regulatory payment disclosure requirements for pharmaceutical companies in Europe[15]. Such measures are intended to aid transparency, reducing potential conflicts of interest and undue influence on clinical and policy decisions. This article combines and analyses disclosure data to better understand the depth, breadth, and structure of industry payments and compares them in the four countries of the UK. Comparative analysis can illustrate novel ethical and governance problems[16] or reveal that recognised problems are common across countries[17], which our systematic examination of the extent and diversity of payments reported by pharmaceutical companies explores.

Disclosure of industry payments

In the United States, pharmaceutical industry disclosures of payments to physicians and teaching hospitals were made mandatory in 2013[18], and subsequent research has examined payments to physicians[19-30], with institutional payments to hospitals largely ignored[31]. Payments to patient organisations, defined as not-for-profit institutions that primarily represent the needs of patients and/or caregivers[32], have been seldom explored in the US[33 34] as their disclosure is not regulated by the state or industry.

The prevalence of self-regulation in Europe is associated with very different disclosure rules to the US[35 36]. Since 2012, the European trade association, the European Federation of Pharmaceutical Industries and Associations (EFPIA), has mandated that pharmaceutical companies publish annual disclosures of their payments to patient organisations on their websites[37]. Subsequent studies revealed extensive funding in the UK[38 39] and the Nordic countries[40 41]. However, transparency remains limited by a lack of standardised reporting requirements and limited oversight[42] which are associated with payment under-reporting by both donors and recipients[39].

In separate self-regulatory arrangements[43], disclosures of payments to healthcare organisations, defined by the industry as healthcare, medical or scientific associations or organisations such as hospitals, clinics, foundations or universities[44], have been mandated

For peer review only - http://bmjopen.bmj.com/site/about/guidelines.xhtml

since 2015. In the UK, these are reported annually in a centralised database, Disclosure UK, hosted by the industry trade body, the Association for the British Pharmaceutical Industry (ABPI). Most research attention has been on the poor accessibility and quality of the data[35], noting lack of standardisation of recipients[4 25] and inadequate details about individual payments' purpose[4]. These make tracking and analysing the payments complicated and time-consuming, hindering the principle aim of improving transparency.

Our study is the first, to our knowledge, to systematically combine industry data on payments to healthcare organisations and patient organisations as the self-regulatory codes allow them to be reported separately. Analysing them together enables us to better assess the breadth and depth of the 'web of influence', and gain insight into potential reinforcement effects of payments to multiple and diverse organisations that have separate yet overlapping interests, including providing patient care and support[31 38], involvement in policy-making[13 45 46], and conducting clinical research[9 38].

Regional differences in industry payments

Another aspect of the industry's web of influence largely unexplored in Europe is whether and how it is structured around regional differences in payments. Little is known about strategic targeting of particular fields of healthcare provision and/or decision-making, nor about possible effects on potential COIs in regional policy-making. Regionally targeted payments may have direct policy effects 'upstream', such as commissioning (the planning, prioritising, and purchasing of public health services)[47]; and 'downstream', such as bearing greater influence on organisational priorities and day-to-day practices.

Emerging US research has found significant differences in the distribution of payments between states[20 48-50], including by state size[51] and political composition[52], indicating that demographics and the organisation and regulation of healthcare matter. The first regional analysis in Europe revealed differences in the total value and type of payments prioritised in eight countries[17]. Most recently a UK study found headquarter distance from country capitals predicts patient organisations' dependence on pharmaceutical company funding[53]. To date, research has not considered the locations of patient and healthcare organisations as the reporting requirements do not extend to disclosing country locations[17 25 28].

However, the UK presents a crucial case for this type of analysis given its importance as a pharmaceutical market[54], large value of payments compared to other European countries[17] and vast charitable sector comprising many potential recipients[55]. England, Scotland, Wales and Northern Ireland have four distinct health systems, with substantial autonomy to determine health policies and services[56-58]. They also differ demographically – population size is largest in England and smallest in Northern Ireland[59] and health outcomes are highest in England and lowest in Scotland[60]. The demographic and health system differences could be associated with how industry engages with different healthcare sectors.

We know that pharmaceutical companies prioritise payments to different types of healthcare organisations in the UK[25 28], however commercially patterned inequalities, including dominant funders or types of recipients, may be more pronounced sub-nationally in the in the smallest UK countries yet hidden by UK-level analysis to date[16]. Studies have started recognising the country distinction, focusing on payments to healthcare

For peer review only - http://bmjopen.bmj.com/site/about/guidelines.xhtml

organisations in England[47 61], but cross-country comparisons have not yet been conducted. Comparative insights could also help understand whether similar patterns are occurring in other European countries with highly decentralised healthcare set-ups, including Germany[62] and Spain[63].

In this article, we apply social network analysis (SNA) which offers new insights into industry marketing tactics[64 65 61]. SNA can reveal areas of the healthcare ecosystem where connections between companies, measured by the number of payment recipients companies have in common, are most prevalent. Prevalent connections may highlight industry marketing efforts in pockets of each of the UK's health systems, including indicating areas of *competition* between companies[66 67 68] and revealing areas where companies are seeking to enhance their visibility[61 69].

We integrate and analyse data from Disclosure UK and disclosures of payments to patient organisations to examine patterns in pharmaceutical company payments to organisations in the UK healthcare ecosystem. Specifically, our objectives are to:

- examine the characteristics of payments to healthcare and patient organisations in the four countries
- compare the top donors financially in each country
- identify similarities and differences in the types of payments and in the types of organisations receiving payments in the four countries
- measure the extent to which companies target payments at the same recipients in each country and whether it differs depending on the type of recipient.

METHODS

Data sources

Our primary data sources are publicly available pharmaceutical industry transparency disclosures from 2015. Corresponding to relevant ABPI[70] and EFPIA Codes[37], pharmaceutical companies disclose payments to healthcare organisations and to patient organisations separately.

Payments to healthcare organisations are disclosed in a centralised database, Disclosure UK, published annually by the ABPI. Payments are disclosed with recipient name, payment type (donations and grants, costs of events, joint working, and consultancy – see Supplementary File 1) and value, and address information. We use the 2015 version of Disclosure UK and focus on non-R&D payments to healthcare organisations (R&D payments are reported as one lump sum per company without named recipients[25 43]).

Payments to patient organisations are only available on individual pharmaceutical company websites and are usually presented as a PDF file and include recipient name, payment description, and payment value[42]. We extracted the payments to patient organisations data into a single database, standardising names and identifying headquarter addresses as part of another project[38]. We detail our approach to data cleaning these data elsewhere[25 38].

For peer review only - http://bmjopen.bmj.com/site/about/guidelines.xhtml

Dataset preparation and integration

We followed several steps to prepare the Disclosure UK and patient organisation datasets for analysis. First, we merged the two datasets (see Supplementary File 2 for data integration flowchart). Second, as Disclosure UK provides incomplete addresses, we conducted independent web searches on each payment recipient to determine which UK country they are based. We used the same methodology to determine patient organisations' locations. Third, we excluded payments to patient organisations duplicated in the two datasets and identified patient organisations incorrectly reported as healthcare organisations in Disclosure UK. Fourth, we coded the patient organisation descriptions to match the codes used by Disclosure UK (Supplementary File 1).

Fifth, as part of a previous study[25] we standardised recipient names for almost 20,000 payment entries and inductively categorised them based on their function within the healthcare system (e.g. service provider) and their sector (e.g. public or private) (see Supplementary File 3 for comprehensive definitions and examples of organisations). For the current study we introduced patient organisations. Recipient types (with the most frequently occurring example) included in our analysis are:

Providers of health services

- Alternative providers of health services (e.g. community interest companies providing health services)
- Healthcare commissioning, planning and regulatory organisations (e.g. clinical commissioning groups)
- Private sector healthcare providers (e.g. private healthcare groups)
- Public sector primary care providers (e.g. GP surgeries)
- Public sector secondary and tertiary care providers (e.g. NHS trusts)

Representative organisations

- Formal bodies representing healthcare professionals or patients (e.g. local medical committees)
- Patient organisations (e.g. multipurpose patient organisations)
- Professional organisations (e.g. multi-professional or multi-stakeholder organisations)

Other organisations

- Charities and other third-sector organisations (excludes providers of health services, professional organisations, and patient organisations) (e.g. charitable trusts providing educational events for healthcare professionals)
- Education and research providers (e.g. universities)
- Private companies other than providers of health services (e.g. providers of medical communications or training services)
- Public administration and providers of public services (e.g. local authorities)
- Recipients unclear (when no information could be found)

Analysis

We calculated the total and median value of payments in each country and recipient type. The Shapiro-Wilks test of normalcy found the data to be non-normal in each country,

For peer review only - http://bmjopen.bmj.com/site/about/guidelines.xhtml

therefore non-parametric Kruskal-Wallis tests (adjusted for ties) were used to check for between-country differences in the distribution of payments overall and in the different recipient types. Dunn's post-hoc pairwise analyses (with Bonferroni's correction for multiple comparisons) were conducted to identify where differences were present between countries and recipient types. Kruskal-Wallis and Dunn's tests do not assume equal sample sizes[71] and have been conducted on similar industry disclosure data[72 73 74]. Statistical significance was set at $p = \leq .05$.

SNA was used to measure the number payment recipients that were common between pairs of pharmaceutical companies (density) and across all companies (degree centrality). Density measures the overall level of connection in a network and can be used to compare the structure of different groups[75]. It produces two outputs – average value (average number of recipients each pair of companies shares[76]) and average weighted degree (average of the total number recipients each company shares with other companies). The higher these values, the more frequently a multiple companies target the same recipients in a given network[77]. For example, a density score of 1.194 tells us that all pairs of companies in the network funded an average of 1.2 recipients in common. Degree centrality, on the other hand, provides a score for each individual company based on the number of recipients in common it shares with other companies in the network – the higher the score, the more recipients a company shares[75 78]. For example, if a company has a degree centrality score of 320, they funded the same recipient as another company 320 times.

We compare the number of common recipients companies have in each country overall and when targeting different recipient types in each country. SNA requires data to be structured as a matrix, therefore we transformed the payment data into a series of matrices of pharmaceutical companies with ties based on the number of recipients each company shared with other companies in each country and recipient type. To identify which companies targeted the same recipients, each matrix consisted only of the companies making at least one payment (regardless of whether or not they shared any recipients). We conducted separate network analyses on each of the four countries as the findings would otherwise be highly influenced by England's data as the largest network.

Data was processed in Microsoft Excel. The dataset underpinning our analysis is published in the Bath Research Data Archive[79]. We analysed the data descriptively in SPSS version 27 (IBM) and Microsoft Excel. We conducted social network analysis in UCINET version 6[77]. Country networks were visualised in Gephi version 0.9.2.

Outcome measures

Disclosure accuracy

First, we measured the number of patient organisations, alongside the number and value of payments, that were incorrectly disclosed as healthcare organisations in Disclosure UK.

Breadth of payments in each country

Second, we explored the payment characteristics in each country. We measured the total and median values and the number of: payments, recipients, and companies. We adjusted

For peer review only - http://bmjopen.bmj.com/site/about/guidelines.xhtml

the total value by population size for comparison. We also compared the distribution of payments between each country using Kruskal-Wallis tests.

Third, we identified the top 10% of companies making payments in each country and compared the payment strategies of the companies paying the most in each country.

Depth of payments in each country

Fourth, we assessed companies making payments to the same recipients by measuring the average number of common recipients between each pair of companies (degree centrality).

Fifth, we scrutinised which companies dominate the payment networks in each country by identifying the number of recipients that each company had in common with every other company.

Structure of payments in each country

Sixth, for each country we identified which type of recipient was prioritised. To do this, we measured and compared the proportion of payments received by each recipient type. We also compared the distribution of payments to each recipient type using Kruskal-Wallis tests to determine whether payments to similar types of recipients differ between countries.

Seventh, we examined whether companies making payments to *each recipient type in each country* made payments to the same organisations by measuring the average number of recipients each pair of companies share.

Finally, for each country we assessed which types of payments were prioritised through identifying the proportion of different payment types. We also compared the four types of payments using Kruskal-Wallis tests to identify differences in the distribution of payments.

Patient and public involvement

The study did not involve patients.

RESULTS

We structure our findings consistent with the order of the outcome measures outlined above. First, we examine overall accuracy of disclosures. Next, we explore the breadth, depth, and structure of payments in each country. While there is inevitable overlap between these framing terms, this will be signposted throughout.

Accuracy of disclosures

We found evidence of pharmaceutical companies misinterpreting disclosure requirements when we integrated the Disclosure UK and patient organisation data (see Supplementary File 3 for data integration flowchart). We identified 341 payments (1.71% of all payments to organisations in Disclosure UK) to 116 patient organisations (2.88% of all organisations in Disclosure UK) worth £2,458,931.99 (5.21% of the total) incorrectly disclosed as healthcare organisations in Disclosure UK. Of these payments, 50 (14.66%) were duplicated in the patient organisation and Disclosure UK data, which were excluded to ensure no payment was counted twice.

For peer review only

Breadth of payments in the four countries
The total value and number of payments, the number of recipients, and the number of companies making payments were consistent with the size of each country, with England receiving the highest and Northern Ireland the lowest (this was maintained after adjusting for population size – see Table 1).

Table 1. Value and number of payments, number of companies and recipients, and top donors in integrated dataset

Descriptive statistic	England	Scotland	Wales	Northern Ireland
Country population 2015 - n*	54,786,300	5,373,000	3,099,100	1,851,600
Total value - £	52,445,615	3,649,749	1,987,703	518,000
Total value - £ (adjusted for population size)†	957,037	675,880	641,194	272,632
Payments - n	18,190	1,370	990	311
Recipients - n	3,575	282	216	156
Companies – n	100	72	64	42
Median payment value (IQR) - £	280 (665.5)	400 (685.3)	300 (658.2)	475.20 (1,164.4)
Value of payments to healthcare organisations - £	40,217,772	3,029,365	1,887,918	474,795
Value of payments to patient organisations - £	12,227,843	620,384	99,784	43,206

*Data obtained from the Office for National Statistics, values correct for mid-2015
†Total value of payments divided by the population size

Between-country differences in payment values
There was a statistically significant difference in the distribution of individual payments between the four countries, $\chi2(3) = 50.127$, p = <.001. Dunn's post-hoc comparisons showed that this difference was driven by significantly higher median payments (Table 1) being made in Scotland (p - <.001) and Northern Ireland (p = <.001) than England. Payment size also varied significantly between Northern Ireland-Wales (p = <.000), Scotland-Wales (p = .001), and Northern Ireland-Scotland (p = .004).

Top donors in each country
The companies spending most in each country also reveals different approaches to payments (see Supplementary File 4). The top donors generally made larger payments in Wales and multiple smaller payments in Northern Ireland. Pfizer was consistently a top donor measured by value and volume of payments in all four countries, indicating an approach to payments focused on breadth. At the country-level, in England, Novartis was the second biggest donor characterised by large payments; similar patterns characterised Biogen's payments in Scotland and Wales. England, Scotland and Northern Ireland all had at

For peer review only - http://bmjopen.bmj.com/site/about/guidelines.xhtml

least one top donor not featuring as a top donor in another country, indicating some companies' payments may be more targeted regionally than others.

Depth of engagement in the four countries

Companies making payments in England had the highest number of common recipients - an average of six to seven recipients (Table 2), implying a significant concentration of shared interest around a spectrum of organisations. Companies, on average, had at least one recipient in common with another company in Scotland and Wales, and were least connected in Northern Ireland (Table 2), indicating that in smaller countries, company interest in particular recipients is more concentrated. The average weighted degree density score shows the average number of recipients a company shares with *all* companies in the network, where similarly the highest score was observed in England (664.36 recipients) and lowest in Northern Ireland. The visualised networks are in Supplementary File 5.

Table 2. Pharmaceutical company connections in each country measured by common recipients

Network measure*	England	Scotland	Wales	Northern Ireland
Density – average value (average number of recipients in common between two companies)	6.71	1.24	1.13	0.42
Density – average weighted degree (average number of recipients in common for all companies in the network)	664.36	88.39	71.06	17.38
Company with highest degree centrality score (number of recipients a company has in common with all other companies in the network)	Pfizer (3,394)	Pfizer (319)	Pfizer (206)	Pfizer (63)

*Calculations were conducted on valued networks which means they consider the number of common recipients and not just the presence of a shared recipient. Networks include only companies making payments in each country.

The data also indicates variation in the depth of payments at the company level, as some companies focus collectively on particular recipients and some companies target a broader set of organisations with exclusive funding. Pfizer consistently targeted the same recipients as other companies most frequently in every country. Pfizer's degree centrality score of 3,394 in England shows that the company funded the same organisation as another company 3,394 times in the year (Table 2). Many of the most connected companies (see Supplementary File 6) were similar in England, Scotland and Wales. However, Northern Ireland's top ten most connected companies were more varied and featured smaller companies, suggesting that a cluster of companies had a unique interest Northern Ireland's health system. Further, differences between top donors and topmost connected companies in each country highlight potentially divergent strategies in targeted funding. For example, Merck Sharpe and Dohme was highly connected in every country but was not a top donor.

Coupled, the SNA and descriptive data provides evidence that some companies prioritise breadth of payments, targeting a broader spectrum of organisations, while other companies

For peer review only - http://bmjopen.bmj.com/site/about/guidelines.xhtml

prioritise depth, targeting recipients which seem important or 'popular' across the industry and potentially competing with other companies for visibility.

Structure of payments in each country

Structural differences in targeted recipient types between countries

The share of the total value of payments received by recipient types revealed diverse funding strategies in each country (Figure 1). In Wales and Scotland, industry targeted funding 'upstream' at healthcare commissioning, planning and regulatory organisations, primarily each country's local health boards that plan and deliver NHS services[80 81]. In Wales, they received just under half of all payments - £920,980.22 (46.38% of Wales' total payments, see Supplementary File 7 for values and Supplementary File 8 for top recipients). In Scotland, they received £878,333.57 (24.13%). Notably, the two Scottish health boards serving the fewest people received no payments. In England and Northern Ireland, funding was targeted 'downstream'. England's public sector secondary and tertiary care providers, namely consisting of NHS trusts which provide hospital and sometimes community healthcare services to residents[82], received the most funding (£13,349,779.1 – 25.56%). In Northern Ireland, public sector primary care providers, primarily general practitioner practices, were targeted with the most funding (£184,903.09 – 35.72%).

Figure 1. Percentage of payments to recipient types per country

There were statistically significant differences in the distribution of payments, indicating that payment values vary between the four countries even when the recipient type is the same (see Table 3). Post-hoc analyses maintained the significant differences, except for in patient organisations (see Supplementary File 9).

Patient organisations were a major target of payments, especially in England and Scotland (Table 3). Professional organisations, including societies and groups of healthcare professionals, were prioritised in England, Scotland and Northern Ireland, with significant but negligible differences in payment values. Consistent with the locations of the top UK universities, industry targeted education and research providers in England (median = £1000) and Scotland, (£1,152) where payments were also significantly higher than Wales (£336). Public sector primary care providers, primarily general practitioner practices, received a very small proportion of the total funding in England and Scotland, yet had the most individual recipients in all four countries, suggesting smaller per-recipient payment totals. This is further reflected in the median values per recipient, which were significantly lower in England (£435) and Scotland (£435) than Wales (£800) and Northern Ireland (£600).

Table 3. Differences in payment sizes between countries

Recipient type	Median (IQR) - £				***p* value**
	England	Scotland	Wales	Northern Ireland	
Alternative providers of health services	200 (160 – 394)	700 (360 – 1,100)	n/a	600 (550 - 600)	0.004*
Charities and other third-sector organisations†	223.52 (157 – 487.2)	1700 (377.5 - 6,250)	120 (120 - 180)	n/a	0.001*

For peer review only - http://bmjopen.bmj.com/site/about/guidelines.xhtml

Education and research providers	1000 (333.34 – 4,798.40)	1152 (400 – 2,880)	336 (175.2 - 1000)	1100 (873.75 - 3525)	0.001*
Formal bodies representing healthcare professionals	200 (160 – 259.8)	213.6 (211.4 - 214.8)	120 (96 - 142)	1066.67 (933.33 - 1200.00)	<.000*
Healthcare commissioning, planning and regulatory organisations	208.17 (160 – 307.2)	240 (131.18 - 500)	225 (114.24 - 486.39)	1500 (470.8 - 4,087)	0.008*
Patient organisations	4000 (500 – 11,104)	1000 (253.68 - 9,745)	747.93 (500 - 2,000)	650 (600 - 1,450)	0.011*
Private companies other than providers of health services	300 (196.8 – 598.92)	1,200 (350 - 5,528.88)	1475 (216 - 6,600)	4179.38 (1,152.19 - 7,687.5)	<.000*
Private sector healthcare providers	240 (166 – 588)	647.54 (206.65 - 1,500)	440 (360.94 - 1,732)	38.49 (28.9 - 485)	<.000*
Professional organisations	500 (240 – 3,200)	450 (285.67 - 980)	400 (280 - 800)	600 (320 - 1,784)	0.001*
Public administration and providers of public services	394.4 (224.45 – 546.67)	540 (200 - 600)	n/a	n/a	0.238
Public sector primary care providers	434.5 (193.6 – 869)	434.5 (202.23 - 651,75)	800 (434.5 - 1,152)	600 (434.5 - 1,600)	<.000*
Public sector secondary and tertiary care providers	233.17 (141.87 – 500)	300 (189.75 - 612)	253.66 (200 - 954)	288 (163.4 - 490.13)	0.055

*Statistically significant
†Excluding providers of health services, professional organisations and patient organisations

Extent of company connections in targeted recipient types in each country

Companies shared 5.8 common recipients on average among England's public sector secondary and tertiary care providers (Table 4), which also received the most funding. These patterns could be a function of the number of research-active NHS trusts located in England[83], meaning service providers might be very effective at getting donor funds, but also suggest a high degree of targeting by industry. Notably, although healthcare commissioning, planning and regulatory organisations, primarily clinical commissioning groups responsible for the planning and purchasing of local health care services[84], received very little funding in England, companies frequently target the same recipients, indicating that low funding does not infer an absence of interest.

In Scotland and Wales, companies targeted the same healthcare commissioning, planning and regulatory organisations most frequently, consistent with the financial prioritisation. In Northern Ireland, the density score for public sector primary care providers was higher than the other countries, suggesting some companies have overlapping interests in specific recipients in pockets of Northern Ireland's primary care system. In Wales, Scotland and Northern Ireland in particular, these patterns of common recipients pose a potentially greater risk to certain areas of the healthcare ecosystem becoming vulnerable to influence given the much smaller population the organisations serve.

For peer review only - http://bmjopen.bmj.com/site/about/guidelines.xhtml

Table 4. Density scores for valued recipient type networks in each country

**Recipient type**	**Density scores***			
	England	Scotland	Wales	Northern Ireland
Alternative providers of health services	0.339†	0.500	-	0.000
Charities and other third-sector organisations	0.510	0.333	0.476	-
Education and research providers	1.194	0.727	0.675	1.000
Formal bodies representing healthcare professionals	1.293	0.000	0.400	0.000
Healthcare commissioning, planning and regulatory organisations	2.523	1.578	1.634	0.133
Patient organisations	0.337	0.200	0.109	0.209
Private companies other than providers of health services	0.312	0.121	0.071	0.000
Private sector healthcare providers	0.416	0.167	0.167	0.067
Professional organisations	0.611	0.244	0.114	0.038
Public administration and providers of public services	0.022	0.300	0.000	-
Public sector primary care providers	0.893	0.038	0.124	1.600
Public sector secondary and tertiary care providers	5.819	1.309	1.000	0.826

* Density scores measure the average number of common recipients between two companies. The network matrix for each recipient type consisted only of companies making payments. Dashes indicate no payments were made. Scores of 0.000 indicate all recipients received payments from one company only.

†Example interpretation: a score of 0.339 indicates that each company making payments to alternative providers of health services funded, on average, 0.3 recipients in common with another company.

Prioritised payment types in each country

Another dimension of structure that differed between countries was the type of payments (Figure 2). Donations and grants, such as medical and educational goods, were consistently prioritised, however there was notable diversity between countries among the remaining payment types. Payments for joint working, defined as initiatives involving shared investment by the NHS and pharmaceutical companies[85], varied from 19.61% of all payments in Wales to 2.29% in Northern Ireland; fees for service and consultancy varied from 33.78% in Scotland to 4.86% in Northern Ireland; and contributions to costs of events, such as science or medical focused conferences and educational events, ranged from 31.87% in Northern Ireland to 18.58% in Wales.

For peer review only - http://bmjopen.bmj.com/site/about/guidelines.xhtml

Figure 2. Percentage of total value for each payment type

There was a statistically significant difference between the distribution of payments for costs of events (p = .000), which were lowest in Wales (£223) and highest in Northern Ireland (£478), and donations and grants (p = <.000), which were lowest in Northern Ireland (£435) and highest in England (£960). Differences in fees for service and consultancy (p = .995) and joint working (p = .261) were non-significant (see Supplementary File 10).

DISCUSSION

Principle findings

Our findings offer insights into the pharmaceutical industry's strategic approach to payments tailored to the policy and decision-making context between, and even within, each country. Our findings also indicate that the pharmaceutical industry's 'web of influence'[14] can be relatively structured and aligned with key within-country differences in health system design and processes, as well as cross-nationally. Our comparative analysis illustrates novel ethical and governance problems as well as commonalities across countries and confirms concerns that UK-level analysis[25 38] obscures important regional payment variations and recipient vulnerabilities[16]. The oversight of strategic specificity is important not least because key decisions about commissioning of health services are taken within each country[47 61].

Findings in context

Our findings align with previous comparative analyses of payments to teaching hospitals[31] and healthcare professionals in the United States, which show significant payment differences between regions[20 48 49 86]. Our findings also mirror those from a comparative study of industry payments to patient organisations in Denmark and Sweden, where larger payments were more frequent in the smallest country[16], suggesting a consistent industry strategy of targeting smaller locations with larger payments.

The concentration of payments among a few companies in each country was also consistent with previous studies of patient organisations[16 38 87] and healthcare organisations[25 31 61]. We identified Pfizer as a top donor, targeting many 'popular' recipients in all four UK countries, however it remains unclear if this relates to a particular product launch[40 88], a new push relative to emerging competition, or reflects a consistent trend. Further interpretation would be facilitated by longitudinal analysis. There were also differences in the companies providing the most funding, particularly in Northern Ireland where the top donors were similar to those making payments to healthcare organisations in the Republic of Ireland[28] rather than the other three UK countries, indicating that some companies may strategically target organisations on the island. One isolated case was Napp Pharmaceuticals, which featured as both a top donor and top-most connected company uniquely in Northern Ireland, suggesting that specific companies can dominate payment networks in relation to smaller countries under the radar. These instances may have direct implications for public health. For example, Napp Pharmaceuticals is an opioid manufacturer[89] and opioid manufacturers in the United States have been known to leverage targeted funding, including to teaching hospitals[31], to increase opioid prescribing[90].

For peer review only - http://bmjopen.bmj.com/site/about/guidelines.xhtml

Discrepancies in the types of payments prioritised also point towards sub-national vulnerabilities in each countries' healthcare ecosystem. In Wales, the prioritisation of joint working raises concerns around the extent of pharmaceutical industry involvement in healthcare design. Joint working arrangements are intended to bring benefits to patients, the NHS, and companies, however many of these projects mention increasing the use of company products[91], potentially serving as an alternative avenue for industry marketing. Similarly, in Northern Ireland, costs of events were higher than the other countries, pointing towards an alternative channel for industry involvement in continuing medical education in a country with fewer professional organisations or large universities. This pattern of frequent event payments was also observed in the Republic of Ireland[28], further indicating island-specific trends.

Lessons for transparency

The transparency concerns we identified are consistent with previous studies of pharmaceutical industry disclosure practices in the UK[4 42] and Europe[35 41]. Although the UK's self-regulatory payment disclosure system is the most robust in Europe[17 92], our analysis confirms earlier concerns about some payments being disclosed on the incorrect platform and thereby preventing their correct identification by policymakers, regulators, and members of the public[39 42]. Our findings indicate that some instances of under-reporting[39] may be explained by confusion about where to report.

These issues, coupled with the extensive additional research required to standardise and categorise recipient types and their locations in the UK, indicate that the self-regulatory system is incomplete and requires better integration. This could be achieved through a single standardised database comprising all pharmaceutical industry payments and combining the highest standards of reporting as they currently apply to, separately, healthcare and patient organisations. For example, EFPIA requires individual disclosures of payments to patient organisations to include descriptions of funded activities[42], a provision that should be extended to healthcare organisations. As a minimum, compulsory recipient identifiers should be introduced[35] to reduce the substantial forensic work involved in cleaning these data and encourage longitudinal comparisons. Echoing calls in the US for state-specific disclosure policies[51], Disclosure UK and disclosures of payments to patient organisations need to be adapted to better capture the distinction between payments in England, Scotland, Wales, and Northern Ireland.

Whilst baseline improvements in data accessibility and quality are imperative, a central database should also contain associated analytics, including descriptive and network statistics. Otherwise, we run the risk that pharmaceutical companies themselves gain more from the payment disclosure system than the public, as companies use disclosures to inform and fine-tune their marketing efforts[93 67].

Strengths and limitations

This is the first study to jointly analyse payments to healthcare and patient organisations, which was made possible by the current UK transparency provisions. It also is the first of its kind to explore payments across the four UK countries. To date, the spotlight has been on individual COIs, which may downplay the systemic problem of a broader institutional culture whereby industry funding is embraced and industry interests can be advanced[14 90].

For peer review only - http://bmjopen.bmj.com/site/about/guidelines.xhtml

However, our study has limitations. We focus only on 2015 data due to the substantial time required to prepare Disclosure UK data for effective analysis, particularly categorising recipients to make them distinguishable, and identifying recipient countries. We can assume the patterns are maintained over time as the overall payment values have remained stable each year[38 94], however longitudinal analysis would confirm this. Also, we could not determine whether sharing recipients was accidental or intentional, nor did we measure the impact of these payments.

Conclusion

Regional variability in payments has implications for sub-national policymaking[51] and it appears that there are specific vulnerabilities to potential institutional COIs arising at a sub-national level. These payment differences may also be occurring in other countries, particularly those with decentralised health system structures and/or high levels of independence across their decision-making authorities. Future research could examine factors contributing to regional payment differences to better inform future government or industry policies to mitigate against undue influence.

For peer review only

Ethical approval

The study did not require ethical approval (as it draws on publicly available data at the organisational level), however it is part of a bigger project which has ethical approval from the University of Bath's Social Sciences Research Ethics Committee (approval code: S19-073).

Funding statement

ER's work was supported by a +3 PhD Studentship award match-funded (50%) by the Economic and Social Research Council and the University of Bath. This work forms part of the PhD project. PO's work was supported by grants from The Swedish Research Council for Health, Working Life and Welfare (FORTE), no. 2016-00875, and The Swedish Research Council (VR), no. 2020-01822.

Competing interests statement

ER and EC have no conflicts of interests to declare. PO's PhD student was supported by a grant from Sigma Pharmaceuticals, a UK pharmacy wholesaler and distributor (not a pharmaceutical company). The PhD work funded by Sigma Pharmaceuticals is unrelated to the subject of this paper.

Author contributions

ER designed, managed, analysed and interpreted the data, as well as drafted the article. PO conceived and designed the study, provided supervision, and drafted the article. EC co-supervised the analysis and contributed to drafting the article.

Data availability statement

All data relevant to the study are included in the article or uploaded as supplementary information. The authors of this study agree to share data underpinning this study in the form of an Excel database available from the University of Bath Research Data Archive. The raw data poses no risk to anonymity of individuals as it draws on publicly available reports concerning financial transfers between organisations. The reference for this dataset is: Rickard, E., Ozieranski, P., 2023. *Dataset for "Comparing pharmaceutical company payments in the four UK countries: a cross-sectional and social network analysis".* Bath: University of Bath Research Data Archive. https://doi.org/10.15125/BATH-01239.

For peer review only

REFERENCES

1. Lexchin J. Pharmaceutical company spending on research and development and promotion in Canada, 2013-2016: a cohort analysis. *J Pharm Policy Pract* 2018;11:5-5. doi: 10.1186/s40545-018-0132-3
2. AHIP. New Study: In the Midst of COVID-19 Crisis, 7 out of 10 Big Pharma Companies Spent More on Sales and Marketing than R&D 2021 [Available from: https://www.ahip.org/news/articles/new-study-in-the-midst-of-covid-19-crisis-7-out-of-10-big-pharma-companies-spent-more-on-sales-and-marketing-than-r-d.
3. Swanson A. Big pharmaceutical companies are spending far more on marketing than research. *The Washington Post,* 2015.
4. Mulinari S, Ozieranski P. Disclosure of payments by pharmaceutical companies to healthcare professionals in the UK: analysis of the Association of the British Pharmaceutical Industry's Disclosure UK database, 2015 and 2016 cohorts. *BMJ Open* 2018;8(10):e023094. doi: 10.1136/bmjopen-2018-023094
5. Rodwin MA. Conflict of Interest in the Pharmaceutical Sector: A Guide for Public Management. *DePaul J Health Care L* 2019;21
6. Humphreys K, Shover CL, Andrews CM, et al. Responding to the opioid crisis in North America and beyond: recommendations of the Stanford-Lancet Commission. *The Lancet* 2022;399(10324):555-604. doi: 10.1016/S0140-6736(21)02252-2
7. Lo YL. Further insights on institutional conflicts of interest in research settings. *Proceedings of Singapore Healthcare* 2020;29(1):3-4. doi: 10.1177/2010105820909983
8. Schafer A. Institutional conflict of interest: attempting to crack the deferiprone mystery. *Journal of Medical Ethics* 2021;47(8):531. doi: 10.1136/medethics-2019-105498
9. Lo B, Field MJ. Conflict of interest in medical research, education, and practice. 2009
10. Lemmens T, Miller PB. The human subjects trade: ethical and legal issues surrounding recruitment incentives. *J Law Med Ethics* 2003;31(3):398-418. doi: 10.1111/j.1748-720x.2003.tb00103.x [published Online First: 2003/11/25]
11. Moynihan R, Bero L. Toward a Healthier Patient Voice: More Independence, Less Industry Funding. *JAMA Internal Medicine* 2017;177(3):350-51. doi: 10.1001/jamainternmed.2016.9179
12. Sismondo S. Ghost-managed medicine: Big pharma's invisible hands: Mattering Press 2018:8.
13. Rickard E, Ozieranski P. A hidden web of policy influence: The pharmaceutical industry's engagement with UK's All-Party Parliamentary Groups. *PLOS ONE* 2021;16(6):e0252551. doi: 10.1371/journal.pone.0252551
14. Marks JH. The perils of partnership: industry influence, institutional integrity, and public health: Oxford University Press 2019.
15. EFPIA. EFPIA CODE OF PRACTICE, 2019.
16. Mulinari S, Pashley D, Ozieranski P. Advancing international comparison of pharmaceutical industry funding of patient advocacy: Focus on Denmark. *Health Policy* 2022;126(12):1256-62. doi: https://doi.org/10.1016/j.healthpol.2022.11.003
17. Mulinari S, Martinon L, Jachiet P-A, et al. Pharmaceutical industry self-regulation and non-transparency: country and company level analysis of payments to healthcare professionals in seven European countries. *Health Policy* 2021;125(7):915-22. doi: https://doi.org/10.1016/j.healthpol.2021.04.015

For peer review only - http://bmjopen.bmj.com/site/about/guidelines.xhtml

18. Agrawal S, Brennan N, Budetti P. The Sunshine Act—effects on physicians. *N Engl J Med* 2013;2013(368):2054-57.

19. Ahmed R, Bae S, Hicks CW, et al. Here Comes the Sunshine: Industry's Payments to Cardiothoracic Surgeons. *Annals of Thoracic Surgery* 2017;103(2):567-72. doi: 10.1016/j.athoracsur.2016.06.053

20. Braithwaite J, Frane N, Partan MJ, et al. Review of Industry Payments to General Orthopaedic Surgeons Reported by the Open Payments Database: 2014 to 2019. *JAAOS Global Research & Reviews* 2021;5(5)

21. Fleischman W, Ross JS, Melnick ER, et al. Financial Ties Between Emergency Physicians and Industry: Insights From Open Payments Data. *Annals of Emergency Medicine* 2016;68(2):153-58.e4. doi: 10.1016/j.annemergmed.2016.01.014

22. Maruf M, Sidana A, Fleischman W, et al. Financial Relationships between Urologists and Industry: An Analysis of Open Payments Data. *Urology Practice* 2018;5(3):180-86. doi: 10.1016/j.urpr.2017.03.012

23. Slentz DH, Nelson CC, Lichter PR. Characteristics of Industry Payments to Ophthalmologists in the Open Payments Database. *JAMA Ophthalmology* 2019;137(9):1038-44. doi: 10.1001/jamaophthalmol.2019.2456

24. Moynihan R, Fabbri A, Parker L, et al. Mixed methods evaluation of workshops for citizen health advocates about financial conflicts of interests in healthcare. *BMJ Open* 2020;10(5) doi: 10.1136/bmjopen-2019-034195

25. Ozieranski P, Csanadi M, Rickard E, et al. Analysis of Pharmaceutical Industry Payments to UK Health Care Organizations in 2015. *JAMA Network Open* 2019;2(6):e196253-e53. doi: 10.1001/jamanetworkopen.2019.6253

26. Parker L, Karanges EA, Bero L. Changes in the type and amount of spending disclosed by Australian pharmaceutical companies: an observational study. *BMJ open* 2019;9(2):e024928.

27. Fabbri A, Grundy Q, Mintzes B, et al. A cross-sectional analysis of pharmaceutical industry-funded events for health professionals in Australia. *BMJ open* 2017;7(6):e016701.

28. Moriarty F, Larkin J, Fahey T. Payments reported by the pharmaceutical industry in Ireland from 2015 to 2019: an observational study. *Health Policy* 2021 doi: https://doi.org/10.1016/j.healthpol.2021.07.016

29. Ozaki A, Saito H, Senoo Y, et al. Overview and transparency of non-research payments to healthcare organizations and healthcare professionals from pharmaceutical companies in Japan: Analysis of payment data in 2016. *Health Policy* 2020;124(7):727-35. doi: https://doi.org/10.1016/j.healthpol.2020.03.011

30. Ozaki A, Saito H, Onoue Y, et al. Pharmaceutical payments to certified oncology specialists in Japan in 2016: a retrospective observational cross-sectional analysis. *BMJ Open* 2019;9(9):e028805. doi: 10.1136/bmjopen-2018-028805

31. Anderson TS, Gellad WF, Good CB. Characteristics of biomedical industry payments to teaching hospitals. *Health Affairs* 2020;39(9):1583-91. doi: 10.1377/hlthaff.2020.00385

32. Association of the British Pharmaceutical Industry. Working with patients and patient organisations. A sourcebook for industry, 2022.

33. McCoy MS, Carniol M, Chockley K, et al. Conflicts of interest for patient-advocacy organizations. *New England Journal of Medicine* 2017;376(9):880-85.

For peer review only

For peer review only - http://bmjopen.bmj.com/site/about/guidelines.xhtml

34. Rose SL, Highland J, Karafa MT, et al. Patient Advocacy Organizations, Industry Funding, and Conflicts of Interest. *JAMA Internal Medicine* 2017;177(3):344-50. doi: 10.1001/jamainternmed.2016.8443

35. Ozieranski P, Martinon, L., Jachiet, P., Mulinari, S. Accessibility and quality of drug company disclosures of payments to healthcare professionals and organisations in 37 countries: A European policy review. *BMJ Open* 2021

36. Fabbri A, Parker L, Colombo C, et al. Industry funding of patient and health consumer organisations: systematic review with meta-analysis. *BMJ* 2020;368:l6925. doi: 10.1136/bmj.l6925

37. EFPIA. EFPIA CODE OF PRACTICE ON RELATIONSHIPS BETWEEN THE PHARMACEUTICAL INDUSTRY AND PATIENT ORGANISATIONS, 2011.

38. Ozieranski P, Rickard E, Mulinari S. Exposing drug industry funding of UK patient organisations. *BMJ* 2019;365:l1806. doi: 10.1136/bmj.l1806

39. Ozieranski P, Csanadi M, Rickard E, et al. Underreporting of drug industry payments to patient organisations in the UK (2012-2016). 2020

40. Mulinari S, Vilhelmsson A, Rickard E, et al. Five years of pharmaceutical industry funding of patient organisations in Sweden: Cross-sectional study of companies, patient organisations and drugs. *PLOS ONE* 2020;15(6):e0235021. doi: 10.1371/journal.pone.0235021

41. Pashley D OP, Mulinari S. . Disclosure of pharmaceutical industry funding of patient organizations in Nordic countries: can industry self-regulation deliver on its transparency promise? *International Journal of Health Services* 2022

42. Rickard E, Ozieranski P, Mulinari S. Evaluating the transparency of pharmaceutical company disclosure of payments to patient organisations in the UK. *Health Policy* 2019;123(12):1244-50.

43. EFPIA. The EFPIA Disclosure Code: Your Questions Answered, 2014.

44. Association of the British Pharmaceutical Industry. Definitions - Disclosure 2022 [Available from: https://www.abpi.org.uk/reputation/disclosure-uk/about-disclosure-uk/definitions-disclosure/.

45. Keller AC, Packel L. Going for the cure: patient interest groups and health advocacy in the United States. *J Health Polit Policy Law* 2014;39(2):331-67. doi: 10.1215/03616878-2416238 [published Online First: 2013/12/07]

46. Hegde D, Sampat B. Can private money buy public science? Disease group lobbying and federal funding for biomedical research. *Management Science* 2015;61(10):2281-98.

47. Moberly T. CCGs fail to declare pharma funding. *BMJ* 2018;360:j5911. doi: 10.1136/bmj.j5911

48. Niforatos JD, Boas S, Hertz S, et al. Analysis of current financial relationships between emergency physicians and industry. *American Journal of Emergency Medicine* 2019;37(4):722-25. doi: 10.1016/j.ajem.2018.12.039

49. Cheng T, Boelitz K, Rybin D, et al. Nationwide patterns in industry payments to academic vascular surgeons. *Journal of Vascular Surgery* 2021;73(2):675-81. doi: 10.1016/j.jvs.2020.04.527

50. Garstka ME, Monlezun D, DuCoin C, et al. The Sunshine Act and surgeons: a nation-wide analysis of industry payments to physicians. *Journal of Surgical Research* 2019;233:41-49. doi: 10.1016/j.jss.2018.07.004

For peer review only - http://bmjopen.bmj.com/site/about/guidelines.xhtml

51. Cuomo RE, Cai M, Shah N, et al. Physicians payment in the United States between 2014 and 2018: An analysis of the CMS Open Payments database. *PLOS ONE* 2021;16(6):e0252656. doi: 10.1371/journal.pone.0252656
52. Mejia J, Mejia A, Pestilli F. Open data on industry payments to healthcare providers reveal potential hidden costs to the public. *Nature Communications* 2019;10(1) doi: 10.1038/s41467-019-12317-z
53. Ozieranski P, Pitter J, Rickard E, et al. A "patient-industry complex"? Investigating the financial dependency of UK patient organisations on drug company funding. *Sociology of Health & Illness* 2021
54. EFPIA. The Pharmaceutical Industry in Figures, 2016.
55. Hyndman N. Editorial: The charity sector— changing times, changing challenges. *Public Money & Management* 2017;37(3):149-53. doi: 10.1080/09540962.2017.1281608
56. Greer SL. Territorial politics and health policy: UK health policy in comparative perspective: Manchester University Press 2004.
57. Birrell D. Comparing devolved governance: Palgrave Macmillan 2012.
58. Cheung A, Paun A, Valsamidis L. Devolution at 20. Institute for Government, 2019.
59. ONS. Population estimates for the UK, England and Wales, Scotland and Northern Ireland: mid-2015 2016 [Available from: https://www.ons.gov.uk/peoplepopulationandcommunity/populationandmigration/populationestimates/bulletins/annualmidyearpopulationestimates/mid2015 accessed 13th September 2021.
60. Office for National Statistics. Life expectancy for local areas of the UK: between 2001 to 2003 and 2017 to 2019 2020 [Available from: https://www.ons.gov.uk/peoplepopulationandcommunity/healthandsocialcare/healthandlifeexpectancies/bulletins/lifeexpectancyforlocalareasoftheuk/between2001to2003and2017to2019 accessed 28th August 2021.
61. Saghy E, Mulinari S, Ozieranski P. Drug company payments to General Practices in England: cross-sectional and social network analysis. *PLOS ONE* 2021
62. Kirchhof P. A tale of two countries: how decentralized organization and long-term investment build resilient healthcare systems. *Eur Heart J Qual Care Clin Outcomes* 2020;6(3):201-03. doi: 10.1093/ehjqcco/qcaa036 [published Online First: 2020/05/07]
63. Martin-Moreno JM, Alonso P, Claveria A, et al. Spain: a decentralised health system in constant flux. *BMJ* 2009;338:b1170. doi: 10.1136/bmj.b1170
64. Rahul A. ea. Leveraging CMS Open Payments Data to Identify Channel Preferences and Gather Competitive Intelligence, Thereby Improving HCP Targeting. *Pharmaceutical Management Science Association* 2021
65. Hollands S. Receipt of Promotional Payments at the Individual and Physician Network Level Associated with Higher Branded Antipsychotic Prescribing Rates. *Adm Policy Ment Health* 2020;47(1):73-85. doi: 10.1007/s10488-019-00974-7
66. Agha L, Zeltzer D. DRUG DIFFUSION THROUGH PEER NETWORKS: THE INFLUENCE OF INDUSTRY PAYMENTS. *NBER WORKING PAPER SERIES* 2020;Working Paper 26338
67. Anand R CD, Yan J, et al. Leveraging CMS Open Payments Data to Identify Channel Preferences and Gather Competitive Intelligence, Thereby Improving HCP Targeting. *Journal of the Pharmaceutical Management Science Association* 2017 doi: https://www.pmsa.org/jpmsa-vol05-article06

For peer review only - http://bmjopen.bmj.com/site/about/guidelines.xhtml

68. Mulinari S, Ozieranski P. Capitalizing on transparency: Commercial surveillance and pharmaceutical marketing after the Physician Sunshine Act. *Big Data & Society* 2022;9(1):20539517211069631. doi: 10.1177/20539517211069631
69. Oldani MJ. Tales From The" Script": An Insider/Outside View Of Pharmaceutical Sales Practice. *Kroeber Anthropological Society Papers* 2002:147-76.
70. Association of the British Pharmaceutical Industry. CODE OF PRACTICE for the PHARMACEUTICAL INDUSTRY 2015, 2015.
71. Dunn OJ. Multiple Comparisons Using Rank Sums. *Technometrics* 1964;6(3):241-52. doi: 10.1080/00401706.1964.10490181
72. Baadh AS, Baadh PK, Islam S, et al. IR and the Sunshine Act: Two-Year Analysis of the Open Payments Database and Comparison with Related Specialties. *Journal of Vascular and Interventional Radiology* 2017;28(2):200-05. doi: 10.1016/j.jvir.2016.10.008
73. Morse E, Fujiwara RJT, Mehra S. Increasing Industry Involvement in Otolaryngology: Insights from 3 Years of the Open Payments Database. *Otolaryngology - Head and Neck Surgery (United States)* 2018;159(3):501-07. doi: 10.1177/0194599818778502
74. Baadh AS, Patel M, Patel K, et al. Medical industry at tumor board: Three-years analysis of the open payments database and comparison of oncologic specialties. *Journal of Surgical Oncology* 2018;117(4):551-57. doi: 10.1002/jso.24899
75. Borgatti SP, Everett MG, Johnson JC. Analyzing social networks: Sage 2018.
76. Snijders TA, Faye M, Brailly J. Network dynamics with a nested node set: Sociability in seven villages in Senegal. *Statistica Neerlandica* 2020;74(3):300-23.
77. Borgatti SP, Everett, M.G. and Freeman, L.C. . Ucinet for Windows: Software for Social Network Analysis. Harvard, MA: Analytic Technologies 2002.
78. Borgatti S. Centrality and Centralization 2000 [Available from: http://www.analytictech.com/mb119/chapter5.htm accessed 20th July 2021.
[dataset] 79. Rickard E, Ozieranski, P.,. Dataset for "Comparing pharmaceutical company payments in the four UK countries: a cross-sectional and social network analysis": Bath: University of Bath Research Data Archive, 2023.
80. Welsh Government. NHS Wales health boards and trusts 2022 [Available from: https://www.gov.wales/nhs-wales-health-boards-and-trusts.
81. NHS Scotland. NHSScotland Health Boards 2022 [Available from: https://www.careers.nhs.scot/careers/find-your-career/international-recruitment/nhsscotland-health-boards/.
82. Foundation Trust Network. BUILDING A HEALTHY NHS AROUND PEOPLE'S NEEDS. AN INTRODUCTION TO NHS FOUNDATION TRUSTS AND TRUSTS, 2015.
83. Noclor. EVERY NHS TRUST IN ENGLAND IS NOW RESEARCH-ACTIVE 2022 [Available from: https://www.noclor.nhs.uk/every-nhs-trust-england-now-research-active.
84. NHS Confederation. What are clinical commissioning groups? 2021 [Available from: https://www.nhsconfed.org/articles/what-are-clinical-commissioning-groups.
85. Association of the British Pharmaceutical Industry. Joint Working: A toolkit for industry and the NHS, 2019.
86. Brauer PR, Morse E, Mehra S. Industry Payments for Otolaryngology Research: A Four-Year Analysis of the Open Payments Database. *Laryngoscope* 2020;130(2):314-20. doi: 10.1002/lary.27896

For peer review only - http://bmjopen.bmj.com/site/about/guidelines.xhtml

87. Fabbri A, Swandari S, Lau E, et al. Pharmaceutical industry funding of health consumer groups in Australia: a cross-sectional analysis. *International Journal of Health Services* 2019;49(2):273-93.
88. Baggott R, Allsop J, Jones K. Speaking for patients and carers: health consumer groups and the policy process: Macmillan International Higher Education 2014.
89. Keefe PR. Empire of pain: The secret history of the Sackler dynasty: Anchor 2021.
90. Marks JH. Lessons from Corporate Influence in the Opioid Epidemic: Toward a Norm of Separation. *Journal of Bioethical Inquiry* 2020;17(2):173-89. doi: 10.1007/s11673-020-09982-x
91. Moberly T. NHS joint working with industry is out of public sight. *BMJ* 2019;364:l1353. doi: 10.1136/bmj.l1353
92. Ozaki POHSERSMA. International comparison of pharmaceutical industry payment disclosures in the UK and Japan: implications for self-regulation, public regulation, and transparency. *Globalization and Health* 2023 doi: 10.1186/s12992-022-00902-9
93. Kanter GP, Loewenstein G. Evaluating open payments. *Jama* 2019;322(5):401-02.
94. Association of the British Pharmaceutical Industry. Disclosure UK 2022 [Available from: https://www.abpi.org.uk/reputation/disclosure-uk/.

For peer review only - http://bmjopen.bmj.com/site/about/guidelines.xhtml

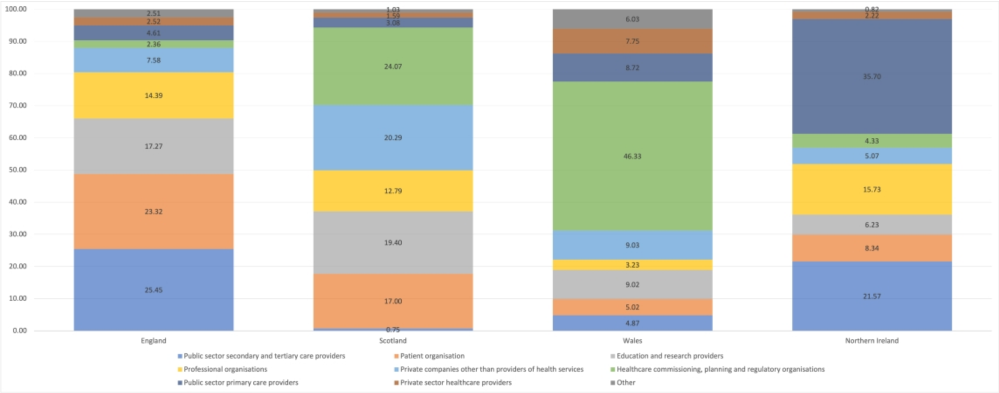

Percentage of payments to recipient types per country

541x213mm (300 x 300 DPI)

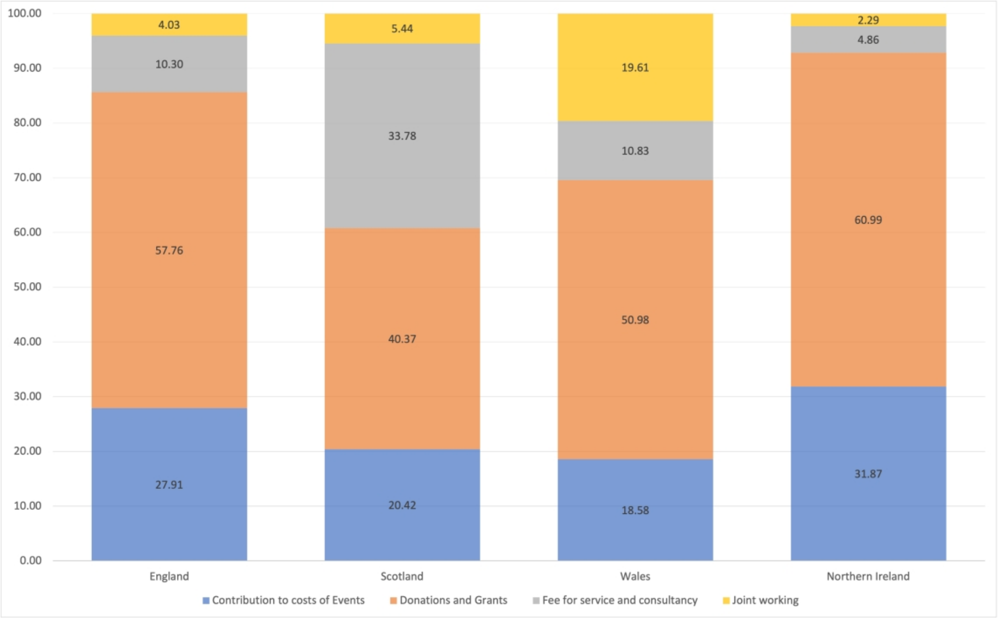

Percentage of total value for each payment type

346x214mm (300 x 300 DPI)

Supplementary file contents:

Supplementary File	Page
Supplementary File 1. Payment types included in Disclosure UK and patient organisation codes applied to Disclosure UK codes	2
Supplementary File 2. Data integration flowchart	3
Supplementary File 3. Recipient category descriptions and examples	4-5
Supplementary File 4. Top 10% of donors in each country	6
Supplementary File 5. Visualised networks for England (a), Scotland (b), Wales (c), Northern Ireland (d)	7-10
Supplementary File 6. Top ten companies by degree centrality scores in each country	11
Supplementary File 7. Descriptive statistics for each recipient category	12-14
Supplementary File 8. Top 10 recipients in each country	15-16
Supplementary File 9. Post-hoc Bonferroni pairwise comparisons between countries of payments per category	17-19
Supplementary File 10. Post-hoc Bonferroni pairwise comparisons between countries of payment types	20

For peer review only

Supplementary File 1. Payment types included in Disclosure UK and patient organisation codes applied to Disclosure UK codes

Payment type	Description of payment type	Patient organisation payments subsumed within the payment type
Contribution to costs of Events	Contribution to costs related to Events, through HCOs or Third Parties, including support to HCPs to attend Events, such as: • Registration fees; • Sponsorship agreements with HCOs or with Third Parties appointed by an HCO to manage an Event; and • Travel and accommodation (EFPIA Code of Practice 2019, p. 30)	contributions to costs of events organised by recipients or third parties; travel, accommodation and registration fees
Donations and Grants to HCOs	Donations and Grants to HCOs that support healthcare, including donations and grants (either cash or benefits in kind) to institutions, organisations or associations that are comprised of HCPs and/or that provide healthcare (EFPIA Code of Practice 2019, p. 30)	donations; grants; corporate member, supporter, sponsor or partner; purchases and subscriptions from patient organisations; more than one distinct payment form; form of funding unclear; sponsorships
Fee for service and consultancy	Payments resulting from or related to contracts between Member Companies and HCOs under which such HCOs provide any type of services to a Member Company or any other type of funding not covered in the previous categories. Fees, on the one hand, and on the other hand payments relating to expenses agreed in the written agreement covering the activity will be disclosed as two separate amounts. (EFPIA Code of Practice 2019, p. 30)	fees for service and consultancy (including travel and accommodation); support, help and contributions
Joint working	The Department of Health defines joint working between the NHS and the pharmaceutical industry as situations where, for the benefit of patients, one or more pharmaceutical companies and the NHS pool skills, experience and/or resources for the joint development and implementation of patient centred projects and share a commitment to successful delivery. (ABPI Code of Practice 2015, Clause 20, p. 30)	n/a

For peer review only - http://bmjopen.bmj.com/site/about/guidelines.xhtml

Supplementary File 2. Data integration flowchart

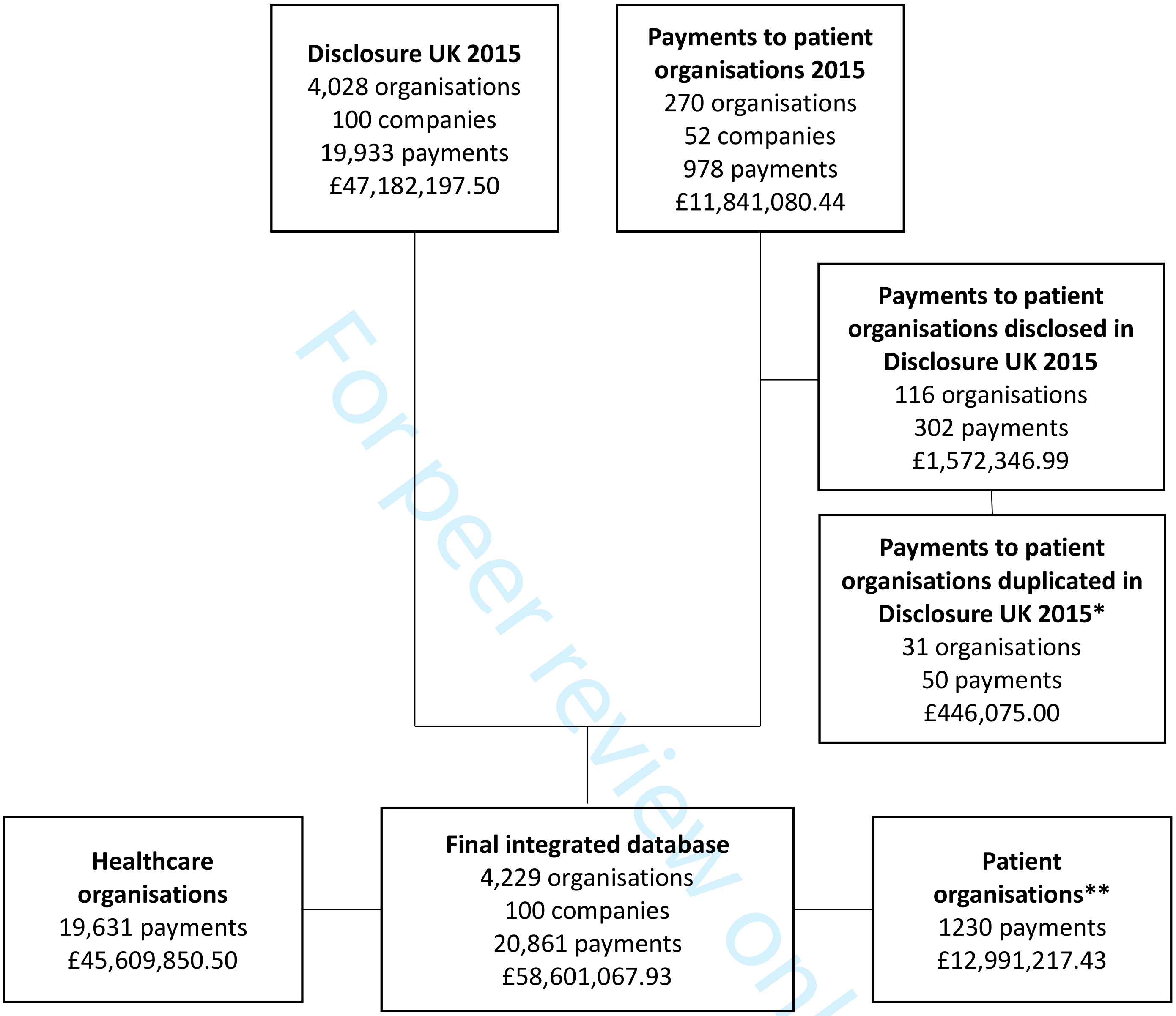

For peer review only

*This is the number and value of payments excluded to ensure no payment was counted twice
**During the cleaning process, a number of considerations took place to determine the final number and value of payments to patient organisations. Some duplicate payments were identified that were reported as multiple payments in one dataset and one payment in the other dataset (influencing the final number of payments). Approaches to VAT when reporting values also differed between the two datasets (influencing the final value of payments).

Supplementary File 3. Recipient category descriptions and examples

Recipient category	Category description	Examples (country-specific examples where applicable)
Alternative providers of health services	Charities, not-for-profit companies, social enterprises and community interest companies providing health services	- social enterprise delivering primary or secondary care health services<br>- nursing or care home run by a community interest company (CIC)<br>- hospital, hospice, or nursing home with a charitable status
Education and research providers	Universities, charities, and noncommercial institutes undertaking research	- university<br>- research institute at a NHS organisation<br>- charity focusing on undertaking medical research
Formal bodies representing healthcare professionals or patients	Local medical, optical, optometric, or pharmaceutical committees and statutory bodies representing healthcare professionals or patients	- local medical committees (LMC)<br>- local optical or optometric committee (LOC)<br>*England*<br>- local pharmaceutical committee (LPC)
Charities and other third-sector organisations (excluding providers of health services, professional organisations, and patient organisations)	Organisations (not patient organisations) focusing on education, research, advocacy, and multipurpose organisations	- charitable trusts providing medical education events to healthcare professionals<br>- think tanks<br>- third-sector organisation (non-charity) or charity focused on funding medical research<br>- research institute registered as a charitable organisation
Healthcare commissioning, planning and regulatory organisations	Local, regional, and commissioning, planning, or regulatory organisations	- primary care trust (PCT)<br>- NHS Shared Business Services<br>- National Institute for Health and Care Excellence (NICE)<br>- Public Health England<br>*England*<br>- clinical commissioning group<br>- Locality group<br>- area prescribing committee<br>- local commissioning group<br>- NHS England<br>*Scotland*<br>- regional NHS board<br>- area pharmaceutical committee<br>*Wales*<br>- health board<br>- public health Wales

For peer review only - http://bmjopen.bmj.com/site/about/guidelines.xhtml

		Northern Ireland<br>- health and social care board<br>- local commissioning group
Patient organisations	Organisations focusing on supporting education, research, advocacy, and multipurpose organisations	- multipurpose patient organisations<br>- organisations focused on providing patient support<br>- hospital charities
Private companies other than providers of health services	Providers of medical communications or training services, commercial or medical research services, and accountancy or consultancy services	- manufacturer or supplier of medical devices or technologies<br>- pharmacy wholesaler or distributor<br>- event management services<br>- journal or publishing company<br>- clinical or contract research organisation<br>- private laboratory
Private sector healthcare providers	Private clinics and hospitals, healthcare groups, and providers of dental, pharmacy, and optical services	- dental practice<br>- pharmacy or chemist<br>- opticians<br>- private clinic, surgery, practice, or hospital<br>- private company providing community health or social care services
Professional organisations	Organisations of medical professionals, other healthcare professionals, or non-healthcare professionals,	- organisation of medical professionals<br>- professional bodies responsible for setting standards of care and education for medical specialities<br>- royal college - medical professionals<br>- alliance or coalition of professional associations or groups<br>- professional organisation of pharmacists or pharmacy technicians
Public administration and providers of public services	Central UK government bodies, devolved administrations in Scotland, Wales, and Northern Ireland, and local authorities	- district, city, country, or borough council<br>- prison<br>- devolved administrations<br>- central government bodies
Public sector primary care providers	General practitioner surgeries, medical practice centres, groups of surgeries or medical practices, and healthcare or medical groups	- GP practice, surgery, medical practice or family practice<br>- health centre, medical centre or primary care centre<br>- group of surgeries or medical practices
Public sector secondary and tertiary care providers	NHS trusts, NHS hospitals, and networks and collaboratives of NHS organisations	- NHS hospital<br>- NHS Foundation Trust<br>- NHS rust<br>- strategic clinical network (SCN)<br>*Scotland*<br>- managed clinical network (MCN)

For peer review only

Supplementary File 4. Top 10% of donors in each country

Country	Company (revenue ranking)	Payment value - £ (%)	Payments – n (%)
England	Pfizer (2)	5,292,130.74 (10.09)	1636 (8.99)
	Novartis (7)	3,564,500.43 (6.80)	460 (2.53)
	Bayer (8)	3,476,304.44 (6.63)	2110 (11.60)
	GlaxoSmithKline (4)	3,291,496.35 (6.28)	1076 (5.92)
	AstraZeneca (1)	2,779,000.54 (5.30)	1279 (7.03)
	Janssen-Cilag (10)	2,387,242.64 (4.55)	722 (3.97)
	UCB Pharma (30)	2,204,967.90 (4.20)	74 (0.41)
	Astellas Pharma (21)	2,044,050.60 (3.90)	311 (1.71)
	Roche (5)	1,931,651.77 (3.68)	173 (0.95)
	Biogen Idec (23)	1,886,879.26 (3.60)	83 (0.46)
	Top 10% total	28,858,224.67 (55.03)	7924 (43.56)
Scotland	Biogen Idec (24)	733,104.05 (20.09)	7 (0.51)
	Takeda UK (38)	274,952.71 (7.53)	25 (1.82)
	Pfizer (2)	250,859.45 (6.87)	143 (10.44)
	Bayer (8)	215,930.76 (5.92)	164 (11.97)
	Novartis (7)	199,703.97 (5.47)	48 (3.50)
	Bristol-Myers Squibb (23)	183,959.00 (5.04)	51 (3.72)
	AstraZeneca (1)	178,848.49 (4.90)	75 (5.47)
	Top 10% total	2,037,358.42 (55.82)	513 (37.45)
Wales	Pfizer (2)	284,719.57 (14.32)	102 (10.30)
	Roche (5)	230,090.90 (11.58)	10 (1.01)
	Novartis (7)	177,069.59 (8.91)	36 (3.64)
	AstraZeneca (1)	148,288.67 (7.46)	79 (7.98)
	Janssen-Cilag (10)	122,237.44 (6.15)	22 (2.22)
	Biogen (24)	112,428.62 (5.66)	7 (0.71)
	Top 10% total	1,074,834.80 (54.07)	256 (25.86)
Northern Ireland	Sanofi Aventis (13)	92,252.80 (17.81)	24 (7.72)
	Pfizer (2)	86,639.31 (16.73)	45 (14.47)
	Napp Pharmaceuticals (29)	83,252.29 (16.07)	45 (14.47)
	Bayer (8)	37,959.50 (7.33)	75 (24.12)
	Top 10% total	300,103.90 (57.94)	189 (60.77)

*Value as proportion of all payments in each country

For peer review only - http://bmjopen.bmj.com/site/about/guidelines.xhtml

Supplementary File 5. Visualised networks for England (a), Scotland (b), Wales (c), Northern Ireland (d)

Supplementary File 5a. England's network

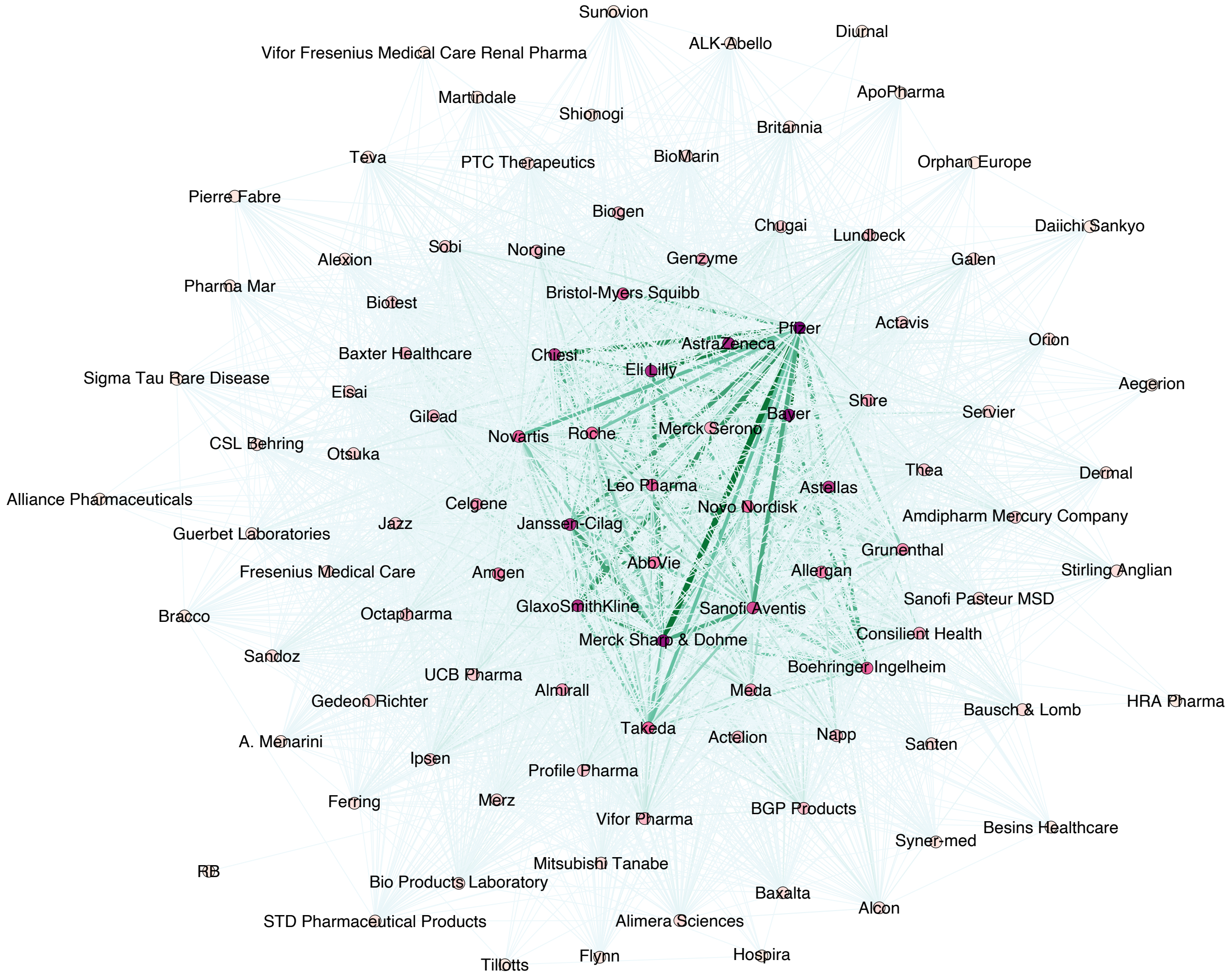

only

Supplementary File 5b. Scotland's network

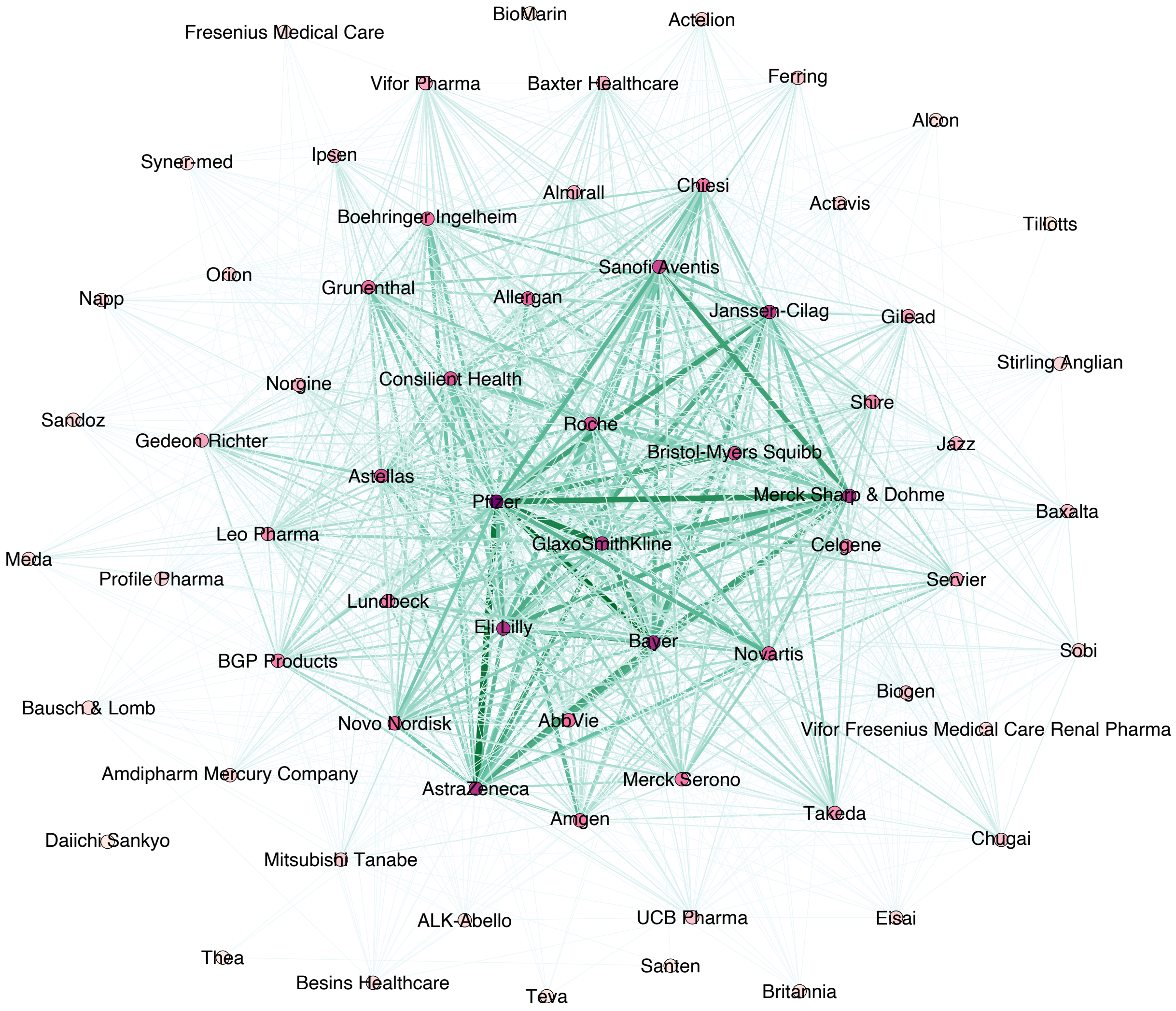

For peer review only - http://bmjopen.bmj.com/site/about/guidelines.xhtml

Supplementary File 5c. Wales' network

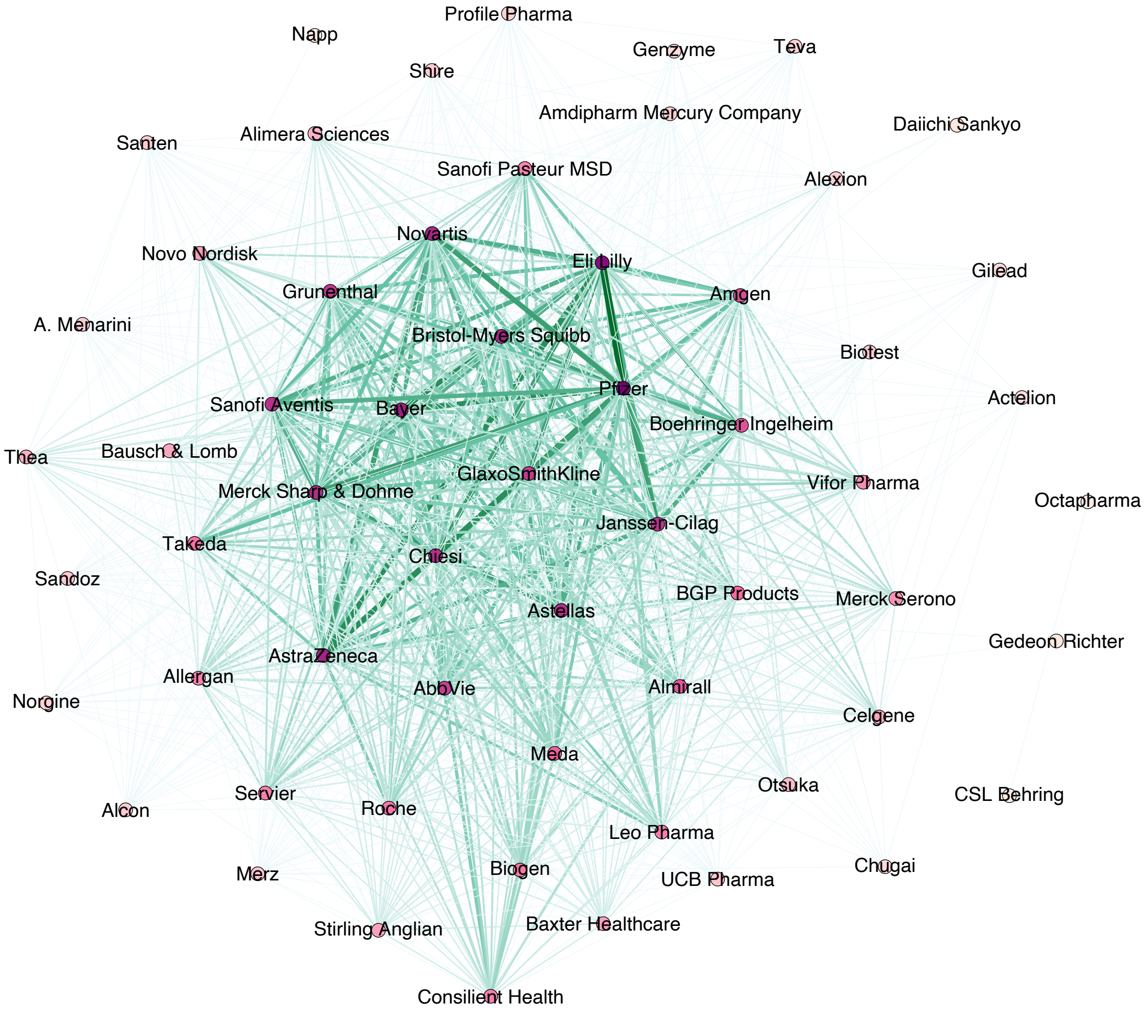

Supplementary File 5d. Northern Ireland's network

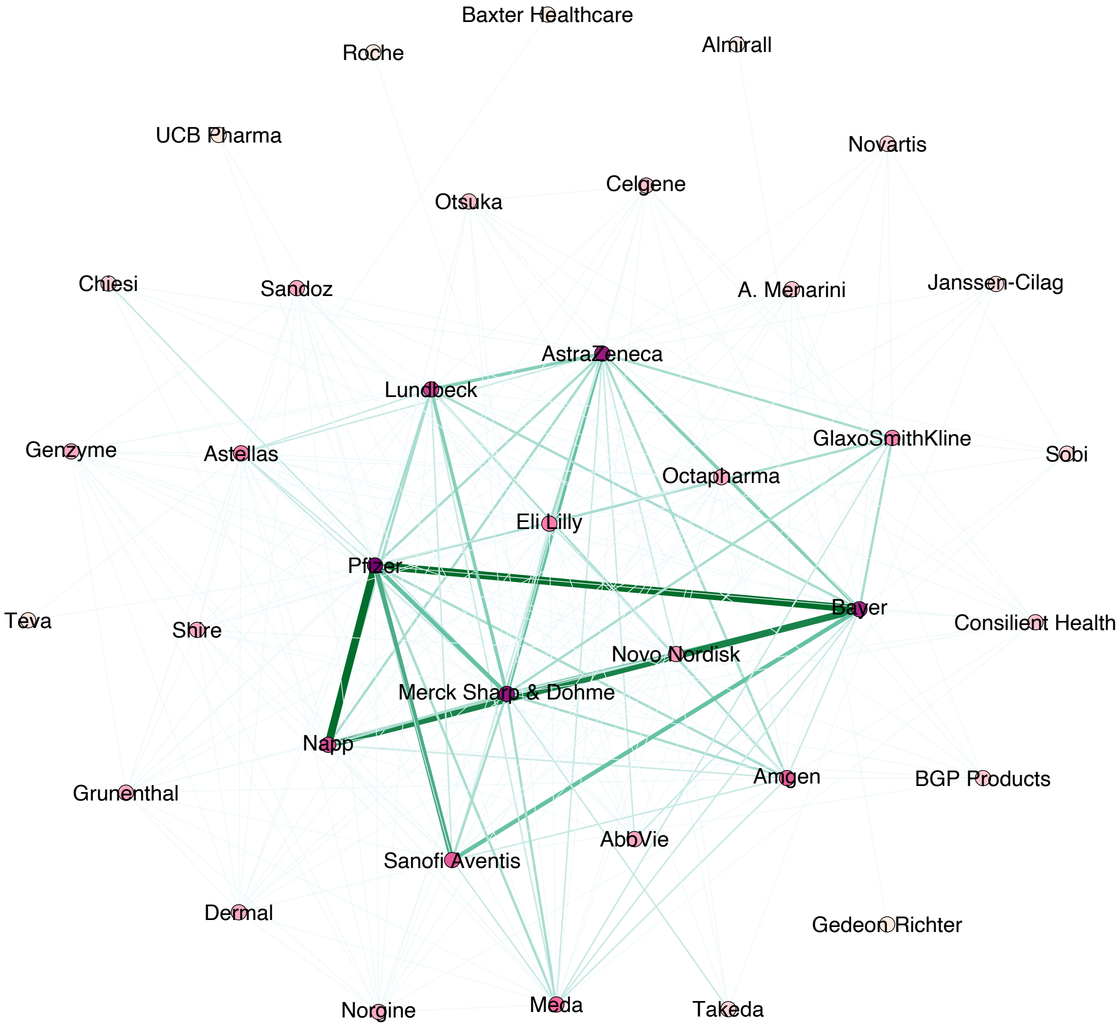

Note. All networks were visualised in Gephi v 0.9.2. Node label size and darkness corresponds to the weighted degree centrality of each company; the size and darkness of the edges (connecting lines) correspond to the number of shared recipients between companies

For peer review only - http://bmjopen.bmj.com/site/about/guidelines.xhtml

Supplementary File 6. Top ten companies by degree centrality scores in each country

England		Scotland		Wales		Northern Ireland	
Company	Degree	Company	Degree	Company	Degree	Company	Degree
Pfizer (2)	3394	Pfizer (2)	319	Pfizer (2)	206	Pfizer (2)	63
Merck Sharp & Dohme (6)	3064	Bayer (8)	260	Eli Lilly (3)	196	Merck Sharp & Dohme (6)	57
Bayer (8)	3060	Merck Sharp & Dohme (6)	251	Bayer (8)	176	AstraZeneca (1)	55
AstraZeneca (1)	2755	AstraZeneca (1)	245	AstraZeneca (1)	171	Bayer (8)	52
Eli Lilly (3)	2741	Eli Lilly (3)	245	Bristol-Myers Squibb (23)	169	Lundbeck (64)	43
Janssen-Cilag (10)	2539	GlaxoSmithKline (4)	241	Novartis (7)	168	Napp Pharmaceuticals (29)	39
GlaxoSmithKline (4)	2531	Janssen-Cilag (10)	227	Merck Sharp & Dohme (6)	161	Amgen (19)	36
Astellas Pharma (21)	2410	Sanofi Aventis (13)	214	Astellas Pharma (21)	160	Sanofi Aventis (13)	36
Chiesi (27)	2365	Astellas Pharma (21)	196	Chiesi (27)	158	Meda Pharma (46)	32
Sanofi Aventis (13)	2156	Bristol-Myers Squibb (23)	193	Sanofi Aventis (13)	156	Eli Lilly (3)	27

Note. Numbers in brackets represent company size (revenue ranking)

For peer review only

Supplementary File 7. Descriptive statistics for each recipient type

**England – types of recipient**	**Value (%)**	**Payments – n (%)**	**Median - £ (IQR)**	**Recipients – n (%)**	**Pharmaceutical companies**
Public sector secondary and tertiary care providers	**13,349,779.1 (25.56)**	**6660 (36.87)**	233.17 (141.87 – 500)	260 (7.41)	**89**
Patient organisation	12,227,843.2 (23.41)	1141 (6.32)	**4000 (500 – 11,104)**	288 (8.21)	65
Education and research providers	9,055,882.96 (17.34)	875 (4.84)	1000 (333.34 – 4,798.40)	56 (1.60)	68
Professional organisations	7,545,121.68 (14.44)	1776 (9.83)	500 (240 – 3,200)	354 (10.09)	84
Private companies other than providers of health services	3,975,461.63 (7.61)	1443 (7.99)	300 (196.8 – 598.92)	239 (6.81)	56
Public sector primary care providers	2,416,957.98 (4.63)	2513 (13.91)	434.5 (193.6 – 869)	**1809 (51.55)**	32
Private sector healthcare providers	1,322,785.04 (2.53)	463 (2.56)	240 (166 – 588)	108 (3.08)	44
Healthcare commissioning, planning and regulatory organisations	1,235,239.68 (2.36)	2166 (11.99)	208.17 (160 – 307.2)	206 (5.87)	47
Charities and other third-sector organisations	876,822.76 (1.68)	366 (2.03)	223.52 (157 – 487.2)	39 (1.11)	40
Formal bodies representing healthcare professionals or patients	121,351.93 (0.23)	458 (2.54)	200 (160 – 259.8)	68 (1.94)	25
Alternative providers of health services	93,534.91 (0.18)	180 (1.00)	200 (160 – 394)	62 (1.77)	28
Public administration and providers of public services	15,335.25 (0.03)	24 (0.13)	394.4 (224.45 – 546.67)	20 (0.57)	10
All payments	*52,445,615.48*	*18065*	*280 (160 – 827.75)*	*3509*	*100*
**Scotland - types of recipient**	**Value (%)**	**Payments - n (%)**	**Median - £ (IQR)**	**Recipients - n (%)**	**Pharmaceutical companies (%)**
Healthcare commissioning, planning and regulatory organisations	**878,333.57 (24.13)**	**582 (43.30)**	240 (131.18 - 500)	22 (8.30)	**53 (73.61)**
Private companies other than providers of health services	740,694.09 (20.35)	25 (1.86)	1,200 (350 - 5,528.88)	11 (4.15)	13 (18.06)
Education and research providers	708,149.16 (19.46)	141 (10.49)	1152 (400 – 2,880)	8 (3.02)	41 (56.94)
Patient organisation	620,384.33 (17.05)	52 (3.87)	1000 (253.68 - 9,745)	14 (5.28)	19 (26.39)
Professional organisations	466,833.11 (12.83)	291 (21.65)	450 (285.67 - 980)	64 (24.15)	52 (72.22)

For peer review only - http://bmjopen.bmj.com/site/about/guidelines.xhtml

Public sector primary care providers	112,308.91 (3.09)	128 (9.52)	434.5 (202.23 - 651,75)	**113 (42.64)**	13 (18.06)
Private sector healthcare providers	58,091.76 (1.60)	69 (5.13)	647.54 (206.65-1,500)	11 (4.15)	9 (12.50)
Public sector secondary and tertiary care providers	27,392.82 (0.75)	39 (2.90)	300 (189.75 - 612)	12 (4.53)	11 (15.28)
Charities and other third-sector organisations	19,710 (0.54)	4 (0.30)	**1700 (377.5 - 6,250)**	2 (0.75)	4 (5.56)
Alternative providers of health services	4,580 (0.13)	6 (0.45)	700 (360 – 1,100)	4 (1.51)	4 (5.56)
Public administration and providers of public services	2,700 (0.07)	5 (0.37)	540 (200 - 600)	3 (1.13)	5 (6.94)
Formal bodies representing healthcare professionals or patients	427.2 (0.01)	2 (0.15)	213.6 (211.4 - 214.8)	1 (0.38)	1 (1.39)
All payments	*3,649,749.43*	*1,344*	*400 (180 - 864)*	*265*	*72*
**Wales - types of recipient**	**Value (%)**	**Payments - n (%)**	**Median - £ (IQR)**	**Recipients - n (%)**	**Pharmaceutical companies (%)**
Healthcare commissioning, planning and regulatory organisations	**920,980.22 (46.38)**	**557 (56.61)**	225 (114.24 - 486.39)	10 (4.72)	**50 (78.13)**
Private companies other than providers of health services	179,495.4 (9.04)	56 (5.69)	**1475 (216 - 6,600)**	10 (4.72)	8 (12.50)
Education and research providers	179,256.38 (9.03)	37 (3.76)	336 (175.2 - 1000)	5 (2.36)	16 (25.00)
Public sector primary care providers	173,268.30 (8.73)	141 (14.33)	800 (434.5 - 1,152)	**118 (55.66)**	15 (23.44)
Private sector healthcare providers	153,983.36 (7.76)	18 (1.83)	440 (360.94 - 1,732)	6 (2.83)	9 (14.06)
Public administration and providers of public services	108,000 (5.44)	1 (0.10)	-	1 (0.47)	1 (1.56)
Patient organisation	99,784.32 (5.03)	22 (2.24)	747.93 (500 - 2,000)	10 (4.72)	11 (17.19)
Public sector secondary and tertiary care providers	96,862.66 (4.88)	20 (2.03)	253.66 (200 - 954)	3 (1.42)	13 (20.31)
Professional organisations	64,181.82 (3.23)	88 (8.94)	400 (280 - 800)	38 (17.92)	31 (48.44)
Charities and other third-sector organisations	5,036.8 (0.25)	17 (1.73)	120 (120 - 180)	4 (1.89)	7 (10.94)
Formal bodies representing healthcare professionals or patients	4,679.37 (0.24)	27 (2.74)	120 (96 - 142)	7 (3.30)	11 (17.19)
All payments	*1,987,702.62*	*984*	*300 (144 - 800)*	*212*	*64*

For peer review only - http://bmjopen.bmj.com/site/about/guidelines.xhtml

**Northern Ireland - types of recipient**	**Value (%)**	**Payments - n (%)**	**Median - £ (IQR)**	**Recipients - n (%)**	**Pharmaceutical companies (%)**
Public sector primary care providers	**184,903.09 (35.72)**	**127 (40.97)**	600 (434.5 - 1,600)	**94** (60.65)	6 (14.29)
Public sector secondary and tertiary care providers	111,743.45 (21.59)	83 (26.77)	288 (163.4 - 490.13)	5 (3.23)	**27 (64.29)**
Professional organisations	81,489.7 (15.74)	34 (10.97)	600 (320 - 1,784)	21 (13.55)	21 (50.00)
Patient organisation	43,205.6 (8.35)	15 (4.84)	650 (600 - 1,450)	7 (4.52)	14 (33.33)
Education and research providers	32,258 (6.23)	10 (3.23)	1100 (873.75 - 3525)	1 (0.65)	7 (16.67)
Private companies other than providers of health services	26,242.77 (5.07)	6 (1.94)	**4179.38 (1,152.19 - 7,687.5)**	4 (2.58)	3 (7.14)
Healthcare commissioning, planning and regulatory organisations	22,447.6 (4.34)	7 (2.26)	1500 (470.8 - 4,087)	4 (2.58)	6 (14.29)
Private sector healthcare providers	11,476.85 (2.22)	23 (7.42)	38.49 (28.9 - 485)	16 (10.32)	6 (14.29)
Formal bodies representing healthcare professionals or patients	2,133.34 (0.41)	2 (0.65)	1066.67 (933.33 - 1200.00)	1 (0.65)	1 (2.38)
Alternative providers of health services	1,700 (0.33)	3 (0.97)	600 (550 - 600)	2 (1.29)	2 (4.76)
All payments	*517,600.40*	*310*	*475.2 (217.25 - 1,357.47)*	*155*	*42*

For peer review only

Supplementary File 8. Top 10 recipients in each country

Country	Recipient	Type of recipient	Value - £	Payments - n	Companies - n
England	King's College London	Education and research providers	2,572,086.51	45	18
	Bladder and Bowel Foundation	Patient organisation	1,459,371.52	11	1
	London School Hyg and Tropical Med	Education and research providers	935,025.98	16	6
	PeerVoice	Private companies other than providers of health services	930,028.30	11	3
	University College London	Education and research providers	907,256.40	96	36
	Diabetes UK - England	Patient organisation	888,845.00	41	7
	Healthcare At Home	Private sector healthcare providers	872,740.81	18	2
	Cancer Research UK	Patient organisation	804,543.76	19	9
	Central Manchester Univ Hosps FT	Public sector secondary and tertiary care providers	739,595.97	108	37
	British Society for Rheumatology	Professional organisations	543,012.33	31	14
Scotland	Quintiles - Scotland	Private companies other than providers of health services	682,601.65	5	1
	Myeloma UK	Patient organisation	521,574.36	12	7
	NHS Greater Glasgow and Clyde	Healthcare commissioning, planning and regulatory organisations	483,354.99	153	34
	University of Glasgow	Education and research providers	442,707.63	70	26
	University of Dundee	Education and research providers	160,632.40	20	11
	NHS Lothian	Healthcare commissioning, planning and regulatory organisations	144,175.05	73	25
	University of Edinburgh	Education and research providers	73,014.39	37	21
	NHS Tayside	Healthcare commissioning, planning and regulatory organisations	67,924.08	74	24
	NHS Ayrshire and Arran	Healthcare commissioning, planning and regulatory organisations	63,276.93	48	25
	Digestive Disorders Federation	Professional organisations	60,796.00	2	2
Wales	Cardiff and Vale University HB	Healthcare commissioning, planning and regulatory organisations	344,131.95	89	28

For peer review only

	Abertawe Bro Morgannwg Univ HB	Healthcare commissioning, planning and regulatory organisations	242,418.82	124	32
	LloydsPharmacy	Private sector healthcare providers	146,376.00	4	1
	University of Cardiff	Education and research providers	120,822.78	27	13
	Bluebay Medical Systems	Private companies other than providers of health services	116,900.00	26	1
	Hywel Dda University HB	Healthcare commissioning, planning and regulatory organisations	115,600.62	77	27
	National Assembly for Wales	Public administration and providers of public services	108,000.00	1	1
	Betsi Cadwaladr University HB	Healthcare commissioning, planning and regulatory organisations	101,352.58	76	21
	Cwm Taf University Health Board	Healthcare commissioning, planning and regulatory organisations	84,624.15	119	25
	Velindre NHS Trust	Public sector secondary and tertiary care providers	80,629.32	18	13
Northern Ireland	Belfast Health and SC Trust	Public sector secondary and tertiary care providers	60,615.65	30	17
	Federation Of Family Practices	Public sector primary care providers	40,235.20	3	3
	UK and Ireland Society of Cataract and Refractive Surgeons - Northern Ireland	Professional organisations	35,000.00	1	1
	Queen's University Belfast	Education and research providers	32,258.00	10	7
	Northern Health and SC Trust	Public sector secondary and tertiary care providers	23,703.46	14	8
	Medical Communications	Private companies other than providers of health services	23,250.00	3	1
	Adult ADHD - Northern Ireland	Patient organisation	20,000.00	1	1
	Ulster Chemists' Association	Professional organisations	16,584.00	4	3
	Western Health and SC Trust	Public sector secondary and tertiary care providers	15,068.55	12	10
	Cancer Focus Northern Ireland	Patient organisation	12,255.60	2	2

For peer review only - http://bmjopen.bmj.com/site/about/guidelines.xhtml

Supplementary File 9. Post-hoc Bonferroni pairwise comparisons between countries of payments per recipient type

Recipient type	Group 1 – Group 2*	Test Statistic	Std. Error	Std. Test Statistic	Sig.	Adj. Sig†,‡	Median (IQR) – group 1 - £	Median (IQR) – group 2 - £
Alternative providers of health services	England-Scotland	-59.003	22.681	-2.601	0.009	**0.028**	200 (160 – 394)	700 (360 – 1,100)
	England-Northern Ireland	-65.919	31.816	-2.072	0.038	0.115	200 (160 – 394)	600 (550 - 600)
	Scotland-Northern Ireland	6.917	38.645	0.179	0.858	1	700 (360 – 1,100)	600 (550 - 600)
Charities and other third-sector organisations	England-Scotland	-118.214	56.132	-2.106	0.035	0.106	223.52 (157 – 487.2)	**1700 (377.5 - 6,250)**
	Wales-England	79.844	27.702	2.882	0.004	**0.012**	120 (120 - 180)	223.52 (157 – 487.2)
	Wales-Scotland	198.059	62.049	3.192	0.001	**0.004**	120 (120 - 180)	**1700 (377.5 - 6,250)**
Education and research providers	England-Scotland	-5.623	27.857	-0.202	0.84	1	1000 (333.34 – 4,798.40)	1152 (400 – 2,880)
	England-Northern Ireland	-92.61	97.628	-0.949	0.343	1	1000 (333.34 – 4,798.40)	1100 (873.75 - 3525)
	Scotland-Northern Ireland	86.987	100.458	0.866	0.387	1	1152 (400 – 2,880)	1100 (873.75 - 3525)
	Wales-England	204.193	51.523	3.963	<.001	**0**	336 (175.2 - 1000)	1000 (333.34 – 4,798.40)
	Wales-Scotland	209.816	56.703	3.7	<.001	**0.001**	336 (175.2 - 1000)	1152 (400 – 2,880)
	Wales-Northern Ireland	296.803	109.409	2.713	0.007	**0.04**	336 (175.2 - 1000)	1100 (873.75 - 3525)
Formal bodies representing healthcare professionals	England-Scotland	-46.978	99.854	-0.47	0.638	1	200 (160 – 259.8)	213.6 (211.4 - 214.8)
	England-Northern Ireland	-223.228	99.854	-2.236	0.025	0.152	200 (160 – 259.8)	1066.67 (933.33 - 1200.00)
	Scotland-Northern Ireland	176.25	140.907	1.251	0.211	1	213.6 (211.4 - 214.8)	1066.67 (933.33 - 1200.00)
	Wales-England	138.133	27.905	4.95	<.001	**0**	120 (96 - 142)	200 (160 – 259.8)
	Wales-Scotland	185.111	103.261	1.793	0.073	0.438	120 (96 - 142)	213.6 (211.4 - 214.8)

For peer review only

	Wales-Northern Ireland	361.361	103.261	3.5	<.001	**0.003**	120 (96 - 142)	1066.67 (933.33 - 1200.00)
Healthcare commissioning, planning and regulatory organisations	England-Scotland	-122.159	44.623	-2.738	0.006	**0.037**	208.17 (160 – 307.2)	240 (131.18 - 500)
	England-Northern Ireland	-786.684	361.817	-2.174	0.03	0.178	208.17 (160 – 307.2)	1500 (470.8 - 4,087)
	Scotland-Northern Ireland	664.525	363.4	1.829	0.067	0.405	240 (131.18 - 500)	1500 (470.8 - 4,087)
	Wales-Scotland	94.65	56.651	1.671	0.095	0.569	225 (114.24 - 486.39)	240 (131.18 - 500)
	Wales-Northern Ireland	759.175	363.497	2.089	0.037	0.22	225 (114.24 - 486.39)	1500 (470.8 - 4,087)
	England-Wales	-27.51	45.405	-0.606	0.545	1	208.17 (160 – 307.2)	225 (114.24 - 486.39)
Patient organisation	Wales-England	144.727	77.321	1.872	0.061	0.367	747.93 (500 - 2,000)	**4000 (500 – 11,104)**
	Wales-Scotland	29.422	91.028	0.323	0.747	1	747.93 (500 - 2,000)	1000 (253.68 - 9,745)
	Northern Ireland-Wales	-8.633	118.686	-0.073	0.942	1	650 (600 - 1,450)	747.93 (500 - 2,000)
	Northern Ireland-Scotland	-38.055	103.121	-0.369	0.712	1	650 (600 - 1,450)	1000 (253.68 - 9,745)
	Northern Ireland-England	153.361	91.248	1.681	0.093	0.557	650 (600 - 1,450)	**4000 (500 – 11,104)**
	Scotland-England	115.306	50.259	2.294	0.022	0.131	1000 (253.68 - 9,745)	**4000 (500 – 11,104)**
Private companies other than providers of health services	England-Scotland	-317.041	89.112	-3.558	<.001	**0.002**	300 (196.8 – 598.92)	1,200 (350 - 5,528.88)
	England-Northern Ireland	-527.308	180.717	-2.918	0.004	**0.021**	300 (196.8 – 598.92)	**4179.38 (1,152.19 - 7,687.5)**
	Scotland-Northern Ireland	210.267	200.821	1.047	0.295	1	1,200 (350 - 5,528.88)	**4179.38 (1,152.19 - 7,687.5)**
	Wales-Scotland	115.061	106.256	1.083	0.279	1	**1475 (216 - 6,600)**	1,200 (350 - 5,528.88)
	Wales-Northern Ireland	325.327	189.758	1.714	0.086	0.519	**1475 (216 - 6,600)**	**4179.38 (1,152.19 - 7,687.5)**

For peer review only

	England-Wales	-201.98	60.166	-3.357	<.001	**0.005**	300 (196.8 – 598.92)	**1475 (216 - 6,600)**
Private sector healthcare providers	England-Scotland	-64.982	21.355	-3.043	0.002	**0.014**	240 (166 – 588)	647.54 (206.65 - 1,500)
	England-Wales	-88.107	39.755	-2.216	0.027	0.16	240 (166 – 588)	440 (360.94 - 1,732)
	Northern Ireland-Wales	-206.778	52.076	-3.971	<.001	**0**	38.49 (28.9 - 485)	440 (360.94 - 1,732)
	Northern Ireland-Scotland	-183.652	39.843	-4.609	<.001	**0**	38.49 (28.9 - 485)	647.54 (206.65 - 1,500)
	Northern Ireland-England	118.671	35.352	3.357	<.001	**0.005**	38.49 (28.9 - 485)	240 (166 – 588)
	Scotland-wales	-23.126	43.797	-0.528	0.597	1	647.54 (206.65 - 1,500)	440 (360.94 - 1,732)
Professional organisations	England-Northern Ireland	-35.169	109.4	-0.321	0.748	1	500 (240 – 3,200)	600 (320 - 1,784)
	Scotland-Northern Ireland	157.615	114.524	1.376	0.169	1	450 (285.67 - 980)	600 (320 - 1,784)
	Wales-England	182.447	69.008	2.644	0.008	**0.049**	400 (280 - 800)	500 (240 – 3,200)
	Wales-Scotland	60.001	76.873	0.781	0.435	1	400 (280 - 800)	450 (285.67 - 980)
	Wales-Northern Ireland	217.616	127.597	1.705	0.088	0.529	400 (280 - 800)	600 (320 - 1,784)
	Scotland-England	122.446	39.962	3.064	0.002	**0.013**	450 (285.67 - 980)	500 (240 – 3,200)
Public sector primary care providers	England-Northern Ireland	-355.779	76.292	-4.663	<.001	**0**	434.5 (193.6 – 869)	600 (434.5 - 1,600)
	Scotland-Northern Ireland	407.76	105.06	3.881	<.001	**0.001**	434.5 (202.23 - 651,75)	600 (434.5 - 1,600)
	England-Wales	-459.79	72.597	-6.333	<.001	**0**	434.5 (193.6 – 869)	800 (434.5 - 1,152)
	Northern Ireland-Wales	-104.011	102.62	-1.014	0.311	1	600 (434.5 - 1,600)	800 (434.5 - 1,152)
	Scotland-England	51.98	76.008	0.684	0.494	1	434.5 (202.23 - 651,75)	434.5 (193.6 – 869)
	Scotland-wales	-511.771	102.409	-4.997	<.001	**0**	434.5 (202.23 - 651,75)	800 (434.5 - 1,152)

*Each row tests the null hypothesis that the Sample 1 and Sample 2 distributions are the same.
†Asymptotic significances (2-sided tests) are displayed. The significance level is .050.
‡Significance values have been adjusted by the Bonferroni correction for multiple tests

For peer review only

Supplementary File 10. Post-hoc Bonferroni pairwise comparisons of payment types between countries

Payment Type*	Group 1 – Group 2**	Test Statistic	Std. Error	Std. Test Statistic	Sig.	Adj. Sig†,‡	Median (IQR) – group 1 - £	Median (IQR) – group 2 - £
Contribution to costs of Events	Wales-England	498.254	159.743	3.119	0.002	0.011	223.36 (120 - 400)	240 (155.95 - 400)
	Wales-Scotland	1449.82	208.45	6.955	<.001	0	223.36 (120 - 400)	300 (160 - 600)
	Wales-Northern	2690.485	402.839	6.679	<.001	0	223.36 (120 - 400)	477.6 (200 - 1,147.6)
	England-Scotland	-951.566	143.605	-6.626	<.001	0	240 (155.95 - 400)	300 (160 - 600)
	England-Northern	-2192.23	373.43	-5.871	<.001	0	240 (155.95 - 400)	477.6 (200 - 1,147.6)
	Scotland-Northern	1240.664	396.716	3.127	0.002	0.011	300 (160 - 600)	477.6 (200 - 1,147.6)
Donations and Grants	Wales-England	25.151	94.77	0.265	0.791	1	800 (434.5 - 2,200)	959.98 (256 - 4,800)
	Northern-Scotland	-140.935	137.745	-1.023	0.306	1	434.5 (217.5 - 1,867.5)	651.75 (217.25 - 2,578)
	Northern-Wales	-451.347	142.863	-3.159	0.002	0.009	434.5 (217.5 - 1,867.5)	800 (434.5 - 2,200)
	Northern-England	476.498	111.315	4.281	<.001	0	434.5 (217.5 - 1,867.5)	959.98 (256 - 4,800)
	Scotland-Wales	-310.412	124.755	-2.488	0.013	0.077	651.75 (217.25 - 2,578)	800 (434.5 - 2,200)
	Scotland-England	335.563	86.862	3.863	<.001	0.001	651.75 (217.25 - 2,578)	959.98 (256 - 4,800)

*Kruskal-Wallis results: Contributions to cost of events $\chi2(3) = 89.680$, p = .000; Donations and grants $\chi2(3) = 31.698$, p = <.000

**Each row tests the null hypothesis that the Sample 1 and Sample 2 distributions are the same.

†Asymptotic significances (2-sided tests) are displayed. The significance level is .050.

‡Significance values have been adjusted by the Bonferroni correction for multiple tests.

For peer review only

STROBE Statement—Checklist of items that should be included in reports of ***cross-sectional studies***

	Item No	Recommendation	Page No
**Title and abstract**	1	(*a*) Indicate the study's design with a commonly used term in the title or the abstract	2
		(*b*) Provide in the abstract an informative and balanced summary of what was done and what was found	2
**Introduction**			
Background/rationale	2	Explain the scientific background and rationale for the investigation being reported	3-4
Objectives	3	State specific objectives, including any prespecified hypotheses	4
**Methods**			
Study design	4	Present key elements of study design early in the paper	5
Setting	5	Describe the setting, locations, and relevant dates, including periods of recruitment, exposure, follow-up, and data collection	5
Participants	6	(*a*) Give the eligibility criteria, and the sources and methods of selection of participants	5
Variables	7	Clearly define all outcomes, exposures, predictors, potential confounders, and effect modifiers. Give diagnostic criteria, if applicable	5-6
Data sources/ measurement	8*	For each variable of interest, give sources of data and details of methods of assessment (measurement). Describe comparability of assessment methods if there is more than one group	5-6
Bias	9	Describe any efforts to address potential sources of bias	n/a
Study size	10	Explain how the study size was arrived at	N/a
Quantitative variables	11	Explain how quantitative variables were handled in the analyses. If applicable, describe which groupings were chosen and why	5
Statistical methods	12	(*a*) Describe all statistical methods, including those used to control for confounding	5
		(*b*) Describe any methods used to examine subgroups and interactions	n/a
		(*c*) Explain how missing data were addressed	n/a
		(*d*) If applicable, describe analytical methods taking account of sampling strategy	n/a
		(*e*) Describe any sensitivity analyses	n/a
**Results**			
Participants	13*	(a) Report numbers of individuals at each stage of study—eg numbers potentially eligible, examined for eligibility, confirmed eligible, included in the study, completing follow-up, and analysed	Supplementary File 4 (flow diagram)
		(b) Give reasons for non-participation at each stage	n/a
		(c) Consider use of a flow diagram	Supplementary File 4

For peer review only - http://bmjopen.bmj.com/site/about/guidelines.xhtml

Descriptive data	14*	(a) Give characteristics of study participants (eg demographic, clinical, social) and information on exposures and potential confounders	n/a
		(b) Indicate number of participants with missing data for each variable of interest	n/a
Outcome data	15*	Report numbers of outcome events or summary measures	6
Main results	16	(*a*) Give unadjusted estimates and, if applicable, confounder-adjusted estimates and their precision (eg, 95% confidence interval). Make clear which confounders were adjusted for and why they were included	n/a
		(*b*) Report category boundaries when continuous variables were categorized	n/a
		(*c*) If relevant, consider translating estimates of relative risk into absolute risk for a meaningful time period	n/a
Other analyses	17	Report other analyses done—eg analyses of subgroups and interactions, and sensitivity analyses	n/a
**Discussion**			
Key results	18	Summarise key results with reference to study objectives	11-13
Limitations	19	Discuss limitations of the study, taking into account sources of potential bias or imprecision. Discuss both direction and magnitude of any potential bias	13
Interpretation	20	Give a cautious overall interpretation of results considering objectives, limitations, multiplicity of analyses, results from similar studies, and other relevant evidence	11-14
Generalisability	21	Discuss the generalisability (external validity) of the study results	11-14
**Other information**			
Funding	22	Give the source of funding and the role of the funders for the present study and, if applicable, for the original study on which the present article is based	1

*Give information separately for exposed and unexposed groups.

Note: An Explanation and Elaboration article discusses each checklist item and gives methodological background and published examples of transparent reporting. The STROBE checklist is best used in conjunction with this article (freely available on the Web sites of PLoS Medicine at http://www.plosmedicine.org/, Annals of Internal Medicine at http://www.annals.org/, and Epidemiology at http://www.epidem.com/). Information on the STROBE Initiative is available at www.strobe-statement.org.

For peer review only

BMJ Open

Comparing pharmaceutical company payments in the four UK countries: a cross-sectional and social network analysis

Journal:	*BMJ Open*
Manuscript ID	bmjopen-2022-061591.R2
Article Type:	Original research
Date Submitted by the Author:	09-Mar-2023
Complete List of Authors:	Rickard, Emily; University of Bath, Department of Social and Policy Sciences<br>Carmel, Emma; University of Bath, Department of Social and Policy Sciences<br>Ozieranski, Piotr; University of Bath, Department of Social and Policy Sciences
**Primary Subject Heading**:	Health policy
Secondary Subject Heading:	Public health
Keywords:	Health policy < HEALTH SERVICES ADMINISTRATION & MANAGEMENT, MEDICAL ETHICS, PUBLIC HEALTH

SCHOLARONE™
Manuscripts

I, the Submitting Author has the right to grant and does grant on behalf of all authors of the Work (as defined in the below author licence), an exclusive licence and/or a non-exclusive licence for contributions from authors who are: i) UK Crown employees; ii) where BMJ has agreed a CC-BY licence shall apply, and/or iii) in accordance with the terms applicable for US Federal Government officers or employees acting as part of their official duties; on a worldwide, perpetual, irrevocable, royalty-free basis to BMJ Publishing Group Ltd ("BMJ") its licensees and where the relevant Journal is co-owned by BMJ to the co-owners of the Journal, to publish the Work in this journal and any other BMJ products and to exploit all rights, as set out in our licence.

The Submitting Author accepts and understands that any supply made under these terms is made by BMJ to the Submitting Author unless you are acting as an employee on behalf of your employer or a postgraduate student of an affiliated institution which is paying any applicable article publishing charge ("APC") for Open Access articles. Where the Submitting Author wishes to make the Work available on an Open Access basis (and intends to pay the relevant APC), the terms of reuse of such Open Access shall be governed by a Creative Commons licence – details of these licences and which Creative Commons licence will apply to this Work are set out in our licence referred to above.

Other than as permitted in any relevant BMJ Author's Self Archiving Policies, I confirm this Work has not been accepted for publication elsewhere, is not being considered for publication elsewhere and does not duplicate material already published. I confirm all authors consent to publication of this Work and authorise the granting of this licence.

For peer review only

Comparing pharmaceutical company payments in the four UK countries: a cross-sectional and social network analysis

Emily Rickard
Department of Social and Policy Sciences, University of Bath, Bath, United Kingdom.
Email: e.j.rickard@bath.ac.uk

Emma Carmel
Department of Social and Policy Sciences, University of Bath, Bath, United Kingdom.
Email: e.k.carmel@bath.ac.uk

Piotr Ozieranski (Corresponding Author)
Department of Social and Policy Sciences, University of Bath, Bath, United Kingdom.
Email: p.ozieranski@bath.ac.uk

Word count: 4921

Key words
Transparency, financial disclosures, pharmaceutical industry, health policy, conflicts of interest

ABSTRACT

Objectives To examine the characteristics of pharmaceutical payments to healthcare and patient organisations in the four UK countries. Compare companies spending the most; types of organisations receiving payments; and types of payments in the four countries. Measure the extent to which companies target payments at the same recipients in each country and whether it differs depending on the type of recipient.

Design Cross-sectional comparative and social network analysis.

Setting England, Scotland, Wales, Northern Ireland.

Participants 100 donors (pharmaceutical companies) reporting payments to 4,229 recipients (healthcare organisations and patient organisations) in 2015.

Main outcome measures For each country: payment totals and distribution; average number of common recipients between companies; share of payments to organisations fulfilling different roles in the health ecosystem; and payments for different activities.

Results Companies prioritised different types of recipient and different types of activity in each country. There were significant differences in the distribution of payments across the four countries, even for similar types of recipients. Recipients in England and Wales received smaller individual payments than in Scotland and Northern Ireland. Overall, targeting shared

For peer review only - http://bmjopen.bmj.com/site/about/guidelines.xhtml

recipients occurred most frequently in England, but was also common in certain pockets of each country's health ecosystem. We found evidence of reporting errors in Disclosure UK.

Conclusions Our findings suggest a strategic approach to payments tailored to countries' policy and decision-making context, indicating there may be specific vulnerabilities to financial conflicts of interest at sub-national level. Payment differences between countries may be occurring in other countries, particularly those with decentralised health systems and/or high levels of independence across its decision-making authorities. We call for a single database containing all recipient types, full location details, and published with associated descriptive and network statistics.

STRENGTHS AND LIMITATIONS OF THIS STUDY

- This is the first study to compare pharmaceutical industry payments in England, Scotland, Wales and Northern Ireland
- Our analysis created a new database combining payments disclosed in Disclosure UK with individual company disclosures of payments to patient organisations
- We use social network analysis to facilitate a systematic sub-national comparison of payments
- One key limitation is that the data is from 2015 and is not able to assess trends in payment types or amounts.

For peer review only

INTRODUCTION

Some of the major pharmaceutical companies spend more on marketing than on the development of products[1 2 3]. Industry marketing efforts include payments to physicians, which are seen to boost innovation and efficiency in healthcare[4] but also generate concerns about *individual* financial conflicts of interest (COIs), influencing prescribing choices[5] and leading to patient harms[6]. Payments to healthcare and patient organisations have also been seen to generate *institutional* financial COIs around policy and programme decision-making. An institution's primary goals *may* be unduly influenced by a secondary interest[7], which can be more damaging than individual COIs[7-9]. COIs are defined in terms of the *risk* of undue influence and not actual bias or misconduct[9], but institutional COIs have been linked to increased prescriptions of drugs with unproven safety[8], distorting research agendas[10], threatening the objectivity of professional education[7], and compromising independence[11]. These observations have highlighted what has been called the pharmaceutical industry's 'web of influence', in which companies "sustain large networks to gather, create, control and disseminate information"[12].

The potential to distort public health research and policy to favour commercial interests above patients' has led to increased policy scrutiny[13 14], including the introduction of self-regulatory payment disclosure requirements for pharmaceutical companies in Europe[15]. Such measures are intended to aid transparency, reducing conflicts of interest and undue influence on clinical and policy decisions. This article combines and analyses disclosure data to better understand the depth, breadth, and structure of industry payments and compares them in the four countries of the UK. Comparative analysis can illustrate novel ethical and governance problems[16] or reveal that recognised problems are common across countries[17], which our systematic examination of the extent and diversity of payments reported by pharmaceutical companies explores.

Disclosure of industry payments

In the United States, pharmaceutical industry disclosures of payments to physicians and teaching hospitals were made mandatory in 2013[18], and subsequent research has examined payments to physicians[19-30], with institutional payments to hospitals largely ignored[31]. Payments to patient organisations, defined as not-for-profit institutions that primarily represent the needs of patients and/or caregivers[32], have been seldom explored in the US[33 34] as their disclosure is not regulated by the state or industry.

The prevalence of self-regulation in Europe is associated with very different disclosure rules to the US[35 36]. Since 2012, the European trade association, the European Federation of Pharmaceutical Industries and Associations (EFPIA), has mandated that pharmaceutical companies publish annual disclosures of their payments to patient organisations on their websites[37]. Subsequent studies revealed extensive funding in the UK[38 39] and the Nordic countries[40 41]. However, transparency remains limited by a lack of standardised reporting requirements and limited oversight[42] which are associated with payment under-reporting by both donors and recipients[39].

In separate self-regulatory arrangements[43], disclosures of payments to healthcare organisations, defined by the industry as healthcare, medical or scientific associations or organisations such as hospitals, clinics, foundations or universities[44], have been mandated

For peer review only - http://bmjopen.bmj.com/site/about/guidelines.xhtml

since 2015. In the UK, these are reported annually in a centralised database, Disclosure UK, hosted by the industry trade body, the Association for the British Pharmaceutical Industry (ABPI). Most research attention has been on the poor accessibility and quality of the data[35], noting lack of standardisation of recipients[4 25] and inadequate details about individual payments' purpose[4]. These make tracking and analysing the payments complicated and time-consuming, hindering the principle aim of improving transparency.

Our study is the first, to our knowledge, to systematically combine industry data on payments to healthcare organisations and patient organisations as the self-regulatory codes allow them to be reported separately. Analysing them together enables us to better assess the breadth and depth of the 'web of influence', and gain insight into potential reinforcement effects of payments to multiple and diverse organisations that have separate yet overlapping interests, including providing patient care and support[31 38], involvement in policy-making[13 45 46], and conducting clinical research[9 38].

Regional differences in industry payments

Another aspect of the industry's web of influence largely unexplored in Europe is whether and how it is structured around regional differences in payments. Little is known about strategic targeting of particular fields of healthcare provision and/or decision-making, nor about possible effects on COIs in regional policy-making. Regionally targeted payments may have direct policy effects 'upstream', such as commissioning (the planning, prioritising, and purchasing of public health services)[47]; and 'downstream', such as bearing greater influence on organisational priorities and day-to-day practices.

Emerging US research has found significant differences in the distribution of payments between states[20 48-50], including by state size[51] and political composition[52], indicating that demographics and the organisation and regulation of healthcare matter. The first regional analysis in Europe revealed differences in the total value and type of payments prioritised in eight countries[17]. Most recently a UK study found headquarter distance from country capitals predicts patient organisations' dependence on pharmaceutical company funding[53]. To date, research has not considered the locations of patient and healthcare organisations as the reporting requirements do not extend to disclosing country locations[17 25 28].

However, the UK presents a crucial case for this type of analysis given its importance as a pharmaceutical market[54], large value of payments compared to other European countries[17] and vast charitable sector comprising many potential recipients[55]. England, Scotland, Wales and Northern Ireland have four distinct health systems, with substantial autonomy to determine health policies and services[56-58]. They also differ demographically – population size is largest in England and smallest in Northern Ireland[59] and health outcomes are highest in England and lowest in Scotland[60]. The demographic and health system differences could be associated with how industry engages with different healthcare sectors.

We know that pharmaceutical companies prioritise payments to different types of healthcare organisations in the UK[25 28], however commercially patterned inequalities, including dominant funders or types of recipients, may be more pronounced sub-nationally in the in the smallest UK countries yet hidden by UK-level analysis to date[16]. Studies have started recognising the country distinction, focusing on payments to healthcare

For peer review only - http://bmjopen.bmj.com/site/about/guidelines.xhtml

organisations in England[47 61], but cross-country comparisons have not yet been conducted. Comparative insights could also help understand whether similar patterns are occurring in other European countries with highly decentralised healthcare set-ups, including Germany[62] and Spain[63].

In this article, we apply social network analysis (SNA) which offers new insights into industry marketing tactics[64 65 61]. SNA can reveal areas of the healthcare ecosystem where connections between companies, measured by the number of payment recipients companies have in common, are most prevalent. Prevalent connections may highlight industry marketing efforts in pockets of each of the UK's health systems, including indicating areas of *competition* between companies[66 67 68] and revealing areas where companies are seeking to enhance their visibility[61 69].

We integrate and analyse data from Disclosure UK and disclosures of payments to patient organisations to examine patterns in pharmaceutical company payments to organisations in the UK healthcare ecosystem. Specifically, our objectives are to:

- examine the characteristics of payments to healthcare and patient organisations in the four countries
- compare the top donors financially in each country
- identify similarities and differences in the types of payments and in the types of organisations receiving payments in the four countries
- measure the extent to which companies target payments at the same recipients in each country and whether it differs depending on the type of recipient.

METHODS

Data sources

Our primary data sources are publicly available pharmaceutical industry transparency disclosures from 2015. Corresponding to relevant ABPI[70] and EFPIA Codes[37], pharmaceutical companies disclose payments to healthcare organisations and to patient organisations separately.

Payments to healthcare organisations are disclosed in a centralised database, Disclosure UK, published annually by the ABPI. Payments are disclosed with recipient name, payment type (donations and grants, costs of events, joint working, and consultancy – see Supplementary File 1) and value, and address information. We use the 2015 version of Disclosure UK and focus on non-R&D payments to healthcare organisations (R&D payments are reported as one lump sum per company without named recipients[25 43]).

Payments to patient organisations are only available on individual pharmaceutical company websites and are usually presented as a PDF file and include recipient name, payment description, and payment value[42]. We extracted the payments to patient organisations data into a single database, standardising names and identifying headquarter addresses as part of another project[38]. We detail our approach to data cleaning these data elsewhere[25 38].

For peer review only - http://bmjopen.bmj.com/site/about/guidelines.xhtml

Dataset preparation and integration

We followed several steps to prepare the Disclosure UK and patient organisation datasets for analysis. First, we merged the two datasets (see Supplementary File 2 for data integration flowchart). Second, as Disclosure UK provides incomplete addresses, we conducted independent web searches on each payment recipient to determine which UK country they are based. We used the same methodology to determine patient organisations' locations. Third, we excluded payments to patient organisations duplicated in the two datasets and identified patient organisations incorrectly reported as healthcare organisations in Disclosure UK. Fourth, we coded the patient organisation descriptions to match the codes used by Disclosure UK (Supplementary File 1).

Fifth, as part of a previous study[25] we standardised recipient names for almost 20,000 payment entries and inductively categorised them based on their function within the healthcare system (e.g. service provider) and their sector (e.g. public or private) (see Supplementary File 3 for comprehensive definitions and examples of organisations). For the current study we introduced patient organisations. Recipient types (with the most frequently occurring example) included in our analysis are:

Providers of health services

- Alternative providers of health services (e.g. community interest companies providing health services)
- Healthcare commissioning, planning and regulatory organisations (e.g. clinical commissioning groups)
- Private sector healthcare providers (e.g. private healthcare groups)
- Public sector primary care providers (e.g. GP surgeries)
- Public sector secondary and tertiary care providers (e.g. NHS trusts)

Representative organisations

- Formal bodies representing healthcare professionals or patients (e.g. local medical committees)
- Patient organisations (e.g. multipurpose patient organisations)
- Professional organisations (e.g. multi-professional or multi-stakeholder organisations)

Other organisations

- Charities and other third-sector organisations (excludes providers of health services, professional organisations, and patient organisations) (e.g. charitable trusts providing educational events for healthcare professionals)
- Education and research providers (e.g. universities)
- Private companies other than providers of health services (e.g. providers of medical communications or training services)
- Public administration and providers of public services (e.g. local authorities)
- Recipients unclear (when no information could be found)

Analysis

We calculated the total and median value of payments in each country and recipient type. The Shapiro-Wilks test of normalcy found the data to be non-normal in each country,

For peer review only - http://bmjopen.bmj.com/site/about/guidelines.xhtml

therefore non-parametric Kruskal-Wallis tests (adjusted for ties) were used to check for between-country differences in the distribution of payments overall and in the different recipient types. Dunn's post-hoc pairwise analyses (with Bonferroni's correction for multiple comparisons) were conducted to identify where differences were present between countries and recipient types. Kruskal-Wallis and Dunn's tests do not assume equal sample sizes[71] and have been conducted on similar industry disclosure data[72 73 74]. Statistical significance was set at p = ≤.05.

SNA was used to measure the number payment recipients that were common between pairs of pharmaceutical companies (density) and across all companies (degree centrality). Density measures the overall level of connection in a network and can be used to compare the structure of different groups[75]. It produces two outputs – average value (average number of recipients each pair of companies shares[76]) and average weighted degree (average of the total number recipients each company shares with other companies). The higher these values, the more frequently a multiple companies target the same recipients in a given network[77]. For example, a density score of 1.194 tells us that all pairs of companies in the network funded an average of 1.2 recipients in common. Degree centrality, on the other hand, provides a score for each individual company based on the number of recipients in common it shares with other companies in the network – the higher the score, the more recipients a company shares[75 78]. For example, if a company has a degree centrality score of 320, they funded the same recipient as another company 320 times.

We compare the number of common recipients companies have in each country overall and when targeting different recipient types in each country. SNA requires data to be structured as a matrix, therefore we transformed the payment data into a series of matrices of pharmaceutical companies with ties based on the number of recipients each company shared with other companies in each country and recipient type. To identify which companies targeted the same recipients, each matrix consisted only of the companies making at least one payment (regardless of whether or not they shared any recipients). We conducted separate network analyses on each of the four countries as the findings would otherwise be highly influenced by England's data as the largest network.

Data was processed in Microsoft Excel. The dataset underpinning our analysis is published in the Bath Research Data Archive[79]. We analysed the data descriptively in SPSS version 27 (IBM) and Microsoft Excel. We conducted social network analysis in UCINET version 6[77]. Country networks were visualised in Gephi version 0.9.2.

Outcome measures

Breadth of payments in each country
First, we explored the payment characteristics in each country. We measured the total and median values and the number of: payments, recipients, and companies. We adjusted the total value by population size for comparison. We also compared the distribution of payments between each country using Kruskal-Wallis tests.

Second, we identified the top 10% of companies making payments in each country and compared the payment strategies of the companies paying the most in each country.

For peer review only - http://bmjopen.bmj.com/site/about/guidelines.xhtml

Depth of payments in each country

Third, we assessed companies making payments to the same recipients by measuring the average number of common recipients between each pair of companies (degree centrality).

Fourth, we scrutinised which companies dominate the payment networks in each country by identifying the number of recipients that each company had in common with every other company.

Structure of payments in each country

Fifth, for each country we identified which type of recipient was prioritised. To do this, we measured and compared the proportion of payments received by each recipient type. We also compared the distribution of payments to each recipient type using Kruskal-Wallis tests to determine whether payments to similar types of recipients differ between countries.

Sixth, we examined whether companies making payments to *each recipient type in each country* made payments to the same organisations by measuring the average number of recipients each pair of companies share.

Seventh, for each country we assessed which types of payments were prioritised through identifying the proportion of different payment types. We also compared the four types of payments using Kruskal-Wallis tests to identify differences in the distribution of payments.

Disclosure accuracy

Finally, as a secondary outcome we measured the number of patient organisations, alongside the number and value of payments, that were incorrectly disclosed as healthcare organisations in Disclosure UK.

Patient and public involvement

The study did not involve patients.

RESULTS

We structure our findings consistent with the order of the outcome measures outlined above. First, we explore the breadth, depth, and structure of payments in each country. While there is inevitable overlap between these framing terms, this will be signposted throughout. We also examine overall accuracy of disclosures.

Breadth of payments in the four countries

The total value and number of payments, the number of recipients, and the number of companies making payments were consistent with the size of each country, with England receiving the highest and Northern Ireland the lowest (this was maintained after adjusting for population size – see Table 1).

Table 1. Value and number of payments, number of companies and recipients, and top donors in integrated dataset

Descriptive statistic	England	Scotland	Wales	Northern Ireland

For peer review only - http://bmjopen.bmj.com/site/about/guidelines.xhtml

Country population 2015 - n*	54,786,300	5,373,000	3,099,100	1,851,600
Total value - £	52,445,615	3,649,749	1,987,703	518,000
Total value - £ (adjusted for population size)†	957,037	675,880	641,194	272,632
Payments - n	18,190	1,370	990	311
Recipients - n	3,575	282	216	156
Companies – n	100	72	64	42
Median payment value (IQR) - £	280 (665.5)	400 (685.3)	300 (658.2)	475.20 (1,164.4)
Value of payments to healthcare organisations - £	40,217,772	3,029,365	1,887,918	474,795
Value of payments to patient organisations - £	12,227,843	620,384	99,784	43,206

*Data obtained from the Office for National Statistics, values correct for mid-2015
†Total value of payments divided by the population size

Between-country differences in payment values

There was a statistically significant difference in the distribution of individual payments between the four countries, $\chi2(3) = 50.127$, p = <.001. Dunn's post-hoc comparisons showed that this difference was driven by significantly higher median payments (Table 1) being made in Scotland (p - <.001) and Northern Ireland (p = <.001) than England. Payment size also varied significantly between Northern Ireland-Wales (p = <.000), Scotland-Wales (p = .001), and Northern Ireland-Scotland (p = .004).

Top donors in each country

The companies spending most in each country also reveals different approaches to payments (see Supplementary File 4). The top donors generally made larger payments in Wales and multiple smaller payments in Northern Ireland. Pfizer was consistently a top donor measured by value and volume of payments in all four countries, indicating an approach to payments focused on breadth. At the country-level, in England, Novartis was the second biggest donor characterised by large payments; similar patterns characterised Biogen's payments in Scotland and Wales. England, Scotland and Northern Ireland all had at least one top donor not featuring as a top donor in another country, indicating some companies' payments may be more targeted regionally than others.

Depth of engagement in the four countries

Companies making payments in England had the highest number of common recipients - an average of six to seven recipients (Table 2), implying a significant concentration of shared interest around a spectrum of organisations. Companies, on average, had at least one recipient in common with another company in Scotland and Wales, and were least connected in Northern Ireland (Table 2), indicating that in smaller countries, company interest in particular recipients is more concentrated. The average weighted degree density score shows the average number of recipients a company shares with *all* companies in the

For peer review only

network, where similarly the highest score was observed in England (664.36 recipients) and lowest in Northern Ireland. The visualised networks are in Supplementary File 5.

Table 2. Pharmaceutical company connections in each country measured by common recipients

Network measure*	England	Scotland	Wales	Northern Ireland
Density – average value (average number of recipients in common between two companies)	6.71	1.24	1.13	0.42
Density – average weighted degree (average number of recipients in common for all companies in the network)	664.36	88.39	71.06	17.38
Company with highest degree centrality score (number of recipients a company has in common with all other companies in the network)	Pfizer (3,394)	Pfizer (319)	Pfizer (206)	Pfizer (63)

*Calculations were conducted on valued networks which means they consider the number of common recipients and not just the presence of a shared recipient. Networks include only companies making payments in each country.

The data also indicates variation in the depth of payments at the company level, as some companies focus collectively on particular recipients and some companies target a broader set of organisations with exclusive funding. Pfizer consistently targeted the same recipients as other companies most frequently in every country. Pfizer's degree centrality score of 3,394 in England shows that the company funded the same organisation as another company 3,394 times in the year (Table 2). Many of the most connected companies (see Supplementary File 6) were similar in England, Scotland and Wales. However, Northern Ireland's top ten most connected companies were more varied and featured smaller companies, suggesting that a cluster of companies had a unique interest Northern Ireland's health system. Further, differences between top donors and topmost connected companies in each country highlight potentially divergent strategies in targeted funding. For example, Merck Sharpe and Dohme was highly connected in every country but was not a top donor.

Coupled, the SNA and descriptive data provides evidence that some companies prioritise breadth of payments, targeting a broader spectrum of organisations, while other companies prioritise depth, targeting recipients which seem important or 'popular' across the industry and potentially competing with other companies for visibility.

Structure of payments in each country

Structural differences in targeted recipient types between countries

The share of the total value of payments received by recipient types revealed diverse funding strategies in each country (Figure 1). In Wales and Scotland, industry targeted funding 'upstream' at healthcare commissioning, planning and regulatory organisations, primarily each country's local health boards that plan and deliver NHS services[80 81]. In Wales, they received just under half of all payments - £920,980.22 (46.38% of Wales' total payments, see Supplementary File 7 for values and Supplementary File 8 for top recipients).

For peer review only - http://bmjopen.bmj.com/site/about/guidelines.xhtml

In Scotland, they received £878,333.57 (24.13%). Notably, the two Scottish health boards serving the fewest people received no payments. In England and Northern Ireland, funding was targeted ‘downstream’. England’s public sector secondary and tertiary care providers, namely consisting of NHS trusts which provide hospital and sometimes community healthcare services to residents[82], received the most funding (£13,349,779.1 – 25.56%). In Northern Ireland, public sector primary care providers, primarily general practitioner practices, were targeted with the most funding (£184,903.09 **–** 35.72%).

Figure 1. Percentage of payments to recipient types per country

There were statistically significant differences in the distribution of payments, indicating that payment values vary between the four countries even when the recipient type is the same (see Table 3). Post-hoc analyses maintained the significant differences, except for in patient organisations (see Supplementary File 9).

Patient organisations were a major target of payments, especially in England and Scotland (Table 3). Professional organisations, including societies and groups of healthcare professionals, were prioritised in England, Scotland and Northern Ireland, with significant but negligible differences in payment values. Consistent with the locations of the top UK universities, industry targeted education and research providers in England (median = £1000) and Scotland, (£1,152) where payments were also significantly higher than Wales (£336). Public sector primary care providers, primarily general practitioner practices, received a very small proportion of the total funding in England and Scotland, yet had the most individual recipients in all four countries, suggesting smaller per-recipient payment totals. This is further reflected in the median values per recipient, which were significantly lower in England (£435) and Scotland (£435) than Wales (£800) and Northern Ireland (£600).

Table 3. Differences in payment sizes between countries

**Recipient type**	**Median (IQR) - £**				***p* value**
	England	Scotland	Wales	Northern Ireland	
Alternative providers of health services	200 (160 – 394)	700 (360 – 1,100)	n/a	600 (550 - 600)	0.004*
Charities and other third-sector organisations†	223.52 (157 – 487.2)	1700 (377.5 - 6,250)	120 (120 - 180)	n/a	0.001*
Education and research providers	1000 (333.34 – 4,798.40)	1152 (400 – 2,880)	336 (175.2 - 1000)	1100 (873.75 - 3525)	0.001*
Formal bodies representing healthcare professionals	200 (160 – 259.8)	213.6 (211.4 - 214.8)	120 (96 - 142)	1066.67 (933.33 - 1200.00)	<.000*
Healthcare commissioning, planning and regulatory organisations	208.17 (160 – 307.2)	240 (131.18 - 500)	225 (114.24 - 486.39)	1500 (470.8 - 4,087)	0.008*
Patient organisations	4000 (500 – 11,104)	1000 (253.68 - 9,745)	747.93 (500 - 2,000)	650 (600 - 1,450)	0.011*
Private companies other than providers of health services	300 (196.8 – 598.92)	1,200 (350 - 5,528.88)	1475 (216 - 6,600)	4179.38 (1,152.19 - 7,687.5)	<.000*

For peer review only - http://bmjopen.bmj.com/site/about/guidelines.xhtml

Private sector healthcare providers	240 (166 – 588)	647.54 (206.65 - 1,500)	440 (360.94 - 1,732)	38.49 (28.9 - 485)	<.000*
Professional organisations	500 (240 – 3,200)	450 (285.67 - 980)	400 (280 - 800)	600 (320 - 1,784)	0.001*
Public administration and providers of public services	394.4 (224.45 – 546.67)	540 (200 - 600)	n/a	n/a	0.238
Public sector primary care providers	434.5 (193.6 – 869)	434.5 (202.23 - 651,75)	800 (434.5 - 1,152)	600 (434.5 - 1,600)	<.000*
Public sector secondary and tertiary care providers	233.17 (141.87 – 500)	300 (189.75 - 612)	253.66 (200 - 954)	288 (163.4 - 490.13)	0.055

*Statistically significant
†Excluding providers of health services, professional organisations and patient organisations

Extent of company connections in targeted recipient types in each country

Companies shared 5.8 common recipients on average among England's public sector secondary and tertiary care providers (Table 4), which also received the most funding. These patterns could be a function of the number of research-active NHS trusts located in England[83], meaning service providers might be very effective at getting donor funds, but also suggest a high degree of targeting by industry. Notably, although healthcare commissioning, planning and regulatory organisations, primarily clinical commissioning groups responsible for the planning and purchasing of local health care services[84], received very little funding in England, companies frequently target the same recipients, indicating that low funding does not infer an absence of interest.

In Scotland and Wales, companies targeted the same healthcare commissioning, planning and regulatory organisations most frequently, consistent with the financial prioritisation. In Northern Ireland, the density score for public sector primary care providers was higher than the other countries, suggesting some companies have overlapping interests in specific recipients in pockets of Northern Ireland's primary care system. In Wales, Scotland and Northern Ireland in particular, these patterns of common recipients pose a potentially greater risk to certain areas of the healthcare ecosystem becoming vulnerable to influence given the much smaller population the organisations serve.

Table 4. Density scores for valued recipient type networks in each country

**Recipient type**	**Density scores***			
	England	Scotland	Wales	Northern Ireland
Alternative providers of health services	0.339†	0.500	-	0.000
Charities and other third-sector organisations	0.510	0.333	0.476	-
Education and research providers	1.194	0.727	0.675	1.000
Formal bodies representing healthcare professionals	1.293	0.000	0.400	0.000
Healthcare commissioning, planning and regulatory organisations	2.523	1.578	1.634	0.133

For peer review only

Patient organisations	0.337	0.200	0.109	0.209
Private companies other than providers of health services	0.312	0.121	0.071	0.000
Private sector healthcare providers	0.416	0.167	0.167	0.067
Professional organisations	0.611	0.244	0.114	0.038
Public administration and providers of public services	0.022	0.300	0.000	-
Public sector primary care providers	0.893	0.038	0.124	1.600
Public sector secondary and tertiary care providers	5.819	1.309	1.000	0.826

* Density scores measure the average number of common recipients between two companies. The network matrix for each recipient type consisted only of companies making payments. Dashes indicate no payments were made. Scores of 0.000 indicate all recipients received payments from one company only.
†Example interpretation: a score of 0.339 indicates that each company making payments to alternative providers of health services funded, on average, 0.3 recipients in common with another company.

Prioritised payment types in each country

Another dimension of structure that differed between countries was the type of payments (Figure 2). Donations and grants, such as medical and educational goods, were consistently prioritised, however there was notable diversity between countries among the remaining payment types. Payments for joint working, defined as initiatives involving shared investment by the NHS and pharmaceutical companies[85], varied from 19.61% of all payments in Wales to 2.29% in Northern Ireland; fees for service and consultancy varied from 33.78% in Scotland to 4.86% in Northern Ireland; and contributions to costs of events, such as science or medical focused conferences and educational events, ranged from 31.87% in Northern Ireland to 18.58% in Wales.

Figure 2. Percentage of total value for each payment type

There was a statistically significant difference between the distribution of payments for costs of events (p = .000), which were lowest in Wales (£223) and highest in Northern Ireland (£478), and donations and grants (p = <.000), which were lowest in Northern Ireland (£435) and highest in England (£960). Differences in fees for service and consultancy (p = .995) and joint working (p = .261) were non-significant (see Supplementary File 10).

Accuracy of disclosures

We found evidence of pharmaceutical companies misinterpreting disclosure requirements when we integrated the Disclosure UK and patient organisation data (see Supplementary File 3 for data integration flowchart). We identified 341 payments (1.71% of all payments to organisations in Disclosure UK) to 116 patient organisations (2.88% of all organisations in Disclosure UK) worth £2,458,931.99 (5.21% of the total) incorrectly disclosed as healthcare organisations in Disclosure UK. Of these payments, 50 (14.66%) were duplicated in the

For peer review only - http://bmjopen.bmj.com/site/about/guidelines.xhtml

patient organisation and Disclosure UK data, which were excluded to ensure no payment was counted twice.

DISCUSSION

Principle findings

Our findings offer insights into the pharmaceutical industry's strategic approach to payments tailored to the policy and decision-making context between, and even within, each country. Our findings also indicate that the pharmaceutical industry's 'web of influence'[14] can be relatively structured and aligned with key within-country differences in health system design and processes, as well as cross-nationally. Our comparative analysis illustrates novel ethical and governance problems as well as commonalities across countries and confirms concerns that UK-level analysis[25 38] obscures important regional payment variations and recipient vulnerabilities[16]. The oversight of strategic specificity is important not least because key decisions about commissioning of health services are taken within each country[47 61].

Findings in context

Our findings align with previous comparative analyses of payments to teaching hospitals[31] and healthcare professionals in the United States, which show significant payment differences between regions[20 48 49 86]. Our findings also mirror those from a comparative study of industry payments to patient organisations in Denmark and Sweden, where larger payments were more frequent in the smallest country[16], suggesting a consistent industry strategy of targeting smaller locations with larger payments.

The concentration of payments among a few companies in each country was also consistent with previous studies of patient organisations[16 38 87] and healthcare organisations[25 31 61]. We identified Pfizer as a top donor, targeting many 'popular' recipients in all four UK countries, however it remains unclear if this relates to a particular product launch[40 88], a new push relative to emerging competition, or reflects a consistent trend. Further interpretation would be facilitated by longitudinal analysis. There were also differences in the companies providing the most funding, particularly in Northern Ireland where the top donors were similar to those making payments to healthcare organisations in the Republic of Ireland[28] rather than the other three UK countries, indicating that some companies may strategically target organisations on the island. One isolated case was Napp Pharmaceuticals, which featured as both a top donor and top-most connected company uniquely in Northern Ireland, suggesting that specific companies can dominate payment networks in relation to smaller countries under the radar. These instances may have direct implications for public health. For example, Napp Pharmaceuticals is an opioid manufacturer[89] and opioid manufacturers in the United States have been known to leverage targeted funding, including to teaching hospitals[31], to increase opioid prescribing[90].

Discrepancies in the types of payments prioritised also point towards sub-national vulnerabilities in each countries' healthcare ecosystem. In Wales, the prioritisation of joint working raises concerns around the extent of pharmaceutical industry involvement in healthcare design. Joint working arrangements are intended to bring benefits to patients, the NHS, and companies, however many of these projects mention increasing the use of

For peer review only - http://bmjopen.bmj.com/site/about/guidelines.xhtml

company products[91], potentially serving as an alternative avenue for industry marketing. Similarly, in Northern Ireland, costs of events were higher than the other countries, pointing towards an alternative channel for industry involvement in continuing medical education in a country with fewer professional organisations or large universities. This pattern of frequent event payments was also observed in the Republic of Ireland[28], further indicating island-specific trends.

Lessons for transparency

The transparency concerns we identified are consistent with previous studies of pharmaceutical industry disclosure practices in the UK[4 42] and Europe[35 41]. Although the UK's self-regulatory payment disclosure system is the most robust in Europe[17 92], our analysis confirms earlier concerns about some payments being disclosed on the incorrect platform and thereby preventing their correct identification by policymakers, regulators, and members of the public[39 42]. Our findings indicate that some instances of under-reporting[39] may be explained by confusion about where to report.

These issues, coupled with the extensive additional research required to standardise and categorise recipient types and their locations in the UK, indicate that the self-regulatory system is incomplete and requires better integration. This could be achieved through a single standardised database comprising all pharmaceutical industry payments and combining the highest standards of reporting as they currently apply to, separately, healthcare and patient organisations. For example, EFPIA requires individual disclosures of payments to patient organisations to include descriptions of funded activities[42], a provision that should be extended to healthcare organisations. As a minimum, compulsory recipient identifiers should be introduced[35] to reduce the substantial forensic work involved in cleaning these data and encourage longitudinal comparisons. Echoing calls in the US for state-specific disclosure policies[51], Disclosure UK and disclosures of payments to patient organisations need to be adapted to better capture the distinction between payments in England, Scotland, Wales, and Northern Ireland.

Whilst baseline improvements in data accessibility and quality are imperative, a central database should also contain associated analytics, including descriptive and network statistics. Otherwise, we run the risk that pharmaceutical companies themselves gain more from the payment disclosure system than the public, as companies use disclosures to inform and fine-tune their marketing efforts[93 67].

Strengths and limitations

This is the first study to jointly analyse payments to healthcare and patient organisations, which was made possible by the current UK transparency provisions. It also is the first of its kind to explore payments across the four UK countries. To date, the spotlight has been on individual COIs, which may downplay the systemic problem of a broader institutional culture whereby industry funding is embraced and industry interests can be advanced[14 90]. However, our study has limitations. We focus only on 2015 data due to the substantial time required to prepare Disclosure UK data for effective analysis, particularly categorising recipients to make them distinguishable, and identifying recipient countries. We can assume the patterns are maintained over time as the overall payment values have remained stable each year[38 94], however longitudinal analysis would confirm this. Also, we could not

For peer review only - http://bmjopen.bmj.com/site/about/guidelines.xhtml

determine whether sharing recipients was accidental or intentional, nor did we measure the impact of these payments.

Conclusion

Regional variability in payments has implications for sub-national policymaking[51] and it appears that there are specific vulnerabilities to institutional COIs arising at a sub-national level. These payment differences may also be occurring in other countries, particularly those with decentralised health system structures and/or high levels of independence across their decision-making authorities. Future research could examine factors contributing to regional payment differences to better inform future government or industry policies to mitigate against undue influence.

For peer review only

For peer review only - http://bmjopen.bmj.com/site/about/guidelines.xhtml

Ethical approval

The study did not require ethical approval (as it draws on publicly available data at the organisational level), however it is part of a bigger project which has ethical approval from the University of Bath's Social Sciences Research Ethics Committee (approval code: S19-073).

Funding statement

ER's work was supported by a +3 PhD Studentship award match-funded (50%) by the Economic and Social Research Council and the University of Bath. This work forms part of the PhD project. PO's work was supported by grants from The Swedish Research Council for Health, Working Life and Welfare (FORTE), no. 2016-00875, and The Swedish Research Council (VR), no. 2020-01822.

Competing interests statement

ER and EC have no conflicts of interests to declare. PO's PhD student was supported by a grant from Sigma Pharmaceuticals, a UK pharmacy wholesaler and distributor (not a pharmaceutical company). The PhD work funded by Sigma Pharmaceuticals is unrelated to the subject of this paper.

Author contributions

ER designed, managed, analysed and interpreted the data, as well as drafted the article. PO conceived and designed the study, provided supervision, and drafted the article. EC co-supervised the analysis and contributed to drafting the article.

Data availability statement

All data relevant to the study are included in the article or uploaded as supplementary information. The authors of this study agree to share data underpinning this study in the form of an Excel database available from the University of Bath Research Data Archive. The raw data poses no risk to anonymity of individuals as it draws on publicly available reports concerning financial transfers between organisations. The reference for this dataset is: Rickard, E., Ozieranski, P., 2023. *Dataset for "Comparing pharmaceutical company payments in the four UK countries: a cross-sectional and social network analysis"*. Bath: University of Bath Research Data Archive. https://doi.org/10.15125/BATH-01239.

For peer review only

REFERENCES

1. Lexchin J. Pharmaceutical company spending on research and development and promotion in Canada, 2013-2016: a cohort analysis. *J Pharm Policy Pract* 2018;11:5-5. doi: 10.1186/s40545-018-0132-3
2. AHIP. New Study: In the Midst of COVID-19 Crisis, 7 out of 10 Big Pharma Companies Spent More on Sales and Marketing than R&D 2021 [Available from: https://www.ahip.org/news/articles/new-study-in-the-midst-of-covid-19-crisis-7-out-of-10-big-pharma-companies-spent-more-on-sales-and-marketing-than-r-d.
3. Swanson A. Big pharmaceutical companies are spending far more on marketing than research. *The Washington Post,* 2015.
4. Mulinari S, Ozieranski P. Disclosure of payments by pharmaceutical companies to healthcare professionals in the UK: analysis of the Association of the British Pharmaceutical Industry's Disclosure UK database, 2015 and 2016 cohorts. *BMJ Open* 2018;8(10):e023094. doi: 10.1136/bmjopen-2018-023094
5. Rodwin MA. Conflict of Interest in the Pharmaceutical Sector: A Guide for Public Management. *DePaul J Health Care L* 2019;21
6. Humphreys K, Shover CL, Andrews CM, et al. Responding to the opioid crisis in North America and beyond: recommendations of the Stanford-Lancet Commission. *The Lancet* 2022;399(10324):555-604. doi: 10.1016/S0140-6736(21)02252-2
7. Lo YL. Further insights on institutional conflicts of interest in research settings. *Proceedings of Singapore Healthcare* 2020;29(1):3-4. doi: 10.1177/2010105820909983
8. Schafer A. Institutional conflict of interest: attempting to crack the deferiprone mystery. *Journal of Medical Ethics* 2021;47(8):531. doi: 10.1136/medethics-2019-105498
9. Lo B, Field MJ. Conflict of interest in medical research, education, and practice. 2009
10. Lemmens T, Miller PB. The human subjects trade: ethical and legal issues surrounding recruitment incentives. *J Law Med Ethics* 2003;31(3):398-418. doi: 10.1111/j.1748-720x.2003.tb00103.x [published Online First: 2003/11/25]
11. Moynihan R, Bero L. Toward a Healthier Patient Voice: More Independence, Less Industry Funding. *JAMA Internal Medicine* 2017;177(3):350-51. doi: 10.1001/jamainternmed.2016.9179
12. Sismondo S. Ghost-managed medicine: Big pharma's invisible hands: Mattering Press 2018:8.
13. Rickard E, Ozieranski P. A hidden web of policy influence: The pharmaceutical industry's engagement with UK's All-Party Parliamentary Groups. *PLOS ONE* 2021;16(6):e0252551. doi: 10.1371/journal.pone.0252551
14. Marks JH. The perils of partnership: industry influence, institutional integrity, and public health: Oxford University Press 2019.
15. EFPIA. EFPIA CODE OF PRACTICE, 2019.
16. Mulinari S, Pashley D, Ozieranski P. Advancing international comparison of pharmaceutical industry funding of patient advocacy: Focus on Denmark. *Health Policy* 2022;126(12):1256-62. doi: https://doi.org/10.1016/j.healthpol.2022.11.003
17. Mulinari S, Martinon L, Jachiet P-A, et al. Pharmaceutical industry self-regulation and non-transparency: country and company level analysis of payments to healthcare professionals in seven European countries. *Health Policy* 2021;125(7):915-22. doi: https://doi.org/10.1016/j.healthpol.2021.04.015

For peer review only - http://bmjopen.bmj.com/site/about/guidelines.xhtml

18. Agrawal S, Brennan N, Budetti P. The Sunshine Act—effects on physicians. *N Engl J Med* 2013;2013(368):2054-57.
19. Ahmed R, Bae S, Hicks CW, et al. Here Comes the Sunshine: Industry's Payments to Cardiothoracic Surgeons. *Annals of Thoracic Surgery* 2017;103(2):567-72. doi: 10.1016/j.athoracsur.2016.06.053
20. Braithwaite J, Frane N, Partan MJ, et al. Review of Industry Payments to General Orthopaedic Surgeons Reported by the Open Payments Database: 2014 to 2019. *JAAOS Global Research & Reviews* 2021;5(5)
21. Fleischman W, Ross JS, Melnick ER, et al. Financial Ties Between Emergency Physicians and Industry: Insights From Open Payments Data. *Annals of Emergency Medicine* 2016;68(2):153-58.e4. doi: 10.1016/j.annemergmed.2016.01.014
22. Maruf M, Sidana A, Fleischman W, et al. Financial Relationships between Urologists and Industry: An Analysis of Open Payments Data. *Urology Practice* 2018;5(3):180-86. doi: 10.1016/j.urpr.2017.03.012
23. Slentz DH, Nelson CC, Lichter PR. Characteristics of Industry Payments to Ophthalmologists in the Open Payments Database. *JAMA Ophthalmology* 2019;137(9):1038-44. doi: 10.1001/jamaophthalmol.2019.2456
24. Moynihan R, Fabbri A, Parker L, et al. Mixed methods evaluation of workshops for citizen health advocates about financial conflicts of interests in healthcare. *BMJ Open* 2020;10(5) doi: 10.1136/bmjopen-2019-034195
25. Ozieranski P, Csanadi M, Rickard E, et al. Analysis of Pharmaceutical Industry Payments to UK Health Care Organizations in 2015. *JAMA Network Open* 2019;2(6):e196253-e53. doi: 10.1001/jamanetworkopen.2019.6253
26. Parker L, Karanges EA, Bero L. Changes in the type and amount of spending disclosed by Australian pharmaceutical companies: an observational study. *BMJ open* 2019;9(2):e024928.
27. Fabbri A, Grundy Q, Mintzes B, et al. A cross-sectional analysis of pharmaceutical industry-funded events for health professionals in Australia. *BMJ open* 2017;7(6):e016701.
28. Moriarty F, Larkin J, Fahey T. Payments reported by the pharmaceutical industry in Ireland from 2015 to 2019: an observational study. *Health Policy* 2021 doi: https://doi.org/10.1016/j.healthpol.2021.07.016
29. Ozaki A, Saito H, Senoo Y, et al. Overview and transparency of non-research payments to healthcare organizations and healthcare professionals from pharmaceutical companies in Japan: Analysis of payment data in 2016. *Health Policy* 2020;124(7):727-35. doi: https://doi.org/10.1016/j.healthpol.2020.03.011
30. Ozaki A, Saito H, Onoue Y, et al. Pharmaceutical payments to certified oncology specialists in Japan in 2016: a retrospective observational cross-sectional analysis. *BMJ Open* 2019;9(9):e028805. doi: 10.1136/bmjopen-2018-028805
31. Anderson TS, Gellad WF, Good CB. Characteristics of biomedical industry payments to teaching hospitals. *Health Affairs* 2020;39(9):1583-91. doi: 10.1377/hlthaff.2020.00385
32. Association of the British Pharmaceutical Industry. Working with patients and patient organisations. A sourcebook for industry, 2022.
33. McCoy MS, Carniol M, Chockley K, et al. Conflicts of interest for patient-advocacy organizations. *New England Journal of Medicine* 2017;376(9):880-85.

For peer review only

For peer review only - http://bmjopen.bmj.com/site/about/guidelines.xhtml

34. Rose SL, Highland J, Karafa MT, et al. Patient Advocacy Organizations, Industry Funding, and Conflicts of Interest. *JAMA Internal Medicine* 2017;177(3):344-50. doi: 10.1001/jamainternmed.2016.8443
35. Ozieranski P, Martinon, L., Jachiet, P., Mulinari, S. Accessibility and quality of drug company disclosures of payments to healthcare professionals and organisations in 37 countries: A European policy review. *BMJ Open* 2021
36. Fabbri A, Parker L, Colombo C, et al. Industry funding of patient and health consumer organisations: systematic review with meta-analysis. *BMJ* 2020;368:l6925. doi: 10.1136/bmj.l6925
37. EFPIA. EFPIA CODE OF PRACTICE ON RELATIONSHIPS BETWEEN THE PHARMACEUTICAL INDUSTRY AND PATIENT ORGANISATIONS, 2011.
38. Ozieranski P, Rickard E, Mulinari S. Exposing drug industry funding of UK patient organisations. *BMJ* 2019;365:l1806. doi: 10.1136/bmj.l1806
39. Ozieranski P, Csanadi M, Rickard E, et al. Underreporting of drug industry payments to patient organisations in the UK (2012-2016). 2020
40. Mulinari S, Vilhelmsson A, Rickard E, et al. Five years of pharmaceutical industry funding of patient organisations in Sweden: Cross-sectional study of companies, patient organisations and drugs. *PLOS ONE* 2020;15(6):e0235021. doi: 10.1371/journal.pone.0235021
41. Pashley D OP, Mulinari S. . Disclosure of pharmaceutical industry funding of patient organizations in Nordic countries: can industry self-regulation deliver on its transparency promise? *International Journal of Health Services* 2022
42. Rickard E, Ozieranski P, Mulinari S. Evaluating the transparency of pharmaceutical company disclosure of payments to patient organisations in the UK. *Health Policy* 2019;123(12):1244-50.
43. EFPIA. The EFPIA Disclosure Code: Your Questions Answered, 2014.
44. Association of the British Pharmaceutical Industry. Definitions - Disclosure 2022 [Available from: https://www.abpi.org.uk/reputation/disclosure-uk/about-disclosure-uk/definitions-disclosure/.
45. Keller AC, Packel L. Going for the cure: patient interest groups and health advocacy in the United States. *J Health Polit Policy Law* 2014;39(2):331-67. doi: 10.1215/03616878-2416238 [published Online First: 2013/12/07]
46. Hegde D, Sampat B. Can private money buy public science? Disease group lobbying and federal funding for biomedical research. *Management Science* 2015;61(10):2281-98.
47. Moberly T. CCGs fail to declare pharma funding. *BMJ* 2018;360:j5911. doi: 10.1136/bmj.j5911
48. Niforatos JD, Boas S, Hertz S, et al. Analysis of current financial relationships between emergency physicians and industry. *American Journal of Emergency Medicine* 2019;37(4):722-25. doi: 10.1016/j.ajem.2018.12.039
49. Cheng T, Boelitz K, Rybin D, et al. Nationwide patterns in industry payments to academic vascular surgeons. *Journal of Vascular Surgery* 2021;73(2):675-81. doi: 10.1016/j.jvs.2020.04.527
50. Garstka ME, Monlezun D, DuCoin C, et al. The Sunshine Act and surgeons: a nation-wide analysis of industry payments to physicians. *Journal of Surgical Research* 2019;233:41-49. doi: 10.1016/j.jss.2018.07.004

For peer review only

51. Cuomo RE, Cai M, Shah N, et al. Physicians payment in the United States between 2014 and 2018: An analysis of the CMS Open Payments database. *PLOS ONE* 2021;16(6):e0252656. doi: 10.1371/journal.pone.0252656
52. Mejia J, Mejia A, Pestilli F. Open data on industry payments to healthcare providers reveal potential hidden costs to the public. *Nature Communications* 2019;10(1) doi: 10.1038/s41467-019-12317-z
53. Ozieranski P, Pitter J, Rickard E, et al. A “patient-industry complex”? Investigating the financial dependency of UK patient organisations on drug company funding. *Sociology of Health & Illness* 2021
54. EFPIA. The Pharmaceutical Industry in Figures, 2016.
55. Hyndman N. Editorial: The charity sector— changing times, changing challenges. *Public Money & Management* 2017;37(3):149-53. doi: 10.1080/09540962.2017.1281608
56. Greer SL. Territorial politics and health policy: UK health policy in comparative perspective: Manchester University Press 2004.
57. Birrell D. Comparing devolved governance: Palgrave Macmillan 2012.
58. Cheung A, Paun A, Valsamidis L. Devolution at 20. Institute for Government, 2019.
59. ONS. Population estimates for the UK, England and Wales, Scotland and Northern Ireland: mid-2015 2016 [Available from: https://www.ons.gov.uk/peoplepopulationandcommunity/populationandmigration/populationestimates/bulletins/annualmidyearpopulationestimates/mid2015 accessed 13th September 2021.
60. Office for National Statistics. Life expectancy for local areas of the UK: between 2001 to 2003 and 2017 to 2019 2020 [Available from: https://www.ons.gov.uk/peoplepopulationandcommunity/healthandsocialcare/healthandlifeexpectancies/bulletins/lifeexpectancyforlocalareasoftheuk/between2001to2003and2017to2019 accessed 28th August 2021.
61. Saghy E, Mulinari S, Ozieranski P. Drug company payments to General Practices in England: cross-sectional and social network analysis. *PLOS ONE* 2021
62. Kirchhof P. A tale of two countries: how decentralized organization and long-term investment build resilient healthcare systems. *Eur Heart J Qual Care Clin Outcomes* 2020;6(3):201-03. doi: 10.1093/ehjqcco/qcaa036 [published Online First: 2020/05/07]
63. Martin-Moreno JM, Alonso P, Claveria A, et al. Spain: a decentralised health system in constant flux. *BMJ* 2009;338:b1170. doi: 10.1136/bmj.b1170
64. Rahul A. ea. Leveraging CMS Open Payments Data to Identify Channel Preferences and Gather Competitive Intelligence, Thereby Improving HCP Targeting. *Pharmaceutical Management Science Association* 2021
65. Hollands S. Receipt of Promotional Payments at the Individual and Physician Network Level Associated with Higher Branded Antipsychotic Prescribing Rates. *Adm Policy Ment Health* 2020;47(1):73-85. doi: 10.1007/s10488-019-00974-7
66. Agha L, Zeltzer D. DRUG DIFFUSION THROUGH PEER NETWORKS: THE INFLUENCE OF INDUSTRY PAYMENTS. *NBER WORKING PAPER SERIES* 2020;Working Paper 26338
67. Anand R CD, Yan J, et al. Leveraging CMS Open Payments Data to Identify Channel Preferences and Gather Competitive Intelligence, Thereby Improving HCP Targeting. *Journal of the Pharmaceutical Management Science Association* 2017 doi: https://www.pmsa.org/jpmsa-vol05-article06

For peer review only - http://bmjopen.bmj.com/site/about/guidelines.xhtml

68. Mulinari S, Ozieranski P. Capitalizing on transparency: Commercial surveillance and pharmaceutical marketing after the Physician Sunshine Act. *Big Data & Society* 2022;9(1):20539517211069631. doi: 10.1177/20539517211069631
69. Oldani MJ. Tales From The" Script": An Insider/Outside View Of Pharmaceutical Sales Practice. *Kroeber Anthropological Society Papers* 2002:147-76.
70. Association of the British Pharmaceutical Industry. CODE OF PRACTICE for the PHARMACEUTICAL INDUSTRY 2015, 2015.
71. Dunn OJ. Multiple Comparisons Using Rank Sums. *Technometrics* 1964;6(3):241-52. doi: 10.1080/00401706.1964.10490181
72. Baadh AS, Baadh PK, Islam S, et al. IR and the Sunshine Act: Two-Year Analysis of the Open Payments Database and Comparison with Related Specialties. *Journal of Vascular and Interventional Radiology* 2017;28(2):200-05. doi: 10.1016/j.jvir.2016.10.008
73. Morse E, Fujiwara RJT, Mehra S. Increasing Industry Involvement in Otolaryngology: Insights from 3 Years of the Open Payments Database. *Otolaryngology - Head and Neck Surgery (United States)* 2018;159(3):501-07. doi: 10.1177/0194599818778502
74. Baadh AS, Patel M, Patel K, et al. Medical industry at tumor board: Three-years analysis of the open payments database and comparison of oncologic specialties. *Journal of Surgical Oncology* 2018;117(4):551-57. doi: 10.1002/jso.24899
75. Borgatti SP, Everett MG, Johnson JC. Analyzing social networks: Sage 2018.
76. Snijders TA, Faye M, Brailly J. Network dynamics with a nested node set: Sociability in seven villages in Senegal. *Statistica Neerlandica* 2020;74(3):300-23.
77. Borgatti SP, Everett, M.G. and Freeman, L.C. . Ucinet for Windows: Software for Social Network Analysis. Harvard, MA: Analytic Technologies 2002.
78. Borgatti S. Centrality and Centralization 2000 [Available from: http://www.analytictech.com/mb119/chapter5.htm accessed 20th July 2021.
[dataset] 79. Rickard E, Ozieranski, P.,. Dataset for "Comparing pharmaceutical company payments in the four UK countries: a cross-sectional and social network analysis": Bath: University of Bath Research Data Archive, 2023.
80. Welsh Government. NHS Wales health boards and trusts 2022 [Available from: https://www.gov.wales/nhs-wales-health-boards-and-trusts.
81. NHS Scotland. NHSScotland Health Boards 2022 [Available from: https://www.careers.nhs.scot/careers/find-your-career/international-recruitment/nhsscotland-health-boards/.
82. Foundation Trust Network. BUILDING A HEALTHY NHS AROUND PEOPLE'S NEEDS. AN INTRODUCTION TO NHS FOUNDATION TRUSTS AND TRUSTS, 2015.
83. Noclor. EVERY NHS TRUST IN ENGLAND IS NOW RESEARCH-ACTIVE 2022 [Available from: https://www.noclor.nhs.uk/every-nhs-trust-england-now-research-active.
84. NHS Confederation. What are clinical commissioning groups? 2021 [Available from: https://www.nhsconfed.org/articles/what-are-clinical-commissioning-groups.
85. Association of the British Pharmaceutical Industry. Joint Working: A toolkit for industry and the NHS, 2019.
86. Brauer PR, Morse E, Mehra S. Industry Payments for Otolaryngology Research: A Four-Year Analysis of the Open Payments Database. *Laryngoscope* 2020;130(2):314-20. doi: 10.1002/lary.27896

For peer review only - http://bmjopen.bmj.com/site/about/guidelines.xhtml

87. Fabbri A, Swandari S, Lau E, et al. Pharmaceutical industry funding of health consumer groups in Australia: a cross-sectional analysis. *International Journal of Health Services* 2019;49(2):273-93.
88. Baggott R, Allsop J, Jones K. Speaking for patients and carers: health consumer groups and the policy process: Macmillan International Higher Education 2014.
89. Keefe PR. Empire of pain: The secret history of the Sackler dynasty: Anchor 2021.
90. Marks JH. Lessons from Corporate Influence in the Opioid Epidemic: Toward a Norm of Separation. *Journal of Bioethical Inquiry* 2020;17(2):173-89. doi: 10.1007/s11673-020-09982-x
91. Moberly T. NHS joint working with industry is out of public sight. *BMJ* 2019;364:l1353. doi: 10.1136/bmj.l1353
92. Ozaki POHSERSMA. International comparison of pharmaceutical industry payment disclosures in the UK and Japan: implications for self-regulation, public regulation, and transparency. *Globalization and Health* 2023 doi: 10.1186/s12992-022-00902-9
93. Kanter GP, Loewenstein G. Evaluating open payments. *Jama* 2019;322(5):401-02.
94. Association of the British Pharmaceutical Industry. Disclosure UK 2022 [Available from: https://www.abpi.org.uk/reputation/disclosure-uk/.

For peer review only

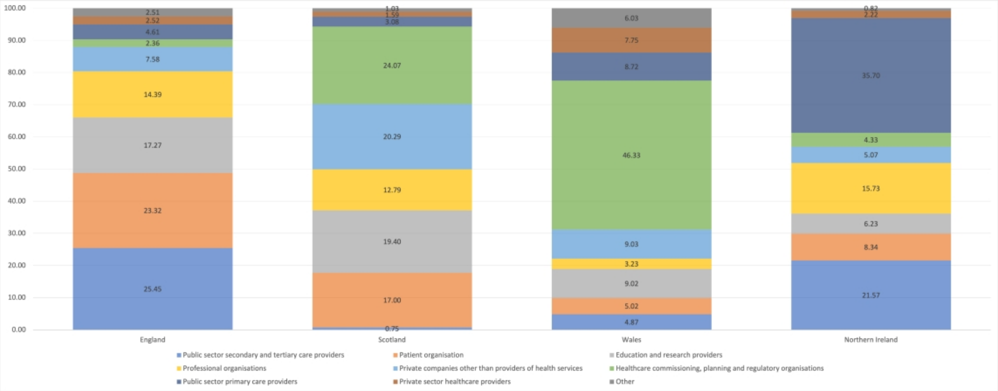

Percentage of payments to recipient types per country

541x213mm (330 x 330 DPI)

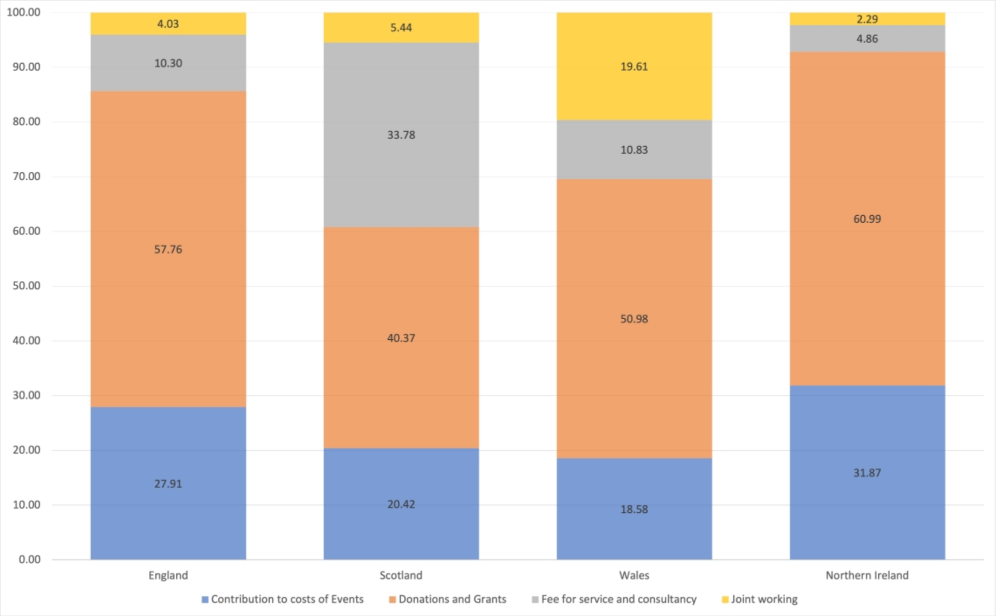

Percentage of total value for each payment type

346x214mm (330 x 330 DPI)

Supplementary file contents:

Supplementary File	Page
Supplementary File 1. Payment types included in Disclosure UK and patient organisation codes applied to Disclosure UK codes	2
Supplementary File 2. Data integration flowchart	3
Supplementary File 3. Recipient category descriptions and examples	4-5
Supplementary File 4. Top 10% of donors in each country	6
Supplementary File 5. Visualised networks for England (a), Scotland (b), Wales (c), Northern Ireland (d)	7-10
Supplementary File 6. Top ten companies by degree centrality scores in each country	11
Supplementary File 7. Descriptive statistics for each recipient category	12-14
Supplementary File 8. Top 10 recipients in each country	15-16
Supplementary File 9. Post-hoc Bonferroni pairwise comparisons between countries of payments per category	17-19
Supplementary File 10. Post-hoc Bonferroni pairwise comparisons between countries of payment types	20

For peer review only

Supplementary File 1. Payment types included in Disclosure UK and patient organisation codes applied to Disclosure UK codes

**Payment type**	**Description of payment type**	**Patient organisation payments subsumed within the payment type**
Contribution to costs of Events	Contribution to costs related to Events, through HCOs or Third Parties, including support to HCPs to attend Events, such as: • Registration fees; • Sponsorship agreements with HCOs or with Third Parties appointed by an HCO to manage an Event; and • Travel and accommodation (EFPIA Code of Practice 2019, p. 30)	contributions to costs of events organised by recipients or third parties; travel, accommodation and registration fees
Donations and Grants to HCOs	Donations and Grants to HCOs that support healthcare, including donations and grants (either cash or benefits in kind) to institutions, organisations or associations that are comprised of HCPs and/or that provide healthcare (EFPIA Code of Practice 2019, p. 30)	donations; grants; corporate member, supporter, sponsor or partner; purchases and subscriptions from patient organisations; more than one distinct payment form; form of funding unclear; sponsorships
Fee for service and consultancy	Payments resulting from or related to contracts between Member Companies and HCOs under which such HCOs provide any type of services to a Member Company or any other type of funding not covered in the previous categories. Fees, on the one hand, and on the other hand payments relating to expenses agreed in the written agreement covering the activity will be disclosed as two separate amounts. (EFPIA Code of Practice 2019, p. 30)	fees for service and consultancy (including travel and accommodation); support, help and contributions
Joint working	The Department of Health defines joint working between the NHS and the pharmaceutical industry as situations where, for the benefit of patients, one or more pharmaceutical companies and the NHS pool skills, experience and/or resources for the joint development and implementation of patient centred projects and share a commitment to successful delivery. (ABPI Code of Practice 2015, Clause 20, p. 30)	n/a

For peer review only

Supplementary File 2. Data integration flowchart

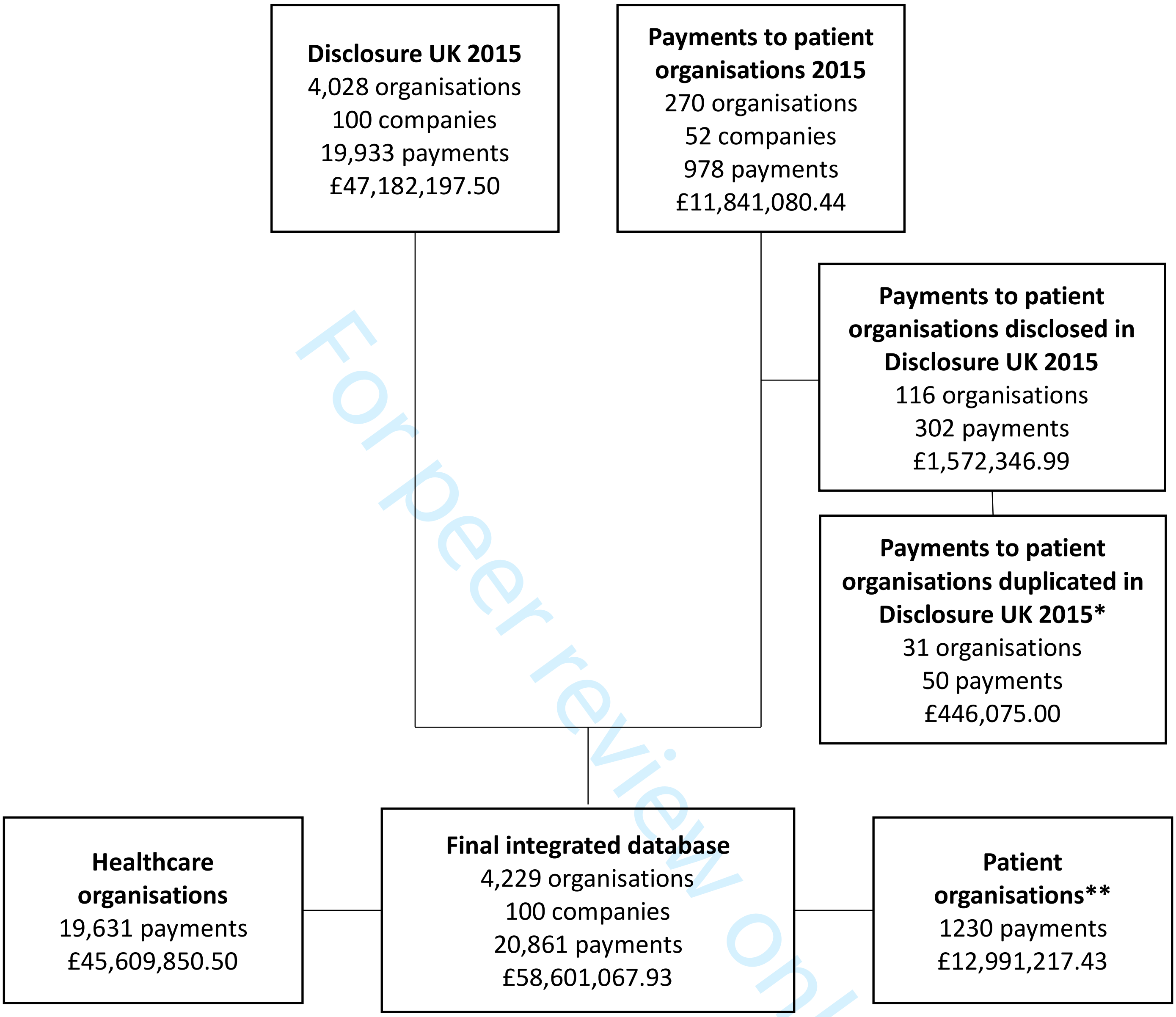

For peer review only

*This is the number and value of payments excluded to ensure no payment was counted twice

**During the cleaning process, a number of considerations took place to determine the final number and value of payments to patient organisations. Some duplicate payments were identified that were reported as multiple payments in one dataset and one payment in the other dataset (influencing the final number of payments). Approaches to VAT when reporting values also differed between the two datasets (influencing the final value of payments).

Supplementary File 3. Recipient category descriptions and examples

**Recipient category**	**Category description**	**Examples (country-specific examples where applicable)**
Alternative providers of health services	Charities, not-for-profit companies, social enterprises and community interest companies providing health services	- social enterprise delivering primary or secondary care health services<br>- nursing or care home run by a community interest company (CIC)<br>- hospital, hospice, or nursing home with a charitable status
Education and research providers	Universities, charities, and noncommercial institutes undertaking research	- university<br>- research institute at a NHS organisation<br>- charity focusing on undertaking medical research
Formal bodies representing healthcare professionals or patients	Local medical, optical, optometric, or pharmaceutical committees and statutory bodies representing healthcare professionals or patients	- local medical committees (LMC)<br>- local optical or optometric committee (LOC)<br>*England*<br>- local pharmaceutical committee (LPC)
Charities and other third-sector organisations (excluding providers of health services, professional organisations, and patient organisations)	Organisations (not patient organisations) focusing on education, research, advocacy, and multipurpose organisations	- charitable trusts providing medical education events to healthcare professionals<br>- think tanks<br>- third-sector organisation (non-charity) or charity focused on funding medical research<br>- research institute registered as a charitable organisation
Healthcare commissioning, planning and regulatory organisations	Local, regional, and commissioning, planning, or regulatory organisations	- primary care trust (PCT)<br>- NHS Shared Business Services<br>- National Institute for Health and Care Excellence (NICE)<br>- Public Health England<br>*England*<br>- clinical commissioning group<br>- Locality group<br>- area prescribing committee<br>- local commissioning group<br>- NHS England<br>*Scotland*<br>- regional NHS board<br>- area pharmaceutical committee<br>*Wales*<br>- health board<br>- public health Wales

For peer review only - http://bmjopen.bmj.com/site/about/guidelines.xhtml

		Northern Ireland<br>- health and social care board<br>- local commissioning group
Patient organisations	Organisations focusing on supporting education, research, advocacy, and multipurpose organisations	- multipurpose patient organisations<br>- organisations focused on providing patient support<br>- hospital charities
Private companies other than providers of health services	Providers of medical communications or training services, commercial or medical research services, and accountancy or consultancy services	- manufacturer or supplier of medical devices or technologies<br>- pharmacy wholesaler or distributor<br>- event management services<br>- journal or publishing company<br>- clinical or contract research organisation<br>- private laboratory
Private sector healthcare providers	Private clinics and hospitals, healthcare groups, and providers of dental, pharmacy, and optical services	- dental practice<br>- pharmacy or chemist<br>- opticians<br>- private clinic, surgery, practice, or hospital<br>- private company providing community health or social care services
Professional organisations	Organisations of medical professionals, other healthcare professionals, or non-healthcare professionals,	- organisation of medical professionals<br>- professional bodies responsible for setting standards of care and education for medical specialities<br>- royal college - medical professionals<br>- alliance or coalition of professional associations or groups<br>- professional organisation of pharmacists or pharmacy technicians
Public administration and providers of public services	Central UK government bodies, devolved administrations in Scotland, Wales, and Northern Ireland, and local authorities	- district, city, country, or borough council<br>- prison<br>- devolved administrations<br>- central government bodies
Public sector primary care providers	General practitioner surgeries, medical practice centres, groups of surgeries or medical practices, and healthcare or medical groups	- GP practice, surgery, medical practice or family practice<br>- health centre, medical centre or primary care centre<br>- group of surgeries or medical practices
Public sector secondary and tertiary care providers	NHS trusts, NHS hospitals, and networks and collaboratives of NHS organisations	- NHS hospital<br>- NHS Foundation Trust<br>- NHS rust<br>- strategic clinical network (SCN)<br>*Scotland*<br>- managed clinical network (MCN)

For peer review only

Supplementary File 4. Top 10% of donors in each country

Country	Company (revenue ranking)	Payment value - £ (%)	Payments – n (%)
England	Pfizer (2)	5,292,130.74 (10.09)	1636 (8.99)
	Novartis (7)	3,564,500.43 (6.80)	460 (2.53)
	Bayer (8)	3,476,304.44 (6.63)	2110 (11.60)
	GlaxoSmithKline (4)	3,291,496.35 (6.28)	1076 (5.92)
	AstraZeneca (1)	2,779,000.54 (5.30)	1279 (7.03)
	Janssen-Cilag (10)	2,387,242.64 (4.55)	722 (3.97)
	UCB Pharma (30)	2,204,967.90 (4.20)	74 (0.41)
	Astellas Pharma (21)	2,044,050.60 (3.90)	311 (1.71)
	Roche (5)	1,931,651.77 (3.68)	173 (0.95)
	Biogen Idec (23)	1,886,879.26 (3.60)	83 (0.46)
	Top 10% total	28,858,224.67 (55.03)	7924 (43.56)
Scotland	Biogen Idec (24)	733,104.05 (20.09)	7 (0.51)
	Takeda UK (38)	274,952.71 (7.53)	25 (1.82)
	Pfizer (2)	250,859.45 (6.87)	143 (10.44)
	Bayer (8)	215,930.76 (5.92)	164 (11.97)
	Novartis (7)	199,703.97 (5.47)	48 (3.50)
	Bristol-Myers Squibb (23)	183,959.00 (5.04)	51 (3.72)
	AstraZeneca (1)	178,848.49 (4.90)	75 (5.47)
	Top 10% total	2,037,358.42 (55.82)	513 (37.45)
Wales	Pfizer (2)	284,719.57 (14.32)	102 (10.30)
	Roche (5)	230,090.90 (11.58)	10 (1.01)
	Novartis (7)	177,069.59 (8.91)	36 (3.64)
	AstraZeneca (1)	148,288.67 (7.46)	79 (7.98)
	Janssen-Cilag (10)	122,237.44 (6.15)	22 (2.22)
	Biogen (24)	112,428.62 (5.66)	7 (0.71)
	Top 10% total	1,074,834.80 (54.07)	256 (25.86)
Northern Ireland	Sanofi Aventis (13)	92,252.80 (17.81)	24 (7.72)
	Pfizer (2)	86,639.31 (16.73)	45 (14.47)
	Napp Pharmaceuticals (29)	83,252.29 (16.07)	45 (14.47)
	Bayer (8)	37,959.50 (7.33)	75 (24.12)
	Top 10% total	300,103.90 (57.94)	189 (60.77)

*Value as proportion of all payments in each country

For peer review only - http://bmjopen.bmj.com/site/about/guidelines.xhtml

Supplementary File 5. Visualised networks for England (a), Scotland (b), Wales (c), Northern Ireland (d)

Supplementary File 5a. England's network

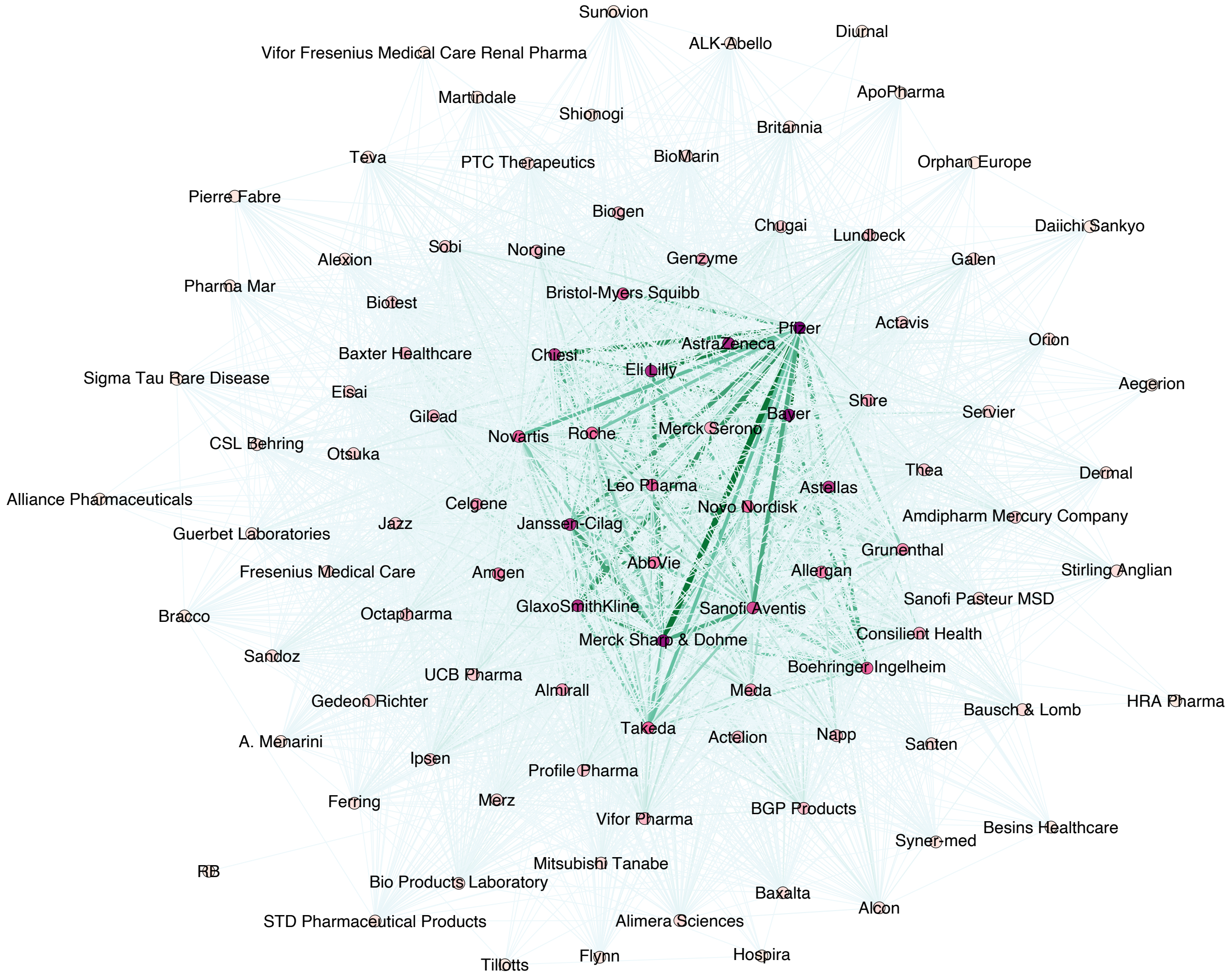

only

Supplementary File 5b. Scotland's network

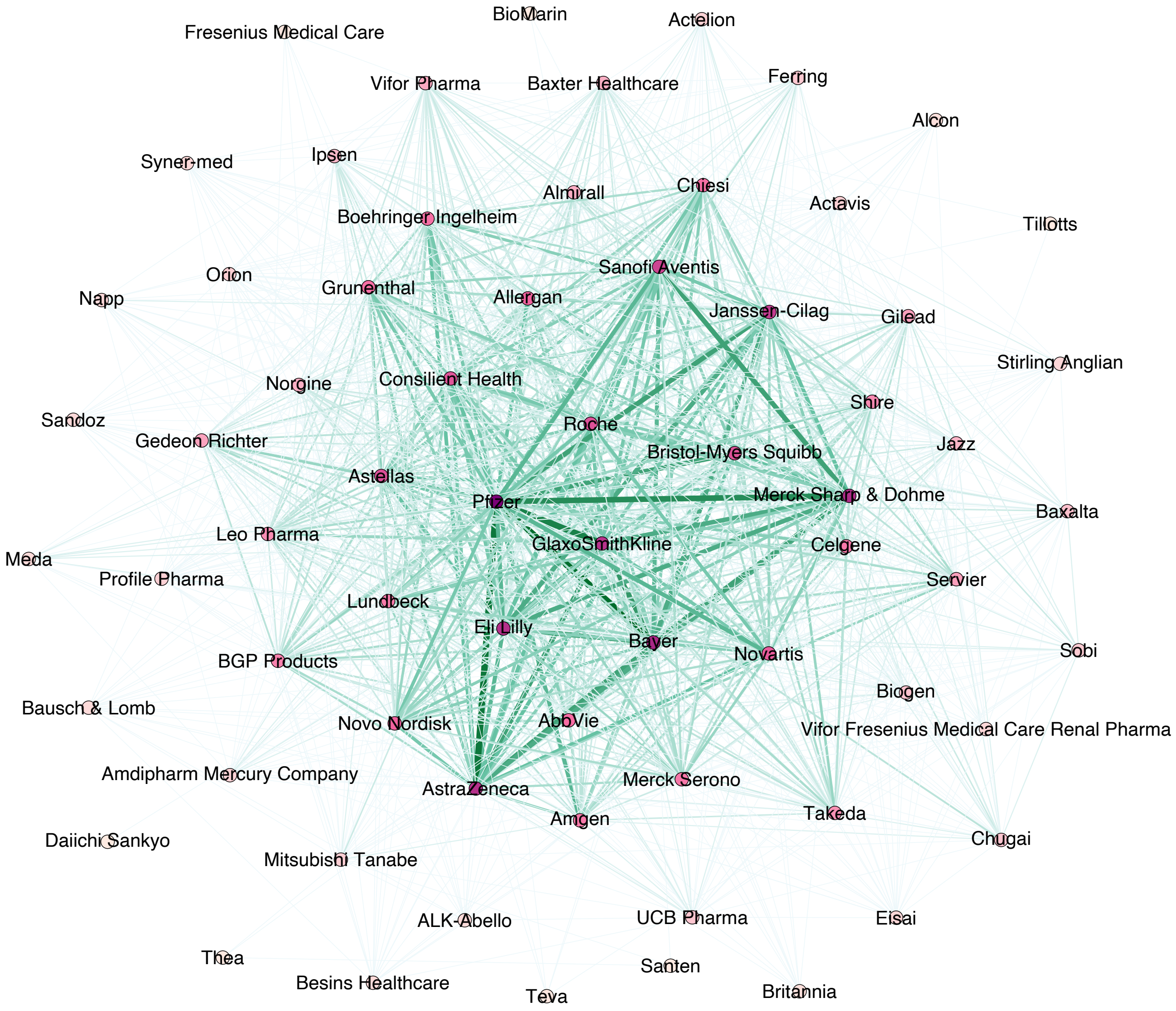

Supplementary File 5c. Wales' network

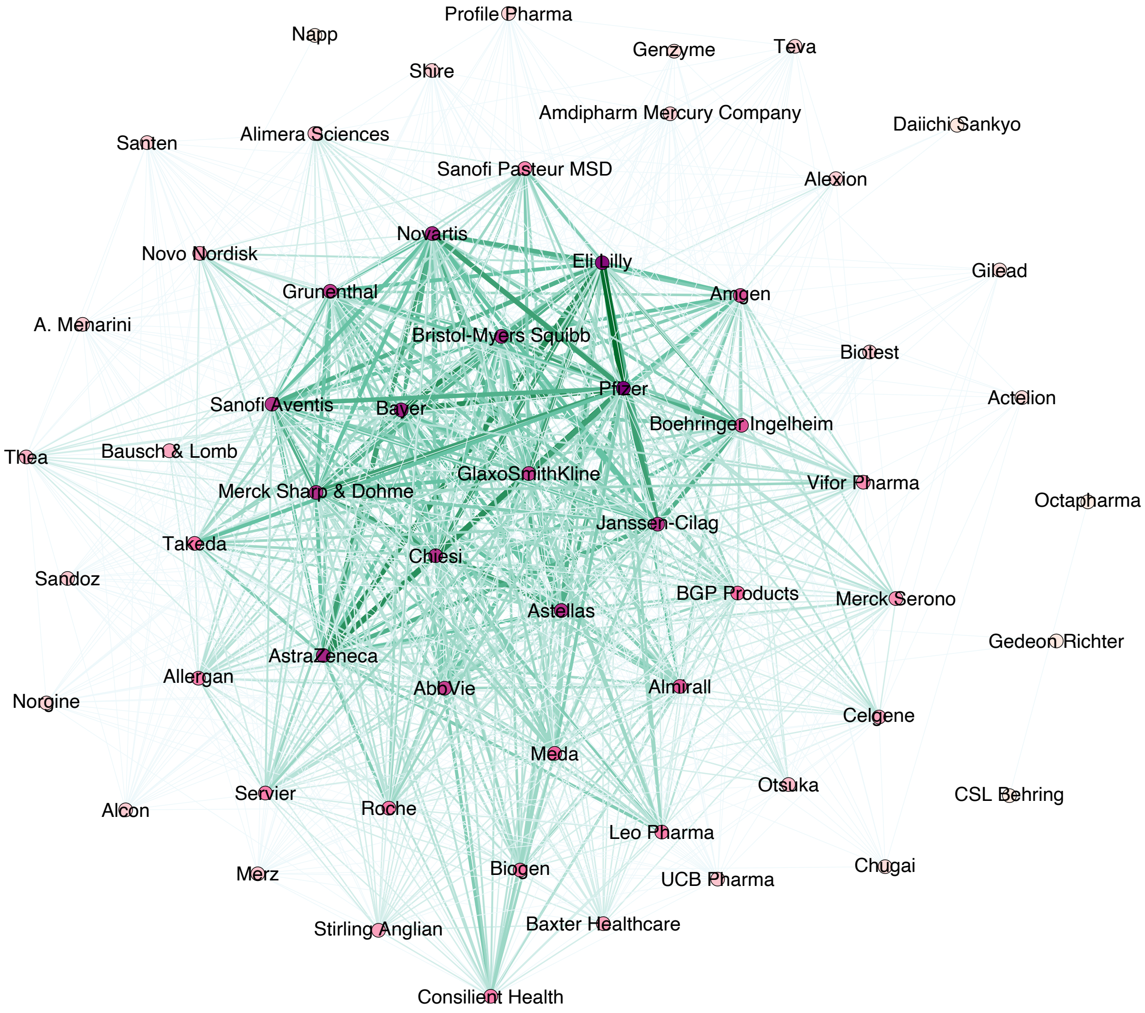

9

Supplementary File 5d. Northern Ireland's network

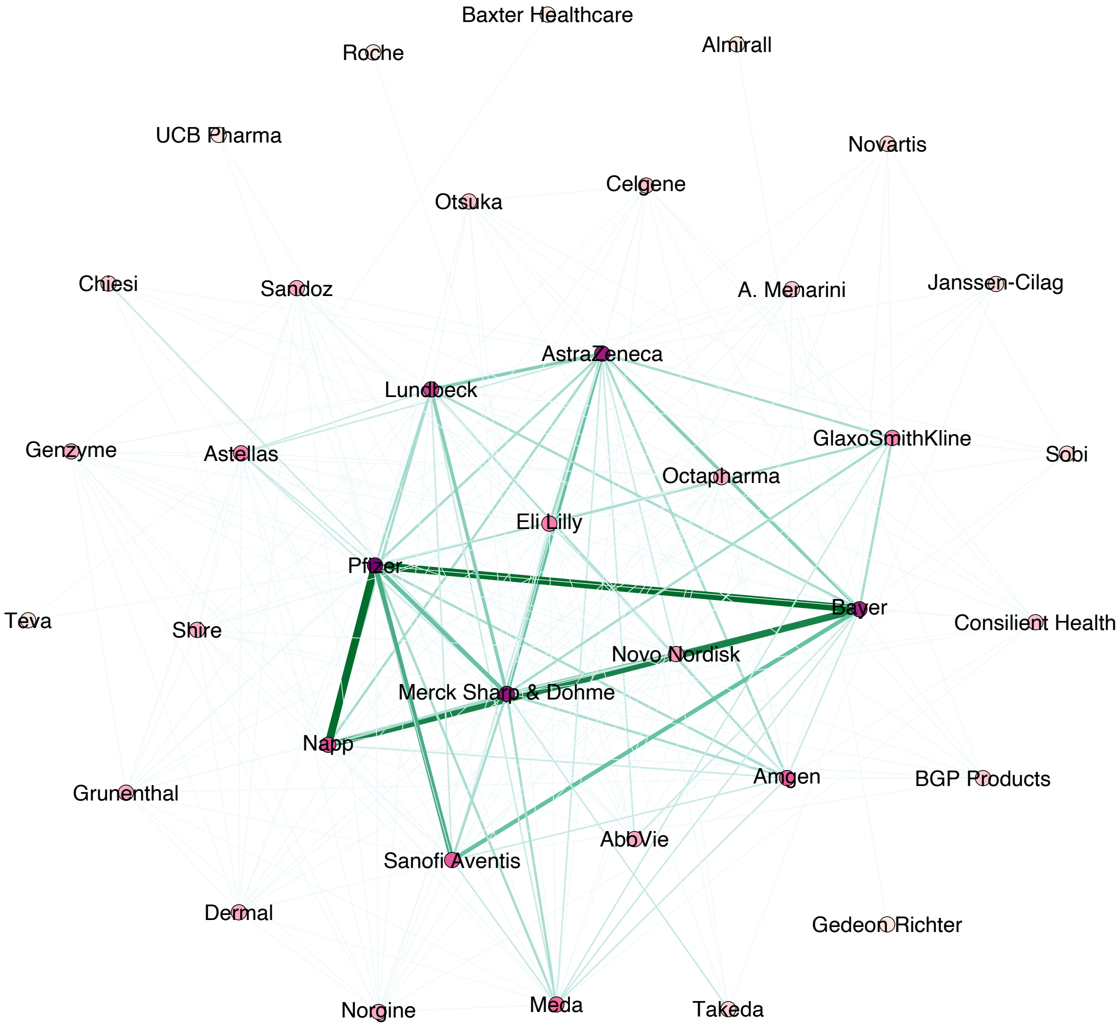

Note. All networks were visualised in Gephi v 0.9.2. Node label size and darkness corresponds to the weighted degree centrality of each company; the size and darkness of the edges (connecting lines) correspond to the number of shared recipients between companies

For peer review only - http://bmjopen.bmj.com/site/about/guidelines.xhtml

Supplementary File 6. Top ten companies by degree centrality scores in each country

England		Scotland		Wales		Northern Ireland	
Company	Degree	Company	Degree	Company	Degree	Company	Degree
Pfizer (2)	3394	Pfizer (2)	319	Pfizer (2)	206	Pfizer (2)	63
Merck Sharp & Dohme (6)	3064	Bayer (8)	260	Eli Lilly (3)	196	Merck Sharp & Dohme (6)	57
Bayer (8)	3060	Merck Sharp & Dohme (6)	251	Bayer (8)	176	AstraZeneca (1)	55
AstraZeneca (1)	2755	AstraZeneca (1)	245	AstraZeneca (1)	171	Bayer (8)	52
Eli Lilly (3)	2741	Eli Lilly (3)	245	Bristol-Myers Squibb (23)	169	Lundbeck (64)	43
Janssen-Cilag (10)	2539	GlaxoSmithKline (4)	241	Novartis (7)	168	Napp Pharmaceuticals (29)	39
GlaxoSmithKline (4)	2531	Janssen-Cilag (10)	227	Merck Sharp & Dohme (6)	161	Amgen (19)	36
Astellas Pharma (21)	2410	Sanofi Aventis (13)	214	Astellas Pharma (21)	160	Sanofi Aventis (13)	36
Chiesi (27)	2365	Astellas Pharma (21)	196	Chiesi (27)	158	Meda Pharma (46)	32
Sanofi Aventis (13)	2156	Bristol-Myers Squibb (23)	193	Sanofi Aventis (13)	156	Eli Lilly (3)	27

Note. Numbers in brackets represent company size (revenue ranking)

For peer review only

Supplementary File 7. Descriptive statistics for each recipient type

England – types of recipient	Value (%)	Payments – n (%)	Median - £ (IQR)	Recipients – n (%)	Pharmaceutical companies
Public sector secondary and tertiary care providers	**13,349,779.1 (25.56)**	**6660 (36.87)**	233.17 (141.87 – 500)	260 (7.41)	**89**
Patient organisation	12,227,843.2 (23.41)	1141 (6.32)	**4000 (500 – 11,104)**	288 (8.21)	65
Education and research providers	9,055,882.96 (17.34)	875 (4.84)	1000 (333.34 – 4,798.40)	56 (1.60)	68
Professional organisations	7,545,121.68 (14.44)	1776 (9.83)	500 (240 – 3,200)	354 (10.09)	84
Private companies other than providers of health services	3,975,461.63 (7.61)	1443 (7.99)	300 (196.8 – 598.92)	239 (6.81)	56
Public sector primary care providers	2,416,957.98 (4.63)	2513 (13.91)	434.5 (193.6 – 869)	**1809 (51.55)**	32
Private sector healthcare providers	1,322,785.04 (2.53)	463 (2.56)	240 (166 – 588)	108 (3.08)	44
Healthcare commissioning, planning and regulatory organisations	1,235,239.68 (2.36)	2166 (11.99)	208.17 (160 – 307.2)	206 (5.87)	47
Charities and other third-sector organisations	876,822.76 (1.68)	366 (2.03)	223.52 (157 – 487.2)	39 (1.11)	40
Formal bodies representing healthcare professionals or patients	121,351.93 (0.23)	458 (2.54)	200 (160 – 259.8)	68 (1.94)	25
Alternative providers of health services	93,534.91 (0.18)	180 (1.00)	200 (160 – 394)	62 (1.77)	28
Public administration and providers of public services	15,335.25 (0.03)	24 (0.13)	394.4 (224.45 – 546.67)	20 (0.57)	10
All payments	*52,445,615.48*	*18065*	*280 (160 – 827.75)*	*3509*	*100*
**Scotland - types of recipient**	**Value (%)**	**Payments - n (%)**	**Median - £ (IQR)**	**Recipients - n (%)**	**Pharmaceutical companies (%)**
Healthcare commissioning, planning and regulatory organisations	**878,333.57 (24.13)**	**582 (43.30)**	240 (131.18 - 500)	22 (8.30)	**53 (73.61)**
Private companies other than providers of health services	740,694.09 (20.35)	25 (1.86)	1,200 (350 - 5,528.88)	11 (4.15)	13 (18.06)
Education and research providers	708,149.16 (19.46)	141 (10.49)	1152 (400 – 2,880)	8 (3.02)	41 (56.94)
Patient organisation	620,384.33 (17.05)	52 (3.87)	1000 (253.68 - 9,745)	14 (5.28)	19 (26.39)
Professional organisations	466,833.11 (12.83)	291 (21.65)	450 (285.67 - 980)	64 (24.15)	52 (72.22)

For peer review only - http://bmjopen.bmj.com/site/about/guidelines.xhtml

Public sector primary care providers	112,308.91 (3.09)	128 (9.52)	434.5 (202.23 - 651,75)	**113 (42.64)**	13 (18.06)
Private sector healthcare providers	58,091.76 (1.60)	69 (5.13)	647.54 (206.65-1,500)	11 (4.15)	9 (12.50)
Public sector secondary and tertiary care providers	27,392.82 (0.75)	39 (2.90)	300 (189.75 - 612)	12 (4.53)	11 (15.28)
Charities and other third-sector organisations	19,710 (0.54)	4 (0.30)	**1700 (377.5 - 6,250)**	2 (0.75)	4 (5.56)
Alternative providers of health services	4,580 (0.13)	6 (0.45)	700 (360 – 1,100)	4 (1.51)	4 (5.56)
Public administration and providers of public services	2,700 (0.07)	5 (0.37)	540 (200 - 600)	3 (1.13)	5 (6.94)
Formal bodies representing healthcare professionals or patients	427.2 (0.01)	2 (0.15)	213.6 (211.4 - 214.8)	1 (0.38)	1 (1.39)
All payments	*3,649,749.43*	*1,344*	*400 (180 - 864)*	*265*	*72*
**Wales - types of recipient**	**Value (%)**	**Payments - n (%)**	**Median - £ (IQR)**	**Recipients - n (%)**	**Pharmaceutical companies (%)**
Healthcare commissioning, planning and regulatory organisations	**920,980.22 (46.38)**	**557 (56.61)**	225 (114.24 - 486.39)	10 (4.72)	**50 (78.13)**
Private companies other than providers of health services	179,495.4 (9.04)	56 (5.69)	**1475 (216 - 6,600)**	10 (4.72)	8 (12.50)
Education and research providers	179,256.38 (9.03)	37 (3.76)	336 (175.2 - 1000)	5 (2.36)	16 (25.00)
Public sector primary care providers	173,268.30 (8.73)	141 (14.33)	800 (434.5 - 1,152)	**118 (55.66)**	15 (23.44)
Private sector healthcare providers	153,983.36 (7.76)	18 (1.83)	440 (360.94 - 1,732)	6 (2.83)	9 (14.06)
Public administration and providers of public services	108,000 (5.44)	1 (0.10)	-	1 (0.47)	1 (1.56)
Patient organisation	99,784.32 (5.03)	22 (2.24)	747.93 (500 - 2,000)	10 (4.72)	11 (17.19)
Public sector secondary and tertiary care providers	96,862.66 (4.88)	20 (2.03)	253.66 (200 - 954)	3 (1.42)	13 (20.31)
Professional organisations	64,181.82 (3.23)	88 (8.94)	400 (280 - 800)	38 (17.92)	31 (48.44)
Charities and other third-sector organisations	5,036.8 (0.25)	17 (1.73)	120 (120 - 180)	4 (1.89)	7 (10.94)
Formal bodies representing healthcare professionals or patients	4,679.37 (0.24)	27 (2.74)	120 (96 - 142)	7 (3.30)	11 (17.19)
All payments	*1,987,702.62*	*984*	*300 (144 - 800)*	*212*	*64*

For peer review only

For peer review only - http://bmjopen.bmj.com/site/about/guidelines.xhtml

**Northern Ireland - types of recipient**	**Value (%)**	**Payments - n (%)**	**Median - £ (IQR)**	**Recipients - n (%)**	**Pharmaceutical companies (%)**
Public sector primary care providers	**184,903.09 (35.72)**	**127 (40.97)**	600 (434.5 - 1,600)	**94** (60.65)	6 (14.29)
Public sector secondary and tertiary care providers	111,743.45 (21.59)	83 (26.77)	288 (163.4 - 490.13)	5 (3.23)	**27 (64.29)**
Professional organisations	81,489.7 (15.74)	34 (10.97)	600 (320 - 1,784)	21 (13.55)	21 (50.00)
Patient organisation	43,205.6 (8.35)	15 (4.84)	650 (600 - 1,450)	7 (4.52)	14 (33.33)
Education and research providers	32,258 (6.23)	10 (3.23)	1100 (873.75 - 3525)	1 (0.65)	7 (16.67)
Private companies other than providers of health services	26,242.77 (5.07)	6 (1.94)	**4179.38 (1,152.19 - 7,687.5)**	4 (2.58)	3 (7.14)
Healthcare commissioning, planning and regulatory organisations	22,447.6 (4.34)	7 (2.26)	1500 (470.8 - 4,087)	4 (2.58)	6 (14.29)
Private sector healthcare providers	11,476.85 (2.22)	23 (7.42)	38.49 (28.9 - 485)	16 (10.32)	6 (14.29)
Formal bodies representing healthcare professionals or patients	2,133.34 (0.41)	2 (0.65)	1066.67 (933.33 - 1200.00)	1 (0.65)	1 (2.38)
Alternative providers of health services	1,700 (0.33)	3 (0.97)	600 (550 - 600)	2 (1.29)	2 (4.76)
All payments	*517,600.40*	*310*	*475.2 (217.25 - 1,357.47)*	*155*	*42*

For peer review only

Supplementary File 8. Top 10 recipients in each country

Country	Recipient	Type of recipient	Value - £	Payments - n	Companies - n
England	King's College London	Education and research providers	2,572,086.51	45	18
	Bladder and Bowel Foundation	Patient organisation	1,459,371.52	11	1
	London School Hyg and Tropical Med	Education and research providers	935,025.98	16	6
	PeerVoice	Private companies other than providers of health services	930,028.30	11	3
	University College London	Education and research providers	907,256.40	96	36
	Diabetes UK - England	Patient organisation	888,845.00	41	7
	Healthcare At Home	Private sector healthcare providers	872,740.81	18	2
	Cancer Research UK	Patient organisation	804,543.76	19	9
	Central Manchester Univ Hosps FT	Public sector secondary and tertiary care providers	739,595.97	108	37
	British Society for Rheumatology	Professional organisations	543,012.33	31	14
Scotland	Quintiles - Scotland	Private companies other than providers of health services	682,601.65	5	1
	Myeloma UK	Patient organisation	521,574.36	12	7
	NHS Greater Glasgow and Clyde	Healthcare commissioning, planning and regulatory organisations	483,354.99	153	34
	University of Glasgow	Education and research providers	442,707.63	70	26
	University of Dundee	Education and research providers	160,632.40	20	11
	NHS Lothian	Healthcare commissioning, planning and regulatory organisations	144,175.05	73	25
	University of Edinburgh	Education and research providers	73,014.39	37	21
	NHS Tayside	Healthcare commissioning, planning and regulatory organisations	67,924.08	74	24
	NHS Ayrshire and Arran	Healthcare commissioning, planning and regulatory organisations	63,276.93	48	25
	Digestive Disorders Federation	Professional organisations	60,796.00	2	2
Wales	Cardiff and Vale University HB	Healthcare commissioning, planning and regulatory organisations	344,131.95	89	28

For peer review only

	Abertawe Bro Morgannwg Univ HB	Healthcare commissioning, planning and regulatory organisations	242,418.82	124	32
	LloydsPharmacy	Private sector healthcare providers	146,376.00	4	1
	University of Cardiff	Education and research providers	120,822.78	27	13
	Bluebay Medical Systems	Private companies other than providers of health services	116,900.00	26	1
	Hywel Dda University HB	Healthcare commissioning, planning and regulatory organisations	115,600.62	77	27
	National Assembly for Wales	Public administration and providers of public services	108,000.00	1	1
	Betsi Cadwaladr University HB	Healthcare commissioning, planning and regulatory organisations	101,352.58	76	21
	Cwm Taf University Health Board	Healthcare commissioning, planning and regulatory organisations	84,624.15	119	25
	Velindre NHS Trust	Public sector secondary and tertiary care providers	80,629.32	18	13
Northern Ireland	Belfast Health and SC Trust	Public sector secondary and tertiary care providers	60,615.65	30	17
	Federation Of Family Practices	Public sector primary care providers	40,235.20	3	3
	UK and Ireland Society of Cataract and Refractive Surgeons - Northern Ireland	Professional organisations	35,000.00	1	1
	Queen's University Belfast	Education and research providers	32,258.00	10	7
	Northern Health and SC Trust	Public sector secondary and tertiary care providers	23,703.46	14	8
	Medical Communications	Private companies other than providers of health services	23,250.00	3	1
	Adult ADHD - Northern Ireland	Patient organisation	20,000.00	1	1
	Ulster Chemists' Association	Professional organisations	16,584.00	4	3
	Western Health and SC Trust	Public sector secondary and tertiary care providers	15,068.55	12	10
	Cancer Focus Northern Ireland	Patient organisation	12,255.60	2	2

For peer review only

Supplementary File 9. Post-hoc Bonferroni pairwise comparisons between countries of payments per recipient type

Recipient type	Group 1 – Group 2*	Test Statistic	Std. Error	Std. Test Statistic	Sig.	Adj. Sig†,‡	Median (IQR) – group 1 - £	Median (IQR) – group 2 - £
Alternative providers of health services	England-Scotland	-59.003	22.681	-2.601	0.009	**0.028**	200 (160 – 394)	700 (360 – 1,100)
	England-Northern Ireland	-65.919	31.816	-2.072	0.038	0.115	200 (160 – 394)	600 (550 - 600)
	Scotland-Northern Ireland	6.917	38.645	0.179	0.858	1	700 (360 – 1,100)	600 (550 - 600)
Charities and other third-sector organisations	England-Scotland	-118.214	56.132	-2.106	0.035	0.106	223.52 (157 – 487.2)	**1700 (377.5 - 6,250)**
	Wales-England	79.844	27.702	2.882	0.004	**0.012**	120 (120 - 180)	223.52 (157 – 487.2)
	Wales-Scotland	198.059	62.049	3.192	0.001	**0.004**	120 (120 - 180)	**1700 (377.5 - 6,250)**
Education and research providers	England-Scotland	-5.623	27.857	-0.202	0.84	1	1000 (333.34 – 4,798.40)	1152 (400 – 2,880)
	England-Northern Ireland	-92.61	97.628	-0.949	0.343	1	1000 (333.34 – 4,798.40)	1100 (873.75 - 3525)
	Scotland-Northern Ireland	86.987	100.458	0.866	0.387	1	1152 (400 – 2,880)	1100 (873.75 - 3525)
	Wales-England	204.193	51.523	3.963	<.001	**0**	336 (175.2 - 1000)	1000 (333.34 – 4,798.40)
	Wales-Scotland	209.816	56.703	3.7	<.001	**0.001**	336 (175.2 - 1000)	1152 (400 – 2,880)
	Wales-Northern Ireland	296.803	109.409	2.713	0.007	**0.04**	336 (175.2 - 1000)	1100 (873.75 - 3525)
Formal bodies representing healthcare professionals	England-Scotland	-46.978	99.854	-0.47	0.638	1	200 (160 – 259.8)	213.6 (211.4 - 214.8)
	England-Northern Ireland	-223.228	99.854	-2.236	0.025	0.152	200 (160 – 259.8)	1066.67 (933.33 - 1200.00)
	Scotland-Northern Ireland	176.25	140.907	1.251	0.211	1	213.6 (211.4 - 214.8)	1066.67 (933.33 - 1200.00)
	Wales-England	138.133	27.905	4.95	<.001	**0**	120 (96 - 142)	200 (160 – 259.8)
	Wales-Scotland	185.111	103.261	1.793	0.073	0.438	120 (96 - 142)	213.6 (211.4 - 214.8)

For peer review only

For peer review only - http://bmjopen.bmj.com/site/about/guidelines.xhtml

	Wales-Northern Ireland	361.361	103.261	3.5	<.001	**0.003**	120 (96 - 142)	1066.67 (933.33 - 1200.00)
Healthcare commissioning, planning and regulatory organisations	England-Scotland	-122.159	44.623	-2.738	0.006	**0.037**	208.17 (160 – 307.2)	240 (131.18 - 500)
	England-Northern Ireland	-786.684	361.817	-2.174	0.03	0.178	208.17 (160 – 307.2)	1500 (470.8 - 4,087)
	Scotland-Northern Ireland	664.525	363.4	1.829	0.067	0.405	240 (131.18 - 500)	1500 (470.8 - 4,087)
	Wales-Scotland	94.65	56.651	1.671	0.095	0.569	225 (114.24 - 486.39)	240 (131.18 - 500)
	Wales-Northern Ireland	759.175	363.497	2.089	0.037	0.22	225 (114.24 - 486.39)	1500 (470.8 - 4,087)
	England-Wales	-27.51	45.405	-0.606	0.545	1	208.17 (160 – 307.2)	225 (114.24 - 486.39)
Patient organisation	Wales-England	144.727	77.321	1.872	0.061	0.367	747.93 (500 - 2,000)	**4000 (500 – 11,104)**
	Wales-Scotland	29.422	91.028	0.323	0.747	1	747.93 (500 - 2,000)	1000 (253.68 - 9,745)
	Northern Ireland-Wales	-8.633	118.686	-0.073	0.942	1	650 (600 - 1,450)	747.93 (500 - 2,000)
	Northern Ireland-Scotland	-38.055	103.121	-0.369	0.712	1	650 (600 - 1,450)	1000 (253.68 - 9,745)
	Northern Ireland-England	153.361	91.248	1.681	0.093	0.557	650 (600 - 1,450)	**4000 (500 – 11,104)**
	Scotland-England	115.306	50.259	2.294	0.022	0.131	1000 (253.68 - 9,745)	**4000 (500 – 11,104)**
Private companies other than providers of health services	England-Scotland	-317.041	89.112	-3.558	<.001	**0.002**	300 (196.8 – 598.92)	1,200 (350 - 5,528.88)
	England-Northern Ireland	-527.308	180.717	-2.918	0.004	**0.021**	300 (196.8 – 598.92)	**4179.38 (1,152.19 - 7,687.5)**
	Scotland-Northern Ireland	210.267	200.821	1.047	0.295	1	1,200 (350 - 5,528.88)	**4179.38 (1,152.19 - 7,687.5)**
	Wales-Scotland	115.061	106.256	1.083	0.279	1	**1475 (216 - 6,600)**	1,200 (350 - 5,528.88)
	Wales-Northern Ireland	325.327	189.758	1.714	0.086	0.519	**1475 (216 - 6,600)**	**4179.38 (1,152.19 - 7,687.5)**

For peer review only - http://bmjopen.bmj.com/site/about/guidelines.xhtml

	England-Wales	-201.98	60.166	-3.357	<.001	**0.005**	300 (196.8 – 598.92)	**1475 (216 - 6,600)**
Private sector healthcare providers	England-Scotland	-64.982	21.355	-3.043	0.002	**0.014**	240 (166 – 588)	647.54 (206.65 - 1,500)
	England-Wales	-88.107	39.755	-2.216	0.027	0.16	240 (166 – 588)	440 (360.94 - 1,732)
	Northern Ireland-Wales	-206.778	52.076	-3.971	<.001	**0**	38.49 (28.9 - 485)	440 (360.94 - 1,732)
	Northern Ireland-Scotland	-183.652	39.843	-4.609	<.001	**0**	38.49 (28.9 - 485)	647.54 (206.65 - 1,500)
	Northern Ireland-England	118.671	35.352	3.357	<.001	**0.005**	38.49 (28.9 - 485)	240 (166 – 588)
	Scotland-wales	-23.126	43.797	-0.528	0.597	1	647.54 (206.65 - 1,500)	440 (360.94 - 1,732)
Professional organisations	England-Northern Ireland	-35.169	109.4	-0.321	0.748	1	500 (240 – 3,200)	600 (320 - 1,784)
	Scotland-Northern Ireland	157.615	114.524	1.376	0.169	1	450 (285.67 - 980)	600 (320 - 1,784)
	Wales-England	182.447	69.008	2.644	0.008	**0.049**	400 (280 - 800)	500 (240 – 3,200)
	Wales-Scotland	60.001	76.873	0.781	0.435	1	400 (280 - 800)	450 (285.67 - 980)
	Wales-Northern Ireland	217.616	127.597	1.705	0.088	0.529	400 (280 - 800)	600 (320 - 1,784)
	Scotland-England	122.446	39.962	3.064	0.002	**0.013**	450 (285.67 - 980)	500 (240 – 3,200)
Public sector primary care providers	England-Northern Ireland	-355.779	76.292	-4.663	<.001	**0**	434.5 (193.6 – 869)	600 (434.5 - 1,600)
	Scotland-Northern Ireland	407.76	105.06	3.881	<.001	**0.001**	434.5 (202.23 - 651,75)	600 (434.5 - 1,600)
	England-Wales	-459.79	72.597	-6.333	<.001	**0**	434.5 (193.6 – 869)	800 (434.5 - 1,152)
	Northern Ireland-Wales	-104.011	102.62	-1.014	0.311	1	600 (434.5 - 1,600)	800 (434.5 - 1,152)
	Scotland-England	51.98	76.008	0.684	0.494	1	434.5 (202.23 - 651,75)	434.5 (193.6 – 869)
	Scotland-wales	-511.771	102.409	-4.997	<.001	**0**	434.5 (202.23 - 651,75)	800 (434.5 - 1,152)

*Each row tests the null hypothesis that the Sample 1 and Sample 2 distributions are the same.
†Asymptotic significances (2-sided tests) are displayed. The significance level is .050.
‡Significance values have been adjusted by the Bonferroni correction for multiple tests

For peer review only - http://bmjopen.bmj.com/site/about/guidelines.xhtml

Supplementary File 10. Post-hoc Bonferroni pairwise comparisons of payment types between countries

Payment Type*	Group 1 – Group 2**	Test Statistic	Std. Error	Std. Test Statistic	Sig.	Adj. Sig†,‡	Median (IQR) – group 1 - £	Median (IQR) – group 2 - £
Contribution to costs of Events	Wales-England	498.254	159.743	3.119	0.002	0.011	223.36 (120 - 400)	240 (155.95 - 400)
	Wales-Scotland	1449.82	208.45	6.955	<.001	0	223.36 (120 - 400)	300 (160 - 600)
	Wales-Northern	2690.485	402.839	6.679	<.001	0	223.36 (120 - 400)	477.6 (200 - 1,147.6)
	England-Scotland	-951.566	143.605	-6.626	<.001	0	240 (155.95 - 400)	300 (160 - 600)
	England-Northern	-2192.23	373.43	-5.871	<.001	0	240 (155.95 - 400)	477.6 (200 - 1,147.6)
	Scotland-Northern	1240.664	396.716	3.127	0.002	0.011	300 (160 - 600)	477.6 (200 - 1,147.6)
Donations and Grants	Wales-England	25.151	94.77	0.265	0.791	1	800 (434.5 - 2,200)	959.98 (256 - 4,800)
	Northern-Scotland	-140.935	137.745	-1.023	0.306	1	434.5 (217.5 - 1,867.5)	651.75 (217.25 - 2,578)
	Northern-Wales	-451.347	142.863	-3.159	0.002	0.009	434.5 (217.5 - 1,867.5)	800 (434.5 - 2,200)
	Northern-England	476.498	111.315	4.281	<.001	0	434.5 (217.5 - 1,867.5)	959.98 (256 - 4,800)
	Scotland-Wales	-310.412	124.755	-2.488	0.013	0.077	651.75 (217.25 - 2,578)	800 (434.5 - 2,200)
	Scotland-England	335.563	86.862	3.863	<.001	0.001	651.75 (217.25 - 2,578)	959.98 (256 - 4,800)

*Kruskal-Wallis results: Contributions to cost of events $\chi2(3) = 89.680$, p = .000; Donations and grants $\chi2(3) = 31.698$, p = <.000

**Each row tests the null hypothesis that the Sample 1 and Sample 2 distributions are the same.

†Asymptotic significances (2-sided tests) are displayed. The significance level is .050.

‡Significance values have been adjusted by the Bonferroni correction for multiple tests.

For peer review only

For peer review only - http://bmjopen.bmj.com/site/about/guidelines.xhtml

STROBE Statement—Checklist of items that should be included in reports of ***cross-sectional studies***

	Item No	Recommendation	Page No
**Title and abstract**	1	(*a*) Indicate the study's design with a commonly used term in the title or the abstract	2
		(*b*) Provide in the abstract an informative and balanced summary of what was done and what was found	2
**Introduction**			
Background/rationale	2	Explain the scientific background and rationale for the investigation being reported	3-4
Objectives	3	State specific objectives, including any prespecified hypotheses	4
**Methods**			
Study design	4	Present key elements of study design early in the paper	5
Setting	5	Describe the setting, locations, and relevant dates, including periods of recruitment, exposure, follow-up, and data collection	5
Participants	6	(*a*) Give the eligibility criteria, and the sources and methods of selection of participants	5
Variables	7	Clearly define all outcomes, exposures, predictors, potential confounders, and effect modifiers. Give diagnostic criteria, if applicable	5-6
Data sources/ measurement	8*	For each variable of interest, give sources of data and details of methods of assessment (measurement). Describe comparability of assessment methods if there is more than one group	5-6
Bias	9	Describe any efforts to address potential sources of bias	n/a
Study size	10	Explain how the study size was arrived at	N/a
Quantitative variables	11	Explain how quantitative variables were handled in the analyses. If applicable, describe which groupings were chosen and why	5
Statistical methods	12	(*a*) Describe all statistical methods, including those used to control for confounding	5
		(*b*) Describe any methods used to examine subgroups and interactions	n/a
		(*c*) Explain how missing data were addressed	n/a
		(*d*) If applicable, describe analytical methods taking account of sampling strategy	n/a
		(*e*) Describe any sensitivity analyses	n/a
**Results**			
Participants	13*	(a) Report numbers of individuals at each stage of study—eg numbers potentially eligible, examined for eligibility, confirmed eligible, included in the study, completing follow-up, and analysed	Supplementary File 4 (flow diagram)
		(b) Give reasons for non-participation at each stage	n/a
		(c) Consider use of a flow diagram	Supplementary File 4

For peer review only

Descriptive data	14*	(a) Give characteristics of study participants (eg demographic, clinical, social) and information on exposures and potential confounders	n/a
		(b) Indicate number of participants with missing data for each variable of interest	n/a
Outcome data	15*	Report numbers of outcome events or summary measures	6
Main results	16	(*a*) Give unadjusted estimates and, if applicable, confounder-adjusted estimates and their precision (eg, 95% confidence interval). Make clear which confounders were adjusted for and why they were included	n/a
		(*b*) Report category boundaries when continuous variables were categorized	n/a
		(*c*) If relevant, consider translating estimates of relative risk into absolute risk for a meaningful time period	n/a
Other analyses	17	Report other analyses done—eg analyses of subgroups and interactions, and sensitivity analyses	n/a
**Discussion**			
Key results	18	Summarise key results with reference to study objectives	11-13
Limitations	19	Discuss limitations of the study, taking into account sources of potential bias or imprecision. Discuss both direction and magnitude of any potential bias	13
Interpretation	20	Give a cautious overall interpretation of results considering objectives, limitations, multiplicity of analyses, results from similar studies, and other relevant evidence	11-14
Generalisability	21	Discuss the generalisability (external validity) of the study results	11-14
**Other information**			
Funding	22	Give the source of funding and the role of the funders for the present study and, if applicable, for the original study on which the present article is based	1

*Give information separately for exposed and unexposed groups.

Note: An Explanation and Elaboration article discusses each checklist item and gives methodological background and published examples of transparent reporting. The STROBE checklist is best used in conjunction with this article (freely available on the Web sites of PLoS Medicine at http://www.plosmedicine.org/, Annals of Internal Medicine at http://www.annals.org/, and Epidemiology at http://www.epidem.com/). Information on the STROBE Initiative is available at www.strobe-statement.org.

For peer review only
